# Substituted Cyclopentannulated Tetraazapentacenes

**DOI:** 10.1002/chem.202201842

**Published:** 2022-09-19

**Authors:** Steffen Maier, Robin Heckershoff, Nikolai Hippchen, Kerstin Brödner, Frank Rominger, Jan Freudenberg, A. Stephen K. Hashmi, Uwe H. F. Bunz

**Affiliations:** ^1^ Organisch-Chemisches Institut (OCI) Heidelberg University Im Neuenheimer Feld 270 69120 Heidelberg Germany; ^2^ Chemistry Department Faculty of Science King Abdulaziz University Jeddah 21589 Saudi Arabia; ^3^ Centre for Advanced Materials (CAM) Heidelberg University Im Neuenheimer Feld 225 69120 Heidelberg Germany

**Keywords:** cyclization, dihydroazaacenes, postfunctionalization, semiconductors, solid-state packing

## Abstract

Brominated pentannulated dihydrotetraazapentacenes were prepared by gold‐ or palladium‐catalyzed 5‐*endo*‐*dig* cyclization of TIPS‐ethynylated dihydrotetraazaacenes (TIPS = tri*iso*propylsilyl). Post‐functionalization was demonstrated by Sonogashira alkynylation and Rosenmund‐von Braun cyanation. Calculations predict these species to act as n‐type semiconductors, which was verified for two derivates through characterization in organic field‐effect transistors.

## Graphical abstract

**The introduction of bromine** substituents into cyclopentannulated tetraazapentacenes allows post‐functionalization, for example through Sonogashira coupling and Rosenmund‐von Braun reaction. The altered properties of the resulting new compounds were examined by experimental and computational methods, and the potential use as organic field‐effect transistors was demonstrated.

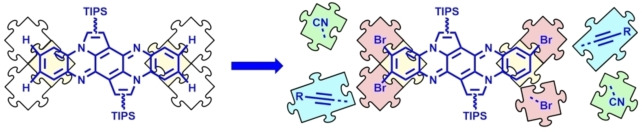

## Introduction

**Scheme 1:**
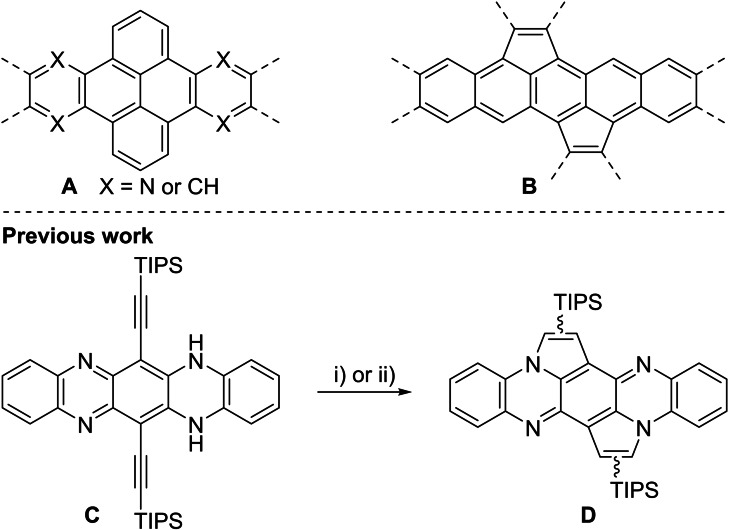
Top: Examples of the stabilization of (aza‐)acenes by annulation. Bottom: Synthesis of non‐halogenated cyclopentannulated dihydrotetraazapentacenes. Conditions: i) IPrAuNTf_2_ (10 mol%), DCE, 80 °C, 16 h; ii) PdCl_2_ (10 mol%), MeCN/CHCl_3_ 1 : 1, 80 °C, 18 h.

In the last two decades, *N*‐heteroacenes have attracted increasing attention in the field of organic electronics. Their low band gap combined with high physical and chemical stability favor their use in devices such as organic field‐effect transistors (OFETs).^[1]^ With increasing number of annulated benzene rings, stability of acenes and *N*‐heteroacenes becomes a dramatic issue. Larger acenes are prone to (photo)oxidation due to increasing HOMO energies and dimerize more easily.^[2]^ Several strategies, such as steric protection or encapsulation, suppress these undesired degradation reactions of larger acenes.^[3]^ Other approaches include (formal) annulation reactions on the acene backbone, increasing the number of Clar sextets and stabilizing the aromatic system.[^4^, ^5^] Whereas benzannulation (**A**) and cyclopentannulation (**B**) are established for acenes, examples of cyclopentannulation on larger *N*‐heteroacenes or benzannulations including a nitrogen atom have barely explored (Scheme 1).

Recently, we developed a transition metal‐catalyzed cycloisomerization of bissilylethynylated *N*,*N’*‐dihydrotetraazapentacene (**C**).^[6]^ We disclosed a catalyst‐dependent (gold^[7]^ and palladium) silyl shift furnishing three different regioisomers of the annulated tetraazapentacenes (**D**) with similar photophysical properties but dramatic differences in crystal packing. Within that work, a detailed experimental and computational mechanistic investigation was conducted to explain the observed difference in the shift‐behavior between gold and palladium catalysts.

Attaching substituents to acenes is a useful tool to alter their photophysical, electronic, physicochemical, and/or morphological properties and to tailor materials for a specific application.^[8]^ These changes are pronounced; the addition of strongly electron‐withdrawing groups (like fluorine or cyano) transforms typical p‐type semico01nductor materials into compounds suitable for electron transport.^[9]^

## Results and Discussion

**Scheme 2:**
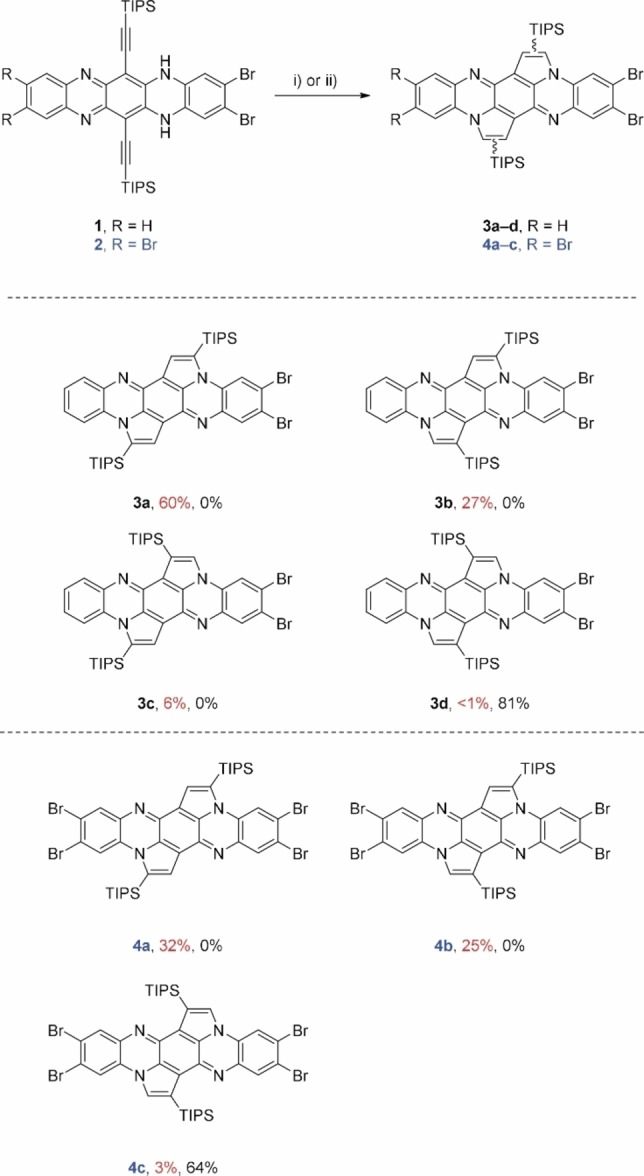
Synthesis of brominated cyclopentannulated tetraazapentacenes **3 a**–**d** and **4 a**–**c**. Conditions: i) IPrAuNTf_2_ (10 mol%), DCE, 80 °C, 2 d; ii) PdCl_2_ (10 mol%), MeCN/CHCl_3_ (1 : 1), 80 °C, 18 h. Yields in red: gold catalysis; yields in black: palladium catalysis.

In this article, we report the synthesis of halogenated cyclopentannulated arenes derived from **D**, furnishing annulated species ready for postfunctionalization, for example, by cross‐coupling reactions. The brominated dihydrotetraazapentacenes **1** ^[10]^ and **2** ^[11]^ serve as excellent starting materials for our endeavor (Scheme 2).

Consistent with our previous results, gold‐catalyzed transformation of dihydroazaacenes **1** or **2** (Scheme 2) resulted in a mixture of regioisomers, in which no (**3 a**, **4 a**) or one silyl group was shifted (**3 b**, **3 c**, **4 b**). For the dibromo derivative **1**, two isomers, in which one silyl group underwent a [1,2] shift, formed as a consequence of the symmetry of the starting material. All isomers were separated by flash column chromatography (for **3 a** and **3 b** additional preparative HPLC) and characterized by NMR spectroscopy and single crystal X‐ray analysis.

Palladium catalysis (Scheme 2) resulted exclusively in the formation of the doubly shifted isomers **3 d** and **4 c**. The products were purified by column chromatography for microscale or by precipitation out of DCM and a second time out of THF (for more details see the Supporting Information) for gram‐scale synthesis. The solubility in common organic solvents distinctly depends on the number of bromine atoms as well as on the symmetry of the molecule. With increasing number of halogens and increasing symmetry, solubility decreases (e. g., **3 a**: 34.5 mg mL^−1^, **4 c**: 0.5 mg mL^−1^), hampering characterization by ^13^C NMR for **4 a**/**c**, even at elevated temperatures. Measurements of cyclic voltammetry failed due to the low solubility of the compounds.

**Scheme 3:**
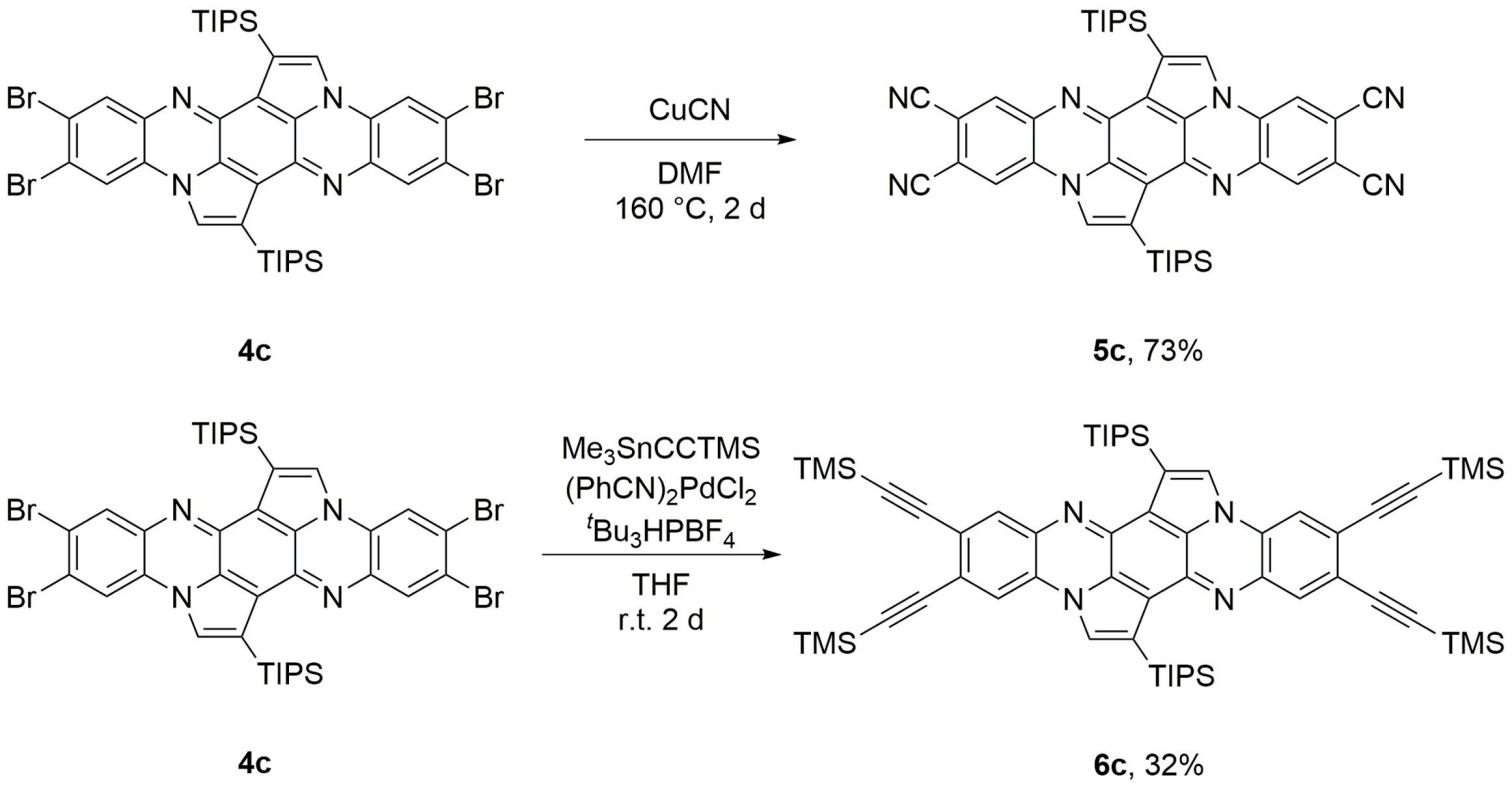
Post‐functionalization of **4 c** by cyanation or alkynylation.

Easily prepared **4 c** was postfunctionalized by a fourfold Rosenmund‐von Braun reaction^[12]^ using copper cyanide in DMF to furnish **5 c** in 73 % yield (92 % per coupling, Scheme 3, top). **5 c** is of interest as tetracyanotetraazapentacene is elusive. Stille coupling of **5 c** with trimethylsilylethinylstannane gives well‐soluble **6 c** as an orange solid in 32 % yield (Scheme 3, bottom).

**Figure 1:**
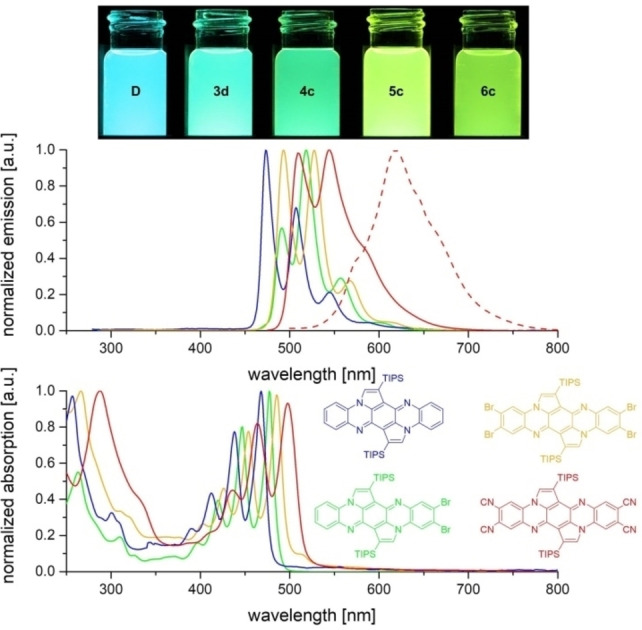
Top: Photographs of **D**, **3 d**, **4 c**, **5 c** and **6 c** in DCM under irradiation by UV light (365 nm). Middle: Normalized emission spectra of **D**, **3 d**, **4 c** and **5 c** in DCM (solid line) and of **5 c** as powder (dashed line). Bottom: Normalized absorption spectra of **D**, **3 d**, **4 c** and **5 c** in DCM.

The absorption and emission spectra of the regioisomers (**3 a**–**d** or **4 a**–**c**) are nearly identical (Table 1, Figure 1; see Figures S17–S24 in the Supporting Information for all spectra). Cyclization of the starting materials **1** and **2** results in a blue shift of 77–80 nm for the dibromo and 60–64 nm for the tetrabromo derivatives due to the non‐aromatic *para*‐diquinone center, supported by NICS_zz_ XY scans (Figure S52). The absorption maximum of **3 d** is red‐shifted by 11 nm (**D**: 466 nm, **3 d**: 477 nm) compared to that of the unsubstituted compound. Functionalization with two additional bromine atoms (**4 c**) shifts the absorption maxima bathochromically by 8 nm (**3 d**: 477 nm, **4 c**: 485 nm). The absorption maximum of the tetraalkynylated **6 c** is red‐shifted by 26 nm. Substitution of the bromines by cyanides results in a red shift of 13 nm (**4 c**: 485 nm, **5 c**: 498 nm) due to their electron‐withdrawing effect and extension of the π‐system. The LUMO level concomitantly decreases from −2.92 to −3.78 eV, making **5 c** suitable for the application in n‐type OFETs. The emission spectra are mirror‐like images of the absorption spectra and show similar trends for the influence of the substituents. Stokes shifts are generally small with values between 190 and 569 cm^−1^. Fluorescence quantum yields in DCM solutions are 79 % for **3 d**, 65 % for **4 c** and 43 % for **5 c**. In contrast to all other derivatives, tetra‐cyanated **5 c** shows solid state fluorescence as well and the emission spectrum is red‐shifted by about 60–70 nm in comparison to the measurement in solution (DCM). In accordance to our preceding work with **D**, the cyclopentannulation of the tetraazapentacene core structure is quite substantial. The reference compound 6,13‐bis(triisopropylsilylethynyl)tetraazapentacene (TIPS‐TAP) and its tetra‐brominated analogue^[11]^ exhibit the longest absorption maxima around 680 and 720 nm, respectively. This equals to a blue‐shift for the cyclopentannulated compounds **D**, **3** and **4** of more than 200 nm and an increase of the optical gap by about 1 eV. In contrast to our study, in which the cyclopentannulation results in a blue‐shift, cyclopentannulated pentacenes are generally red‐shifted in comparison to related pentacene derivatives as seen in works by the groups of Plunkett and Chi.[^5^, ^13^] The major difference compared to our system is the decreased aromaticity of the cyclopentannulated tetraazapentacenes caused by the tri‐substituted nitrogens enforcing a quinoidal structure.

**Table 1:** Experimental (DCM solutions) and calculated (gas‐phase) properties of **1**–**6** and **D**.

	*λ*_max,abs_ [nm]	*λ*_max,em_ [nm]	HOMO^[a]^ [eV]	LUMO^[a]^ [eV]	Gap meas^[b]^/calcd^[a]^ [eV]
**1**	554	547/575	−5.47 (−5.43)^[c]^	−2.64 (−2.71)^[c]^	2.24/2.83 (2.72)^[c]^
**2**	545	569/610	−5.64	−2.90	2.27/2.70
**D** ^[d]^	466	473/507/545	−5.48	−2.45	2.66/3.03
**3 a**	474	488/516/552	−5.60	−2.58	2.62/3.02
**3 b**	476	484/517/554	−5.63	−2.63	2.60/3.00
**3 c**	475	484/517/554	−5.64	−2.64	2.61/3.00
**3 d**	477	491/518/557	−5.67	−2.70	2.60/2.97
**4 a**	481	492/524/561	−5.77	−2.81	2.58/2.96
**4 b**	484	492/525/565	−5.81	−2.86	2.56/2.95
**4 c**	485	493/526/567	−5.85	−2.92	2.56/2.93
**5 c**	498	509/545	−6.66	−3.78	2.49/2.88
**6 c**	511	516/555/600	−5.52	−2.77	2.43/2.75

[a] Obtained from DFT calculations (Gaussian16,^[14]^ B3LYP/def2‐TZVP). [b] Optical gap estimated from *λ*_maxt,abs_: *E*_g(opt)_=1239.8/*λ*_max,abs_. [c] Value in parenthesis for **1 iso** (tautomer of **1**), see the Supporting Information. [d] Values for the doubly shifted isomer are shown.

**Figure 2:**
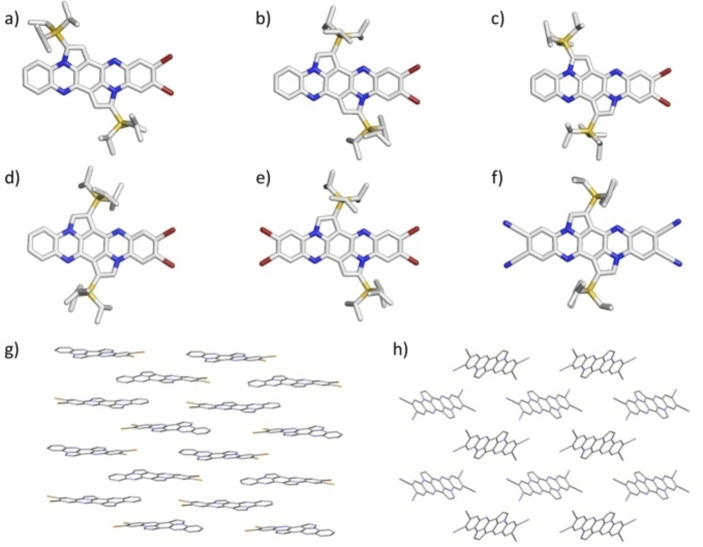
Single crystal structures of a) **3 a**, b) **3 b**, c) **3 c**, d) **3 d**, e) **4 b**, f) **5 c** and crystal structure packing of g) **3 d** and h) **5 c**. For the single molecules, hydrogen atoms were omitted for clarity. For crystal packing TIPS‐groups and co‐crystallized solvents were also omitted.

In contrast to optoelectronics, the silyl regioisomerism dramatically affects the packing in the solid state, which is supported by the differences in the unit dimensions (Table 2). Figure 2 displays the crystal structures of **3 a**–**d**, **4 b** and **5 c** (see Figure S35 for the packing of **3 a**–**c** and **4 b**).^[15]^ **3 a** co‐crystallized with methanol and chloroform. **3 b** formed a one dimensional staircase of dimers and crystallized without solvent intercalation. The distances between the molecules amount to 347 pm and 386 pm. Within the dimer pairs, π–π contacts over three rings are evident. The solid‐state structure of **3 c** includes one molecule of methanol H‐bonded to the pyrazinic nitrogen. **3 c** packed in a one‐dimensional staircase packing of dimers. The distance within the dimers (π–π overlap of the two terminal rings) amounts to 337 pm, whereas the inter‐dimer distance comprises 307 pm as a consequence of their lateral off‐set.

**Table 2:** Unit cell dimensions of the crystal structures of **3 a**–**d**, **4 b** and **5 c**.

		
	*a* [Å]	*b* [Å]	*c* [Å]	*α*[°]	*β*[°]	*γ*[°]
**3 a**	8.85	16.09	16.72	70.47	81.31	89.41
**3 b**	8.31	13.48	17.61	100.68	91.14	90.07
**3 c**	8.08	14.80	18.19	70.13	88.39	81.64
**3 d**	14.97	19.72	28.65	90	90	90
**4 b**	8.07	14.85	18.76	73.52	87.51	82.25
**5 c**	16.95	12.64	23.29	90	99.92	90

Compound **3 d** crystallized with chloroform – its π‐system is not fully planar. Nevertheless, the molecule formed a brick‐wall‐like packing. The outer two rings of each molecule overlap. **4 b** formed dimer pairs in the solid state with distances between the molecules of 344 pm. The distance between the dimer pairs was estimated to be 367 pm. Co‐crystallized methanol forms H‐bonds to the pyrazinic nitrogen atoms. The acene units of the two independent molecules in **5 c** have an angle of 80.2° between each other and pack nearly edge‐to‐face. They build up a 3D pattern with no π–π contacts.

From the crystal structures we extracted all different types of neighboring molecular pairs for which we calculated transfer integrals using the ADF software package^[16]^ and reorganization energies using the Gaussian 16 software package.^[14]^ These values were then used for the calculation of a theoretical electron transport mobility *μ* (Table 3).^[14]^ Only the highest transfer integral of each compound is shown – all other transfer integrals, including images of the corresponding dimer pairs, are listed in Figures S46–S51. As expected, for the derivatives exhibiting π–π stacking (**3 b**–**d**, **4 b**), the transfer integrals as well as the calculated mobilities are the highest. They are close to or exceed 1 cm^2^ V^−1^ s^−1^; **3 c** is predicted to perform best (*μ*_electron_=4.2 cm^2^ V^−1^ s^−1^).

**Table 3:** Calculated transfer integrals, reorganization energies and electron mobilities for an electron transport.

	Transfer integral [eV]	Reorganization energy [eV]	Electron transfer rate [s^−1^]	*μ*_electron_ [cm^2^ V^−1^ s^−1^]
**3 a**	7.84×10^−2^	2.78×10^−1^	6.24×10^13^	2.4
**3 b**	4.06×10^−2^	2.82×10^−1^	1.66×10^13^	6.0×10^−1^
**3 c**	1.01×10^−1^	2.84×10^−1^	1.02×10^14^	4.2
**3 d**	5.28×10^−2^	2.88×10^−1^	2.77×10^13^	1.2
**4 b**	8.44×10^−2^	2.80×10^−1^	7.18×10^13^	2.9
**5c**	3.24×10^−3^	2.71×10^−1^	1.08×10^11^	1.7×10^−2^

**Figure 3:**
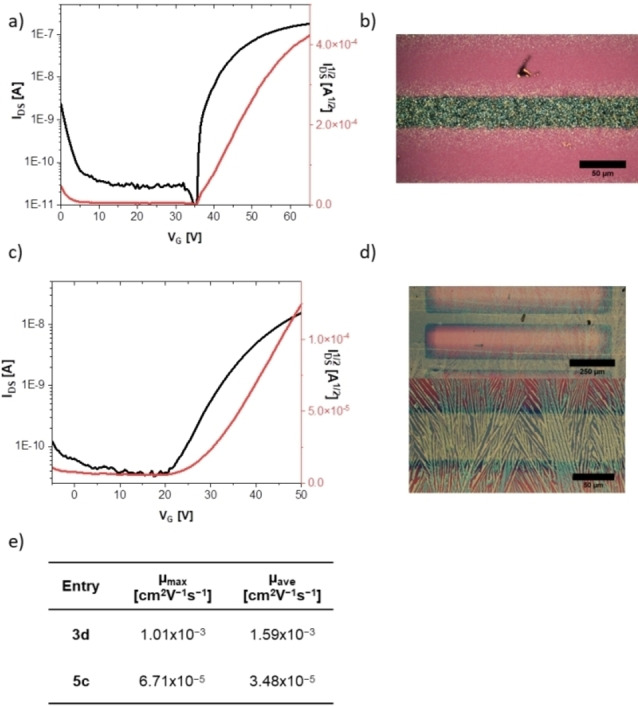
a) Transfer characteristics of **3 d**. b) Micrograph of a film of **3 d**. c) Transfer characteristics of **5 c**. d) Micrograph of a film of **5 c**. e) Charge transfer mobilities (electron) of **3 d** and **5 c**.

Unfortunately, the separation of the isomers **3 a** and **3 b** by preparative HPLC is only possible for small amounts impeding OFET construction/optimization. Also **3 c** and **4 b** could only be prepared in microscale synthesis. Instead, we attempted fabrication of bottom gate/top contact organic field‐effect transistors^[17]^ using **3 d** by drop‐casting from a mixture of THF/cyclohexane 1 : 1 (see Figure 3b for a micrograph of the thin film). We extracted an electron mobility with an average value of 1.59×10^−3^ cm^2^ V^−1^ s^−1^ (calcd: 1.2 cm^2^ V^−1^ s^−1^). We attribute the dramatic difference between experimental and calculated values to the microcrystallinity of the film exhibiting copious grain boundaries. We also assume a different thin‐film packing compared to that in the single crystals proven by powder XRD measurements (Figures S37 and S38) – the co‐crystallized and H‐bonded methanol (see above) is expected to change the packing.

Due to its low‐lying LUMO level, tetracyano compound **5 c** was characterized as an n‐type semiconductor in an OFET. Although the film (Figure 3d) consisted only of crystalline needles, which do not cover the channel completely, we measured an electron mobility of 3.5×10^−5^ cm^2^ V^−1^ s^−1^ (calcd: 1.7×10^−2^ cm^2^ V^−1^ s^−1^). Another aspect is the difference between the work function of the gold electrodes^[19]^ and the LUMO of the compound of approximately 1.3 eV. Nevertheless, the calculations support our results, as the electron mobility of **3 d** is two orders of magnitude higher compared to **5 c**. The cyclopentannulated tetrabromotetraazapentacene **4 c** only gave separated microcrystalline domains – a consequence of spinodal dewetting – although different solvent mixtures and processing techniques such as drop casting and dip coating were attempted.

**Figure 4:**
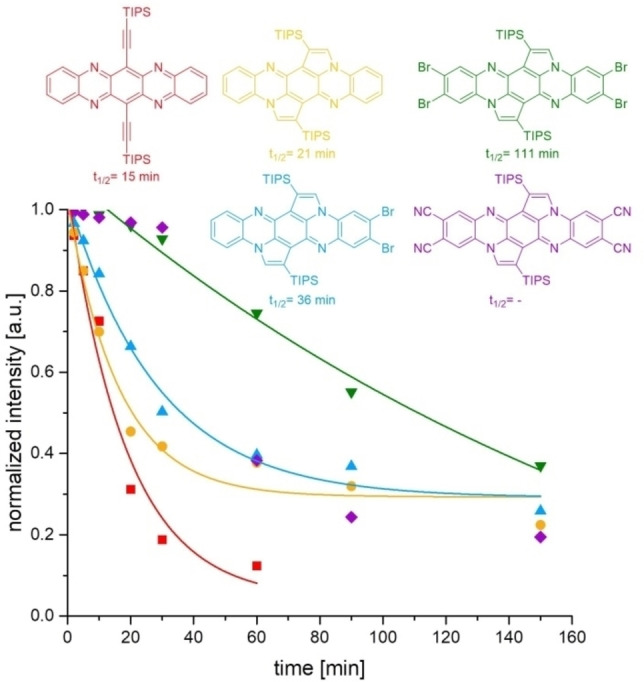
Time‐dependent decrease in the absorption bands of TIPS‐TAP (679 nm), **D** (468 nm), **3 d** (478 nm), **4 c** (486 nm) and **5 c** (499 nm) under irradiation with white and UV light (365 nm) in DCM under air. For clarity, intensity values were fitted with an exponential fit (except for **5 c**).

To proof the stability of the synthesized compounds, we recorded time‐dependent absorption spectra of DCM solutions (10^−5^ mol L^−1^) under irradiation with white and UV light (365 nm) under air (Figure 4). Due to the similar optoelectronic properties of the silyl isomers, we only measured the stabilities for the doubly shifted compounds **3 d**, **4 c** and **5 c** – as a reference **D** (doubly silyl‐shifted isomer) and TIPS‐TAP were used. A clear trend is noticeable for the stabilities, starting with TIPS‐TAP as the least stable compound under these conditions (*t*_1/2_=15 min). **D** shows a higher stability (*t*_1/2_=21 min) and the di‐ and tetrabromination of **3 d** and **4 c** increase the stability further to 36 and 111 min, respectively.^[18]^

## Conclusion

In summary, we have synthesized the brominated cyclopentannulated tetraazapentacenes **3 a**–**d**/**4 a**–**c** and demonstrated post‐functionalization by Stille cross‐coupling and Rosenmund‐von Braun reaction. Dibromo **3 d** and tetracyano **5 c** work as n‐type semiconductors in organic thin‐film transistors. The results agreed qualitatively with the outcome of quantum chemical studies. Compounds **3 d**, **4 c** and **5 c** show a higher stability than TIPS‐TAP. We expect further enhancement of these preliminary results through fine‐tuning of the molecules’ properties by potent postfunctionalization and π‐extensions of the core structures.

## Experimental Section

Deposition Numbers 2178793 (**3 a**), 2178794 (**3 b**), 2178795 (**3 c**), 2178796 (**3 d**), 2178797 (**4 b**) and 2178798 (**5 c**) contain the supplementary crystallographic data for this paper. These data are provided free of charge by the joint Cambridge Crystallographic Data Centre and Fachinformationszentrum Karlsruhe Access Structures service.

## Conflict of interest

The authors declare no conflict of interest.

1

## Supporting information

As a service to our authors and readers, this journal provides supporting information supplied by the authors. Such materials are peer reviewed and may be re‐organized for online delivery, but are not copy‐edited or typeset. Technical support issues arising from supporting information (other than missing files) should be addressed to the authors.

Supporting Information

## Data Availability

After acception of the manuscript, all raw data will be avavaible on HeiData, the data repository of the University of Heidelberg, under the following DOI: 10.11588/data/AUTKBW.

## References

[1a] U. H. F. Bunz , Chem. Eur. J. 2009, 15, 6780–6789; 19551789 10.1002/chem.200900990

[1b] Q. Miao , Synlett 2012, 23, 326–336;

[1c] Q. Miao , Adv. Mater. 2014, 26, 5541–5549; 24585514 10.1002/adma.201305497

[1d] C. Wang , H. Dong , W. Hu , Y. Liu , D. Zhu , Chem. Rev. 2012, 112, 2208–2267. 22111507 10.1021/cr100380z

[2a] U. H. F. Bunz , Acc. Chem. Res. 2015, 48, 1676–1686; 25970089 10.1021/acs.accounts.5b00118

[2b] C. Tönshoff , H. F. Bettinger , Chem. Eur. J. 2021, 27, 3193–3212. 33368683 10.1002/chem.202003112 PMC7898397

[3a] J. E. Anthony , J. S. Brooks , D. L. Eaton , S. R. Parkin , J. Am. Chem. Soc. 2001, 123, 9482–9483; 11562247 10.1021/ja0162459

[3b] B. Purushothaman , S. R. Parkin , J. E. Anthony , Org. Lett. 2010, 12, 2060–2063; 20373818 10.1021/ol100178s

[3c] Y. Fujiwara , R. Ozawa , D. Onuma , K. Suzuki , K. Yoza , K. Kobayashi , J. Org. Chem. 2013, 78, 2206–2212; 23323674 10.1021/jo302621k

[3d] R. Dorel , A. M. Echavarren , Eur. J. Org. Chem. 2017, 2017, 14–24; 10.1002/ejoc.201601129 PMC5484365 28747846

[3e] M. Müller , L. Ahrens , V. Brosius , J. Freudenberg , U. H. F. Bunz , J. Mater. Chem. C 2019, 7, 14011–14034;

[3f] A. Riaño , M. Carini , M. Melle-Franco , A. Mateo-Alonso , J. Am. Chem. Soc. 2020, 142, 20481–20488; 33213145 10.1021/jacs.0c10345

[3g] M. Nathusius , D. Sleeman , J. Pan , F. Rominger , J. Freudenberg , U. H. F. Bunz , K. Müllen , Chem. Eur. J. 2021, 27, 16606–16610; 34519387 10.1002/chem.202103285 PMC9293334

[3h] L. Ahrens , O. Tverskoy , S. Weigold , M. Ganschow , F. Rominger , J. Freudenberg , U. H. F. Bunz , Angew. Chem. Int. Ed. 2021, 60, 9270–9273; 10.1002/anie.202015348 PMC8247972 33259123

[3i] Z. Wang , P. Gu , G. Liu , H. Yao , Y. Wu , Y. Li , G. Rakesh , J. Zhu , H. Fu , Q. Zhang , Chem. Commun. 2017, 53, 7772–7775; 10.1039/c7cc03898d 28650017

[3j] Z. Zhang , Q. Zhang , Mater. Chem. Front. 2020, 4, 3419–3432;

[3k] Z. Zhang , Z. Wang , N. Aratani , X. Zhu , Q. Zhang , CCS 2022, 10.31635/ccschem.022.202202013.35057808

[4a] B. Kohl , F. Rominger , M. Mastalerz , Angew. Chem. Int. Ed. 2015, 54, 6051–6056; 10.1002/anie.201411972 25820770

[4b] B.-L. Hu , K. Zhang , C. An , D. Schollmeyer , W. Pisula , M. Baumgarten , Angew. Chem. Int. Ed. 2018, 57, 12375–12379; 10.1002/anie.201803230 30070417

[5] S. R. Bheemireddy , P. C. Ubaldo , P. W. Rose , A. D. Finke , J. Zhuang , L. Wang , K. N. Plunkett , Angew. Chem. Int. Ed. 2015, 54, 15762–15766; 10.1002/anie.201508650 26768696

[6] R. Heckershoff , S. Maier , T. Wurm , P. Biegger , K. Brödner , P. Krämer , M. T. Hoffmann , L. Eberle , J. Stein , F. Rominger , M. Rudolph , J. Freudenberg , A. Dreuw , A. S. K. Hashmi , U. H. F. Bunz , Chem. Eur. J. 2022, 28, e202104203. 35020239 10.1002/chem.202104203 PMC9303544

[7] For the use of gold catalysis for materials science, see:

[7a] C. M. Hendrich , K. Sekine , T. Koshikawa , K. Tanaka , A. S. K. Hashmi , Chem. Rev. 2021, 121, 9113–9163; 33315377 10.1021/acs.chemrev.0c00824

[7b] R. Heckershoff , T. Schnitzer , T. Diederich , L. Eberle , P. Krämer , F. Rominger , M. Rudolph , A. S. K. Hashmi , J. Am. Chem. Soc. 2022, 144, 8306–8316; 35471963 10.1021/jacs.2c02394

[7c] F. Stuck , M. C. Dietl , M. Meißner , F. Sebastian , M. Rudolph , F. Rominger , P. Krämer , A. S. K. Hashmi , Angew. Chem. Int. Ed. 2022, 61, e202114277; 10.1002/anie.202114277 PMC9299885 34755928

[7d] A. S. K. Hashmi , Chem. Rev. 2007, 107, 3180–3211. 17580975 10.1021/cr000436x

[8a] T. Okamoto , M. L. Senatore , M.-M. Ling , A. B. Mallik , M. L. Tang , Z. Bao , Adv. Mater. 2007, 19, 3381–3384;

[8b] J. Schwaben , N. Münster , M. Klues , T. Breuer , P. Hofmann , K. Harms , G. Witte , U. Koert , Chem. Eur. J. 2015, 21, 13758–13771; 26248605 10.1002/chem.201501399

[8c] J. Aragó , P. M. Viruela , E. Ortí , R. Malavé Osuna , V. Hernández , J. T. López Navarrete , C. R. Swartz , J. E. Anthony , Theor. Chem. Acc. 2011, 128, 521–530;

[8d] M. Müller , S. S. Beglaryan , S. Koser , S. Hahn , O. Tverskoy , F. Rominger , U. H. F. Bunz , Chem. Eur. J. 2017, 23, 7066–7073; 28295719 10.1002/chem.201700421

[8e] Y. Shu , Y.-F. Lim , Z. Li , B. Purushothaman , R. Hallani , J. E. Kim , S. R. Parkin , G. G. Malliaras , J. E. Anthony , Chem. Sci. 2011, 2, 363–368.

[9a] C. R. Swartz , S. R. Parkin , J. E. Bullock , J. E. Anthony , A. C. Mayer , G. G. Malliaras , Org. Lett. 2005, 7, 3163–3166; 16018611 10.1021/ol050872b

[9b] M.-Y. Kuo , H.-Y. Chen , I. Chao , Chem. Eur. J. 2007, 13, 4750–4758; 17373008 10.1002/chem.200601803

[9c] S. Chai , S.-H. Wen , J.-D. Huang , K.-L. Han , J. Comput. Chem. 2011, 32, 3218–3225; 21837726 10.1002/jcc.21904

[9d] A. Naibi Lakshminarayana , A. Ong , C. Chi , J. Mater. Chem. C 2018, 6, 3551–3563.

[10] Compound **1** consists of a mixture of tautomers, only one of which is shown for clarity.

[11] J. U. Engelhart , F. Paulus , M. Schaffroth , V. Vasilenko , O. Tverskoy , F. Rominger , U. H. F. Bunz , J. Org. Chem. 2016, 81, 1198–1205. 26765520 10.1021/acs.joc.5b02731

[12a] K. W. Rosenmund , E. Struck , Ber. Dtsch. Chem. Ges. A 1919, 52, 1749–1756;

[12b] G. P. Ellis , T. M. Romney-Alexander , Chem. Rev. 1987, 87, 779–794;

[12c] J. v. Braun , G. Manz , Justus Liebigs Ann. Chem. 1931, 488, 111–126.

[13] A. Naibi Lakshminarayana , J. Chang , J. Luo , B. Zheng , K.-W. Huang , C. Chi , Chem. Commun. 2015, 51, 3604–3607. 10.1039/c4cc09812a 25634022

[14] *Gaussian 16, Revision B.01*, M. J. Frisch, G. W. Trucks, H. B. Schlegel, G. E. Scuseria, M. A. Robb, J. R. Cheeseman, G. Scalmani, V. Barone, G. A. Petersson, H. Nakatsuji, X. Li, M. Caricato, A. V. Marenich, J. Bloino, B. G. Janesko, R. Gomperts, B. Mennucci, H. P. Hratchian, J. V. Ortiz, A. F. Izmaylov, J. L. Sonnenberg, D. Williams-Young, F. Ding, F. Lipparini, F. Egidi, J. Goings, B. Peng, A. Petrone, T. Henderson, D. Ranasinghe, V. G. Zakrzewski, J. Gao, N. Rega, G. Zheng, W. Liang, M. Hada, M. Ehara, K. Toyota, R. Fukuda, J. Hasegawa, M. Ishida, T. Nakajima, Y. Honda, O. Kitao, H. Nakai, T. Vreven, K. Throssell, J. A. Montgomery, Jr., J. E. Peralta, F. Ogliaro, M. J. Bearpark, J. J. Heyd, E. N. Brothers, K. N. Kudin, V. N. Staroverov, T. A. Keith, R. Kobayashi, J. Normand, K. Raghavachari, A. P. Rendell, J. C. Burant, S. S. Iyengar, J. Tomasi, M. Cossi, J. M. Millam, M. Klene, C. Adamo, R. Cammi, J. W. Ochterski, R. L. Martin, K. Morokuma, O. Farkas, J. B. Foresman, and D. J. Fox, Gaussian, Inc., Wallingford CT, **2016**.

[15] We were not able to obtain suitable crystals for single crystal analysis for **4 a** and **4 c**. Although these compounds are highly crystalline, the corresponding crystals were too small due to the poor solubility.

[16a] *ADF 2021.1*, SCM, Theoretical Chemistry, Vrije Universiteit, Amsterdam, The Netherlands, E. J. Baerends, T. Ziegler, A. J. Atkins, J. Autschbach, O. Baseggio, D. Bashford, A. Bérces, F. M. Bickelhaupt, C. Bo, P. M. Boerrigter, C. Cappelli, L. Cavallo, C. Daul, D. P. Chong, D. V. Chulhai, L. Deng, R. M. Dickson, J. M. Dieterich, F. Egidi, D. E. Ellis, M. van Faassen, L. Fan, T. H. Fischer, A. Förster, C. Fonseca Guerra, M. Franchini, A. Ghysels, A. Giammona, S. J. A. van Gisbergen, A. Goez, A. W. Götz, J. A. Groeneveld, O. V. Gritsenko, M. Grüning, S. Gusarov, F. E. Harris, P. van den Hoek, Z. Hu, C. R. Jacob, H. Jacobsen, L. Jensen, L. Joubert, J. W. Kaminski, G. van Kessel, C. König, F. Kootstra, A. Kovalenko, M. V. Krykunov, P. Lafiosca, E. van Lenthe, D. A. McCormack, M. Medves, A. Michalak, M. Mitoraj, S. M. Morton, J. Neugebauer, V. P. Nicu, L. Noodleman, V. P. Osinga, S. Patchkovskii, M. Pavanello, C. A. Peeples, P. H. T. Philipsen, D. Post, C. C. Pye, H. Ramanantoanina, P. Ramos, W. Ravenek, M. Reimann, J. I. Rodríguez, P. Ros, R. Rüger, P. R. T. Schipper, D. Schlüns, H. van Schoot, G. Schreckenbach, J. S. Seldenthuis, M. Seth, J. G. Snijders, M. Solà, M. Stener, M. Swart, D. Swerhone, V. Tognetti, G. te Velde, P. Vernooijs, L. Versluis, L. Visscher, O. Visser, F. Wang, T. A. Wesolowski, E. M. van Wezenbeek, G. Wiesenekker, S. K. Wolff, T. K. Woo, A. L. Yakovlev;

[16b] A. Moliterni , D. Altamura , R. Lassandro , V. Olieric , G. Ferri , F. Cardarelli , A. Camposeo , D. Pisignano , J. E. Anthony , C. Giannini , Acta Crystallogr. Sect. B 2020, 76, 427–435; 10.1107/S2052520620004424 32831261

[16c] G. te Velde , F. M. Bickelhaupt , E. J. Baerends , C. Fonseca Guerra , S. J. A. van Gisbergen , J. G. Snijders , T. Ziegler , J. Comput. Chem. 2001, 22, 931–967.

[17a] D. Liu , Z. He , Y. Su , Y. Diao , S. C. B. Mannsfeld , Z. Bao , J. Xu , Q. Miao , Adv. Mater. 2014, 26, 7190–7196; 25205623 10.1002/adma.201402822

[17b] S. Maier , N. Hippchen , F. Rominger , J. Freudenberg , U. H. F. Bunz , Chem. Eur. J. 2021, 27, 16320–16324. 34612544 10.1002/chem.202103193 PMC9297893

[18] We were not able to determine a half-life of the tetracyano compound **5 c** due to an unusual accelerated degradation after different time intervals (non-consistent in different experiments). Therefore the time-dependent decrease of the absorption band at 499 nm is not exponential and therefore a fit was not applied.

[19] D. E. Eastman , Phys. Rev. B 1970, 2, 1–2, 10.1103/PhysRevB.2.1.

