# Supplementary material for: Substituted Cyclopentannulated Tetraazapentacenes
Source: Chemistry. 2022 Sep 19;28(64):e202201842. doi: 10.1002/chem.202201842 (PMC9826220; doi:10.1002/chem.202201842)
Supplement: Supplementary file 1 — Supporting Information [file CHEM-28-0-s001.pdf]

Chemistry–A European Journal

Supporting Information

Substituted Cyclopentannulated Tetraazapentacenes

Steffen Maier, Robin Heckershoff, Nikolai Hippchen, Kerstin Brödner, Frank Rominger, Jan Freudenberg,* A. Stephen K. Hashmi,* and Uwe H. F. Bunz*

Table of Contents

1 Experimental Procedures 2
1.1 General Information 2
1.2 Synthesis of Compounds 3
2 NMR Spectra 11
3 UV-Vis Spectra 19
4 IR Spectra 23
5 Crystallographic Data 27
6 Computational Details 35
6.1 Calculations of the Optoelectronic Properties 35
6.1.1 FMO Calculations 35
6.1.2 Calculation of Theoretical Electron Mobilities 42
6.1.3 $NICS_{zz}$ XY-Scans 47
6.1.4 Coordinates of the Optimized Geometries 48
7 Device Fabrication and Measurement 65
8 Stability Measurements 66
9 References 67

1 Experimental Procedures

1.1 General Information

Chemicals were bought from commercial suppliers (ABCR, Acros, Alfa Aesar, Carbolution, Chempur, Fluka, Merck, Sigma Aldrich and TCI) and used as delivered. Anhydrous solvents were dispensed from a solvent purification system MB SPS-800. Deuterated solvents were bought from Euriso Top and Sigma Aldrich.
Melting points (mp) were measured in open glass capillaries on a Stuart SMP10 melting point apparatus and are uncorrected.
R_f-values were determined by analytical thin layer chromatography (TLC) on aluminum sheets coated with silica gel produced by Macherey-Nagel (ALUGRAM® Xtra SIL G/25 UV_{254}). Detection was accomplished using UV-light (254 and 365 nm) or a TLC staining solution (vanillin).
Nuclear magnetic resonance (NMR) spectra were recorded at the chemistry department of Heidelberg University under the supervision of Dr. J. Graf on the following spectrometers: Bruker Avance III 300 (300 MHz), Bruker Avance DRX 300 (300 MHz), Bruker Fourier 300 (300 MHz), Bruker Avance III 400 (400 MHz), Bruker Avance III 500 (500 MHz) and Bruker Avance III 600 (600 MHz). $CDCl_3$ was filtered through a plug of aluminum oxide (ALOX) prior to use to remove acid impurities. Chemical shifts (δ) are given in ppm and coupling constants *J* in Hz. Spectra were referenced to residual solvent protons according to Fulmer *et al*.[1] The following abbreviations were used to describe the observed multiplicities: for ^{1}H NMR spectra: s = singlet, d = doublet, sept = septet, m = multiplet br = broad signal.
High-resolution mass spectra (HR-MS) were recorded at the chemistry department of Heidelberg University under the supervision of Dr. J. Gross on the following spectrometers: JEOL AccuTOF GCx (EI), Bruker ApexQe hybrid 9.4 T FT-ICR (ESI, MALDI, DART), Finnigan LCQ (ESI) and Bruker AutoFlex Speed (MALDI). For MALDI, *trans*-2-[3-(4-*tert*-butylphenyl)-2-methyl-2-propenylidene]-malononitrile (DCTB) was used as matrix.
Infrared spectra were recorded from a neat powder on a FT/IR spectrometer (Bruker LUMOS or Jasco FT/IR-4100) with a Germanium ATR-crystal. For the most significant bands the wave numbers are given.
UV-Vis spectra were recorded on a Jasco UV-VIS V-670. Fluorescence spectra were recorded on a Jasco FP6500. Quantumyields (QY) were recorded on a Jasco FP-8600 fluorescence spectrometer equipped with a ILF-835 100 mm dia. integrating sphere.
X-ray crystallography was carried out at the chemistry department of Heidelberg University under the supervision of Dr. F. Rominger on the following instruments: Bruker Smart APEX II Quazar (with Mo-microsource) and Stoe Stadivari (with Co-microsource and Pilatus detector).
HPLC was carried out on an Agilent 1100 series HPLC system (column: Merck LiChrosorb® Si 60 (5 µm); solvent: hexane to hexane:DCM = 1:1, linear gradient; flow rate: 1 mL/min; UV detection at 254 nm). For flash column chromatography silica gel (Sigma-Aldrich, pore size 60 Å, 70–230 mesh, 63–200 µm) was used as stationary phase. As eluents different mixtures of petroleum ether (PE), ethyl acetate (EA), dichloromethane (DCM) or MeOH were used.
All reactions were performed under air, if not otherwise specified. For handling of air and moisture sensitive reagents, standard Schlenk techniques with flame-dried glassware under an argon or nitrogen atmosphere were used.
1,4-Bis((triisopropylsilyl)ethynyl)phenazine-2,3-diamine (**S1**),[2] 4,5-dibromobenzene-1,2-diol (**S2**)[3] and 2,3,9,10-tetrabromo-6,13-bis((triisopropylsilyl)ethynyl)quinoxalino[2,3-*b*]phenazine (**S5**)[4] were synthesized according to literature procedures.

1.2 Synthesis of Compounds

2,3-Dibromo-6,13-bis((triisopropylsilyl)ethynyl)-5,14-dihydroquinoxalino[2,3-*b*]phenazine / 9,10-dibromo-6,13-bis((triisopropylsilyl)ethynyl)-5,14-dihydroquinoxalino[2,3-*b*]phenazine, mixture of tautomers (1)

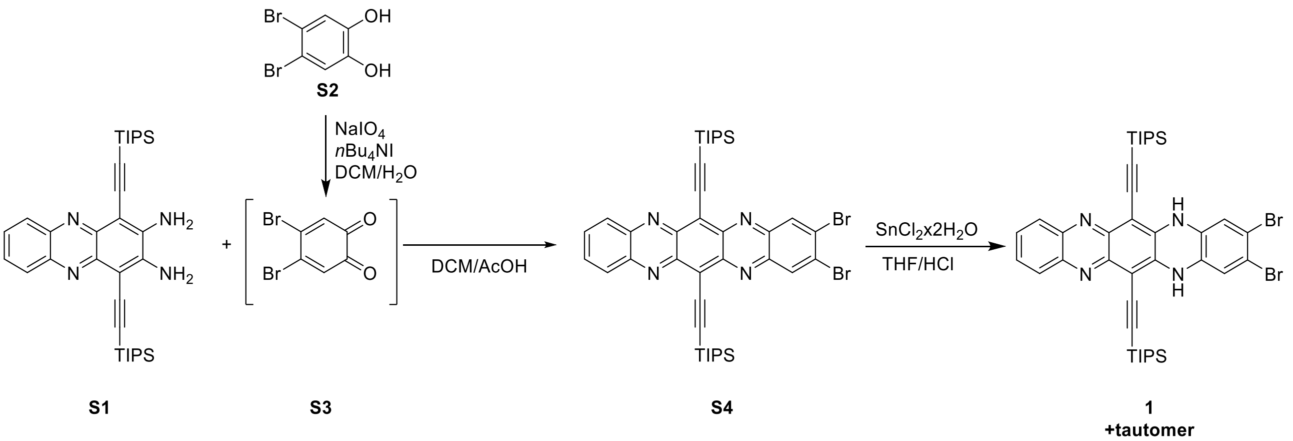

Diol **S2** (5.27 g, 19.7 mmol, 1.00 eq.) was dissolved in 100 mL of DCM. Subsequently, sodium metaperiodate (5.05 g, 23.6 mmol, 1.20 eq.) and NBu_4I (243 mg, 787 µmol, 4 mol%.) were added. After addition of 100 mL of deionized water, the reaction mixture was stirred for 1 h at room temperature. The reaction mixture was extracted with DCM and the combined organic layers were dried over $MgSO_4$. After removal of the solvent under reduced pressure, crude quinone **S3** was dissolved in a mixture of 40 mL DCM and 40 mL acetic acid. After addition of diamine **S1** (5.00 g, 8.76 mmol, 0.44 eq) the reaction mixture was stirred at room temperature overnight. The end of the reaction was monitored by TLC. After completion, an aqueous solution of $NaHCO_3$ was added to neutralize the solution. The aqueous phase was extracted with DCM, the combined organic layers were dried over $MgSO_4$ and the solvent was removed under reduced pressure. The crude product was purified by flash column chromatography (silica gel, PE to PE:DCM = 8:2 to 7:3) to yield dibromotetraazapentacene **S4** as dark green solid (4.72 g, 5.91 mmol, 67%, Rf: 0.40 (silica gel, PE:DCM = 7:3)), which was used without further characterization. **S4** (4.72 g, 5.89 mmol, 1.00 eq) was dissolved in 200 mL of THF. A solution of tin(II)chloride dihydrate (13.3 g, 58.9 mmol, 10.0 eq.) in 200 mL of 6 M HCl was added dropwise. The reaction mixture was stirred at room temperature for 4 h and extracted with DCM. The combined organic layers were washed three times with a 2 M NaOH solution and dried over $MgSO_4$. After removal of the solvent the product was yielded as a mixture of tautomers (ratio determined by ^{1}H NMR: 4:1) as a violet-golden solid (3.50 g, 4.37 mmol, 74%).

Tautomer A:

^{1}H NMR (600 MHz, $CDCl_3$, 295 K): δ = 8.16 (s, 2H), 7.20 (s, 2H), 6.82–6.79 (m, 2H), 6.52–6.49 (m, 2H), 1.25–1.24 (m, 42H) ppm.

^{13}C{^{1}H} NMR (151 MHz, $CDCl_3$, 295 K): δ = 144.6, 141.4, 140.4, 132.8, 128.0, 124.1, 123.9, 114.2, 105.1, 99.1, 96.7, 19.0, 11.5 ppm.

Tautomer B:

^{1}H NMR (600 MHz, $CDCl_3$, 295 K): δ = 7.93–7.92 (m, 2H), 7.58–7.56 (m, 2H), 6.98 (s, 2H), 6.65 (s, 2H), 1.25–1.24 (m, 42H) ppm.

^{13}C{^{1}H} NMR (151 MHz, $CDCl_3$, 295 K): δ = 143.3, 142.4, 138.2, 129.2, 128.9, 128.8, 117.8, 117.1, 105.7, 99.1, 98.6, 19.0, 11.5 ppm.

Tautomer A+B:

Mp: >300 °C.

R*f*: 0.35 (silica gel, PE:DCM = 7:3).

ATR-IR: $\tilde{\nu}$ [cm^{-1}] = 3372, 2939, 2888, 2862, 2161, 2153, 2144, 2139, 2122, 2097, 1611, 1599, 1574, 1523, 1494, 1453, 1415, 1398, 1387, 1366, 1328, 1308, 1287, 1269, 1253, 1236, 1224, 1202, 1154, 1125, 1109, 1080, 1038, 1025, 1014, 993, 950, 918, 907, 880, 870, 842, 817, 809, 751, 730, 704, 669, 659, 645, 623, 587, 581, 552, 547, 534, 517, 503, 492, 485, 478, 455, 443, 430, 425, 420, 418, 410, 404.

HR-MS (MALDI pos.): m/z: $[M]^+$: calcd. for $[C_{40}H_{50}N_4Si_2{}^{79}Br_2]^+$: 800.1935; found: 800.1940.

2,3,9,10-Tetrabromo-6,13-bis((triisopropylsilyl)ethynyl)-5,14-dihydroquinoxalino[2,3-*b*]phenazine (2)

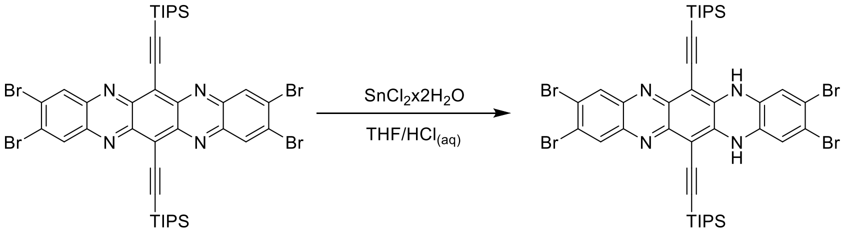

S5 **2**

S5 (1.20 g, 1.25 mmol, 1.00 eq.) was dissolved in 30 mL of THF. A solution of tin(II)chloride dihydrate (2.82 g, 12.5 mmol, 10.0 eq.) in 30 mL hydrochlorid acid (6 M) was added dropwise. The solution changed the color from green to violet/blue. The mixture was stirred for 2 h at room temperature. DCM was added and the mixture was extracted with DCM. The combined organic layers were washed three times with a 2 M sodium hydroxide solution and were afterwards dried over $MgSO_4$. After removing of the solvent, the product was yielded as a violet solid (1.12 g, 1.17 mmol, 93%).

^{1}H NMR (300 MHz, $CDCl_3$, 298 K): δ = 8.19 (s, 2H), 7.08 (s, 2H), 6.68 (s, 2H), 1.25 (s, 42H) ppm.

The analytical data is in agreement with the literature.[5]

4,5-Dibromo-2,9-bis(triisopropylsilyl)-2a,7,9a,14-tetraazadicyclopenta[*fg*,*qr*]pentacene (3a), 4,5-dibromo-2,8-bis(triisopropylsilyl)-2a,7,9a,14-tetraazadicyclopenta[*fg*,*qr*]pentacene (3b), 4,5-dibromo-1,9-bis(triisopropylsilyl)-2a,7,9a,14-tetraazadicyclopenta[*fg*,*qr*]pentacene (3c)

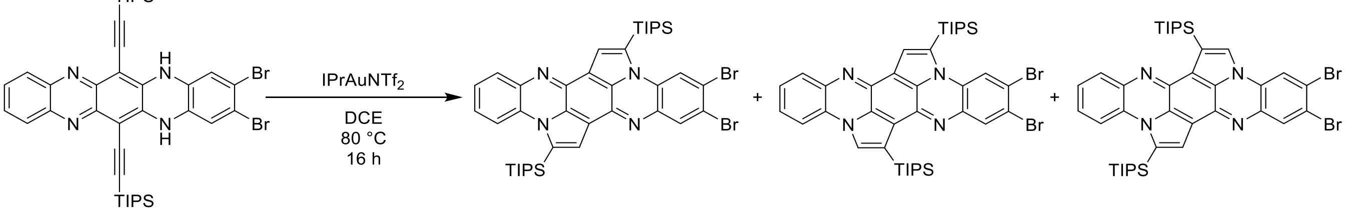

1 **3a** **3b** **3c**
+tautomer

1 (100 mg, 125 µmol, 1.00 eq.) and $IPrAuNTf_2$ (10.8 mg, 12.5 µmol, 10 mol%) were dissolved in DCE (15 mL). The reaction mixture was stirred at 80 °C for 2 d. After cooling to room temperature, the solvent was removed under reduced pressure and the crude product was purified by flash column chromatography (silica gel, PE:EA = 40:1) to yield a mixture of the two isomers **3a** and **3b** (ratio ~ 7:3) as a yellow solid (86.6 mg, 107 µmol, 87%) as well as pure **3c** (6.2 mg, 7.72 µmol, 6%). To obtain the

pure compounds **3a** and **3b** a part of the mixture was separated by preparative HPLC (ReproSil-Pur 120 Si, PE:EA = 20:1) to yield pure **3a** and **3b** as yellow solids.

3a:

Mp: >300 °C

R$_f$: 0.20 (silica gel, PE:EA = 20:1).

^{1}H NMR (600 MHz, $CDCl_3$, 298 K): δ = 8.33 (s, 1H), 8.28 (s, 1H), 8.09–8.07 (m, 1H), 8.05–8.04 (m, 1H), 7.86 (s, 1H), 7.83 (s, 1H), 7.53–7.48 (m, 2H), 1.74 (sept, $^3J_{HH}$ = 7.6 Hz, 6H), 1.25–1.21 (m, 36H) ppm.

^{13}C{^{1}H} NMR (151 MHz, $CDCl_3$, 298 K): δ = 147.7, 146.2, 140.5, 140.2, 133.7, 132.0, 131.9, 130.4, 130.4, 130.1, 129.7, 129.5, 126.6, 126.3, 123.4, 123.1, 121.2, 121.1, 120.7, 117.3, 116.7, 116.1, 19.3, 19.3, 13.9 ppm.

ATR-IR: $\tilde{\nu}$ [cm^{-1}] = 2947, 2888, 2865, 1606, 1557, 1543, 1521, 1460, 1435, 1423, 1409, 1391, 1376, 1367, 1277, 1188, 1132, 1116, 1106, 1077, 1046, 1019, 1006, 990, 968, 906, 878, 846, 758, 746, 732, 706, 688, 676, 669, 661, 651, 634, 617, 610, 606, 593, 572, 555, 547, 542, 519, 499, 487, 482, 475, 468, 464, 459, 456, 452, 444, 418.

HR-MS (DART pos.): m/z: $[M+H]^+$: calcd. for $[C_{40}H_{51}N_4Si_2{}^{79}Br_2]^+$: 801.2014; found: 801.2007.

3b:

Mp: >300 °C

R$_f$: 0.26 (silica gel, PE:EA = 20:1).

^{1}H NMR (600 MHz, $CDCl_3$, 298 K): δ = 8.34 (s, 1H), 8.09 (s, 1H), 8.05–8.03 (m, 1H), 7.92–7.85 (m, 2H), 7.85 (s, 1H), 7.53–7.49 (m, 2H), 1.93 (sept, $^3J_{HH}$ = 7.5 Hz, 3H), 1.74 (sept, $^3J_{HH}$ = 7.5 Hz, 3H), 1.26–1.21 (m, 36H) ppm.

^{13}C{^{1}H} NMR (151 MHz, $CDCl_3$, 298 K): δ = 148.6, 146.0, 140.5, 139.2, 133.5, 132.2, 129.9, 129.5, 129.2, 127.5, 127.0, 126.5, 123.1, 123.0, 121.1, 121.0, 120.9, 120.7, 118.8, 116.7, 114.4, 19.4, 19.4, 14.0, 12.5 ppm.

ATR-IR: $\tilde{\nu}$ [cm^{-1}] = 3154, 3138, 3131, 3115, 2954, 2941, 2888, 2864, 2754, 2741, 2728, 2715, 2359, 1581, 1553, 1520, 1500, 1488, 1479, 1456, 1429, 1406, 1382, 1363, 1340, 1289, 1198, 1164, 1155, 1123, 1111, 1097, 1077, 1073, 1052, 1019, 999, 987, 954, 920, 880, 858, 850, 845, 841, 819, 770, 748, 734, 706, 684, 666, 655, 650, 646, 638, 632, 621, 611, 599, 593, 580, 572, 563, 552, 547, 543, 538, 530, 502, 493, 489, 483, 475, 468, 463, 458, 444, 441, 433, 426, 421, 416, 410, 406, 403.

HR-MS (DART pos.): m/z: $[M+H]^+$: calcd. for $[C_{40}H_{51}N_4Si_2{}^{79}Br_2]^+$: 801.2014; found: 801.2046.

3c:

Mp: >300 °C

R$_f$: 0.50 (silica gel, PE:EA = 20:1).

^{1}H NMR (400 MHz, $CDCl_3$, 298 K): δ = 8.25 (br, 1H), 8.09–7.99 (m, 3H), 7.81–7.79 (m, 2H), 7.53–7.49 (m, 2H), 2.00 (sept, $^3J_{HH}$ = 7.6 Hz, 3H), 1.74 (sept, $^3J_{HH}$ = 7.5 Hz, 3H), 1.26–1.21 (m, 36H) ppm.

$^{13}C\{^1H\}$ NMR (126 MHz, $CDCl_3$, 298 K): δ = 147.5, 147.0, 140.2, 139.6, 133.6, 132.3, 130.5, 129.7, 129.4, 129.2, 127.0, 126.4, 126.2, 122.8, 122.8, 122.3, 121.8, 121.4, 119.3, 118.8, 116.6, 115.5, 19.4, 19.4, 14.0, 12.5 ppm.

ATR-IR: $\tilde{\nu}$ [cm^{-1}] = 2922, 2862, 1726, 1559, 1510, 1458, 1429, 1378, 1277, 1246, 1191, 1176, 1129, 1097, 1052, 1019, 996, 918, 880, 818, 801, 757, 744, 681, 664, 649, 620, 608, 582, 506, 496, 402.

HR-MS (MALDI pos.): m/z: $[M]^+$: calcd. for $[C_{40}H_{50}N_4Si_2{}^{79}Br_2]^+$: 800.1935; found: 800.1948.

11,12-Dibromo-1,8-bis(triisopropylsilyl)-2a,7,9a,14-tetraazadicyclopenta[*fg*,*qr*]pentacene (3d)

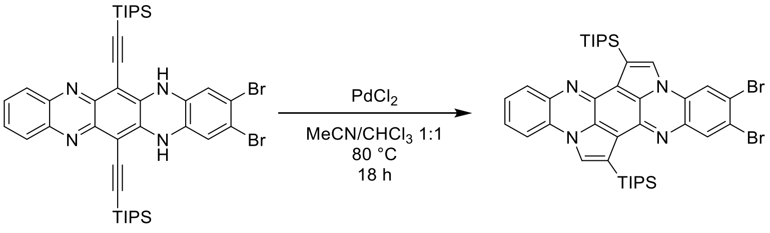

1
+ isomer

3d

Micoscale synthesis:

1 (120 mg, 150 µmol, 1.00 eq.) was dissolved in 5 mL of a 1:1 mixture of chloroform and acetonitrile. Palladium dichloride (5.73 mg, 15.0 µmol, 20 mol%) was added and the mixture was stirred for 18 h at 80 °C. The crude product was purified by flash column chromatography (silica gel, PE to PE:DCM = 9:1) to yield the product as a yellow solid (97.2 mg, 122 µmol, 81%).

Gramscale synthesis:

1 (1.33 g, 1.66 mmol, 1.00 eq.) was dissolved in 240 mL of a 1:1 mixture of chloroform and acetonitrile. Palladium dichloride (58.8 mg, 331 µmol, 20 mol%) was added and the mixture was stirred for 18 h at 80 °C. The crude product was suspended in 50 mL DCM at 40 °C. Afterwards, a 1:1 mixture of acetone and PE (300 mL) was added and the precipitated orange solid was filtered off to yield the product (906 mg, 1.13 mmol, 68%).

Mp: >300 °C

R_f: 0.70 (silica gel, PE:EA = 20:1).

^1H NMR (600 MHz, $CDCl_3$, 298 K): δ = 8.08–8.06 (m, 2H) 7.94–7.93 (m, 1H), 7.90–7.86 (m, 2H), 7.75 (s, 1H), 2.01–1.96 (m, 3H), 1.95–1.90 (m, 3H), 1.21–1.20 (m, 36H) ppm.

$^{13}C\{^1H\}$ NMR (151 MHz, $CDCl_3$, 298 K) δ = 148.4, 146.7, 139.6, 139.3, 133.4, 130.0, 130.0, 129.6, 127.3, 126.8, 126.8, 126.3, 122.7, 122.6, 121.8, 121.6, 121.2, 120.3, 118.7, 118.5, 118.0, 114.2, 19.3, 19.3, 12.4, 12.4 ppm.

ATR-IR: $\tilde{\nu}$ [cm^{-1}] = 2956, 2953, 2939, 2926, 2923, 2899, 2896, 2886, 2884, 2860, 1558, 1555, 1508, 1507, 1496, 1494, 1486, 1480, 1477, 1473, 1472, 1465, 1464, 1458, 1456, 1431, 1382, 1364, 1360, 1285, 1255, 1195, 1186, 1105, 1071, 1066, 1053, 1020, 1008, 996, 994, 992, 919, 880, 878, 865, 845, 824, 786, 771, 763, 731, 697, 682, 679, 678, 672, 670, 668, 666, 663, 659, 655, 652, 645, 643, 638, 623, 620, 618, 611, 607, 605, 600, 593, 591, 588, 581, 575, 569, 564, 559, 556, 553, 551, 549, 548, 540, 537, 511, 507, 503, 499, 497, 494, 492, 491, 490, 488, 485, 480, 477, 474, 472, 471, 468, 465, 463, 461, 458, 454, 451, 450, 448, 443, 442, 440, 438, 437, 435, 433, 431, 429, 426, 424, 423, 421, 418, 416, 413, 411, 410, 407, 403.

HR-MS (MALDI pos.): m/z: $[M]^+$: calcd. for $[C_{40}H_{50}N_4Si_2{}^{79}Br_2]^+$: 800.1935; found: 800.1906.

4,5,11,12-Tetrabromo-2,9-bis(triisopropylsilyl)-2a,7,9a,14-tetraazadicyclopenta[*fg*,*qr*]pentacene (4a), 4,5,11,12-tetrabromo-1,9-bis(triisopropylsilyl)-2a,7,9a,14-tetraazadicyclopenta[*fg*,*qr*]pentacene (4b)

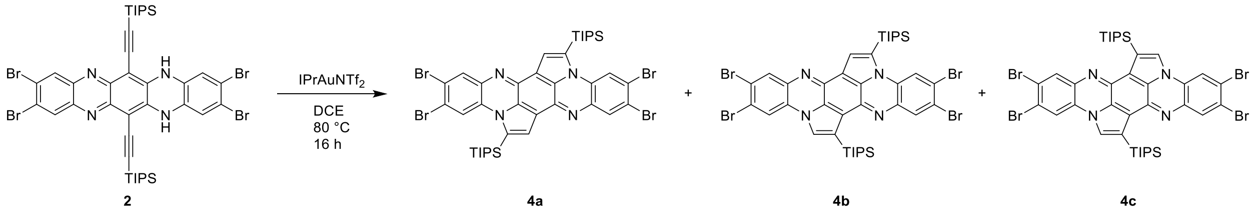

2 (150 mg, 156 µmol, 1.00 eq.) and $IPrAuNTf_2$ (13.5 mg, 15.6 µmol, 10 mol%) were dissolved in DCE (30 mL). The reaction mixture was stirred at 80 °C for 2 d. After cooling to room temperature, the solvent was removed under reduced pressure and the crude product was purified by flash column chromatography (silica gel, PE:EA = 200:1 to 1:1) to yield **4a** as a yellow solid (48.2 mg, 50.2 µmol, 32%), **4b** as a yellow solid (37.4 mg, 38.9 µmol, 25%) and **4c** as a yellow solid (4.50 mg, 4.68 µmol, 3%).

4a:

Mp: >300 °C

R*f*: 0.67 (silica gel, PE:EA = 20:1).

^{1}H NMR (300 MHz, $CDCl_3$, 298 K): δ = 8.35 (s, 2H), 8.30 (s, 2H), 7.82 (s, 2H), 1.78–1.68 (sept, $^3J_{HH}$ = 7.4 Hz, 6H), 1.25–1.23 (m, 36H) ppm.

^{13}C{^{1}H} NMR: Measurement was not possible due to the low solubility of the compound.

ATR-IR: $\tilde{\nu}$ [cm^{-1}] = 2952, 2921, 2852, 2356, 1737, 1594, 1554, 1513, 1457, 1431, 1410, 1391, 1377, 1363, 1280, 1260, 1252, 1188, 1170, 1162, 1115, 1099, 1075, 1051, 1020, 960, 919, 896, 879, 844, 839, 736, 677, 664, 593, 500, 454.

HR-MS (MALDI pos.): m/z: $[M]^+$: calcd. for $[C_{40}H_{48}N_4Si_2{}^{79}Br_4]^+$: 956.0146; found: 956.0152.

4b:

Mp: >300 °C

R*f*: 0.60 (silica gel, PE:EA = 20:1).

^{1}H NMR (600 MHz, $CDCl_3$, 298 K): δ = 8.36 (s, 1H), 8.28 (s, 1H), 8.12-8.11 (m, 2H), 7.80-7.79 (m, 2H), 1.93 (sept, $^3J_{HH}$ = 7.52 Hz, 3H), 1.73 (sept, $^3J_{HH}$ = 7.51 Hz, 3H), 1.25–1.24 (m, 18H), 1.21–1.20 (m, 18H) ppm.

^{13}C{^{1}H} NMR (101 MHz, $CDCl_3$, 298 K): δ = 148.2, 147.0, 140.3, 139.5, 133.9, 133.7, 132.1, 130.5, 129.3, 129.1, 126.8, 123.2, 123.1, 122.5, 121.7, 121.7, 121.4, 120.8, 119.9, 118.9, 116.5, 19.4, 14.0, 12.4 ppm.

ATR-IR: $\tilde{\nu}$ [cm^{-1}] = 2950, 2942, 2924, 2894, 2884, 2865, 1736, 1716, 1596, 1548, 1518, 1465, 1458, 1441, 1420, 1410, 1391, 1384, 1358, 1279, 1261, 1184, 1162, 1121, 1105, 1094, 1078, 1062, 1045, 1018, 995, 973, 922, 876, 847, 836, 799, 768, 752, 736, 700, 668, 651, 627, 613, 602, 579, 569, 565, 561, 548, 540, 514, 501, 492, 486, 479, 475, 472, 468, 458, 454, 451, 442, 438, 433, 430, 427, 419, 414, 409, 404.

HR-MS (DART pos.): m/z: [M+H]$^+$: calcd. for [$C_{40}H_{49}N_4Si_2{}^{79}Br_2{}^{81}Br_2$]$^+$: 961.0183; found: 961.0145.

4,5,11,12-Tetrabromo-1,8-bis(triisopropylsilyl)-2a,7,9a,14-tetraazadicyclopenta[*fg*,*qr*]pentacene (4c)

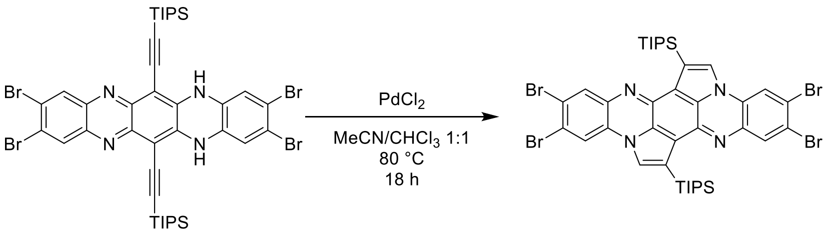

2 **4c**

Micoscale synthesis:

2 (20.0 mg, 20.8 µmol, 1.00 eq.) was dissolved in 2 mL of a 1:1 mixture of chloroform and acetonitrile. Palladium dichloride (738 µg, 4.16 µmol, 20 mol%) was added and the mixture was stirred for 18 h at 80 °C. The crude product was purified by flash column chromatography (silica gel, PE to PE:DCM = 9:1) to yield the product as a yellow solid (15.0 mg, 15.6 µmol, 75%).

Gramscale synthesis:

2 (2.50 g, 1.66 mmol, 1.00 eq.) was dissolved in 500 mL of a 1:1 mixture of chloroform and acetonitrile. Palladium dichloride (46.1 mg, 260 µmol, 20 mol%) was added and the mixture was stirred for 18 h at 80 °C. The crude product was suspended in 50 mL DCM at 40 °C. Afterwards, a 1:1 mixture of acetone and PE (500 mL) was added and the precipitated dark orange solid was filtered off. The procedure was repeated using THF (50 mL) and a mixture of DCM, acetone and PE for precipitation (equal amount, combined 500 mL). The corresponding brown solid was filtered off and dried under reduced pressure to yield the product (1.61 g, 1.67 mmol, 64%) as a dark orange solid.

Mp: >300 °C

R*f*: 0.95 (silica gel, PE:EA = 20:1).

^{1}H NMR (300 MHz, $CDCl_3$, 298 K): δ = 8.11–8.09 (m, 4H), 7.77 (s, 2H), 1.98–1.87 (m, 6H), 1.21–1.18 (m, 36H) ppm.

^{13}C{^{1}H} NMR: Measurement was not possible due to the low solubility of the compound.

ATR-IR: $\tilde{\nu}$ [cm^{-1}] = 2950, 2947, 2945, 2941, 2939, 2922, 2859, 1550, 1544, 1487, 1476, 1473, 1471, 1464, 1460, 1456, 1451, 1448, 1436, 1427, 1419, 1405, 1397, 1387, 1368, 1286, 1194, 1174, 1106, 1055, 1018, 995, 987, 916, 876, 839, 794, 768, 701, 699, 693, 679, 668, 665, 656, 654, 645, 643, 634, 626, 623, 620, 614, 613, 606, 595, 589, 587, 586, 584, 569, 567, 566, 562, 559, 550, 547, 546, 510, 508, 504, 502, 499, 495, 492, 488, 486, 484, 481, 479, 475, 471, 470, 467, 465, 463, 461, 459, 458, 457, 453, 451, 449, 447, 445, 443, 442, 440, 437, 435, 432, 429, 426, 424, 421, 419, 417, 415, 413, 409, 407, 405.

HR-MS (MALDI pos.): m/z: [M+H]$^+$: calcd. for [$C_{40}H_{47}N_4Si_2{}^{79}Br_2{}^{81}Br_2$]$^+$: 959.0026; found: 959.0020.

1,8-Bis(triisopropylsilyl)-2a,7,9a,14-tetraazadicyclopenta[*fg*,*qr*]pentacene-4,5,11,12-tetracarbonitrile (5c)

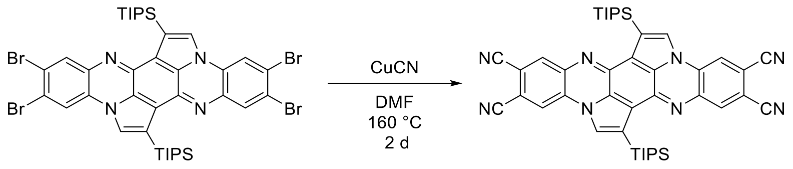

4c **5c**

A mixture of **4c** (82.0 mg, 85.4 µmol, 1 eq.) and CuCN (122 mg, 1.37 mmol, 16 eq) in DMF (10 mL) was stirred at 160 °C for 2 d. The solvent was removed under reduced pressure and water (20 mL) was added to the residue. The mixture was extracted with DCM, the combined organic layers were washed with water and brine and dried over Na_2SO_4. The solvent was removed under reduced pressure and the residue was purified by flash column chromatography (aluminum oxide, PE:DCM = 3:2 to 1:2). After further purification by recrystallization from DCM/MeOH and DCM/pentane, the product was obtained as a red-orange solid (46.7 mg, 62.7 mmol, 73%).

Mp: >300 °C

R*f*: 0.60 (silica gel, PE:EA = 5:1).

^{1}H NMR (301 MHz, $CDCl_3$, 298 K): δ = 8.28 (s, 2H), 8.26 (s, 2H), 7.93 (s, 2H), 1.91 (sept, $^3J_{HH}$ = 7.3 Hz, 6H), 1.20 (d, $^3J_{HH}$ = 7.5 Hz, 36H) ppm.

^{13}C{^{1}H} NMR (151 MHz, $CDCl_3$, 323 K): δ = 149.8, 141.7, 135.4, 130.3, 129.3, 124.3, 122.8, 122.0, 120.3, 115.2, 115.2, 113.1, 112.8, 19.3, 12.5 ppm.

ATR-IR: $\tilde{\nu}$ [cm^{-1}] = 2943, 2862, 2230, 1792, 1741, 1607, 1542, 1494, 1481, 1462, 1439, 1415, 1385, 1358, 1310, 1284, 1247, 1206, 1181, 1120, 1093, 1022, 996, 919, 897, 882, 872, 807, 777, 753, 699, 664, 648, 612.

HR-MS (MALDI neg.): *m/z*: [M]$^-$: calcd. for [$C_{44}H_{48}N_8Si_2$]: 744.3546; found: 744.3557.

1,8-Bis(triisopropylsilyl)-4,5,11,12-tetrakis((trimethylsilyl)ethynyl)-2a,7,9a,14-tetraazadicyclopenta[*fg*,*qr*]pentacene (6c)

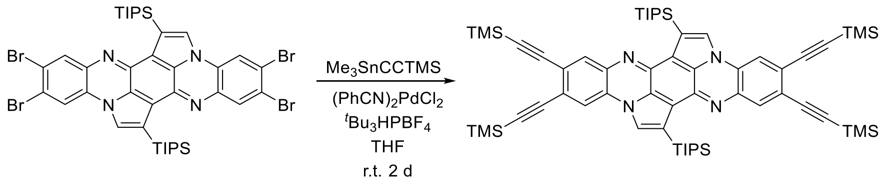

4c **6c**

4c (50.0 mg, 52.1 µmol, 1.00 eq.) was dissolved in 20 mL of dry THF under an argon atmosphere in a flame dried Schlenk tube. The solution was degassed using an argon flow for 30 min. Afterwards, the stannane (204 mg, 781 µmol, 15.0 eq.) was added and the mixture was degassed for additional 10 min. Tri-*tert*-butylphosphine tetrafluoroborate (3.02 mg, 10.4 µmol, 20 mol%) and bis-(benzonitrile)palladium dichloride (2.00 mg, 5.20 µmol, 10 mol%) were added and the mixture was stirred at room temperature for two days. Water was added and the mixture was extracted with DCM. The combined organic layers were dried over $MgSO_4$ and the solvent was removed under reduced pressure. The crude product was purified by flash column chromatography (silica gel, PE:DCM = 9:1 to 4:1) to yield the product (17.0 mg, 16.5 µmol, 32%) as an orange solid.

Mp: >300 °C

R$_f$: 0.45 (silica gel, PE:DCM = 8:2).

^{1}H NMR (600 MHz, $CDCl_3$, 298 K): δ = 7.91–7.90 (m, 4H), 7.80 (s, 2H), 1.97–1.92 (m, 6H), 1.21–1.19 (m, 36H), 0.35 (s, 36H) ppm.

^{13}C{^{1}H} NMR (151 MHz, $CDCl_3$, 298 K): δ = 147.7, 139.1, 133.3, 129.8, 129.2, 128.4, 126.5, 125.4, 124.0, 123.4, 123.0, 121.2, 118.8, 117.9, 103.1, 102.8, 100.4, 98.9, 19.4, 12.4, 0.3, 0.2 ppm.

ATR-IR: $\tilde{\nu}$ [cm^{-1}] = 2941, 2862, 2157, 1609, 1543, 1491, 1460, 1435, 1386, 1292, 1248, 1198, 1147, 1103, 1045, 1022, 993, 913, 840, 759, 685, 661, 647, 634, 614.

HR-MS (MALDI pos.): m/z: [M]$^+$: calcd. for $[C_{60}H_{84}N_4Si_6]^+$: 986.4778; found: 986.4806.

2 NMR Spectra

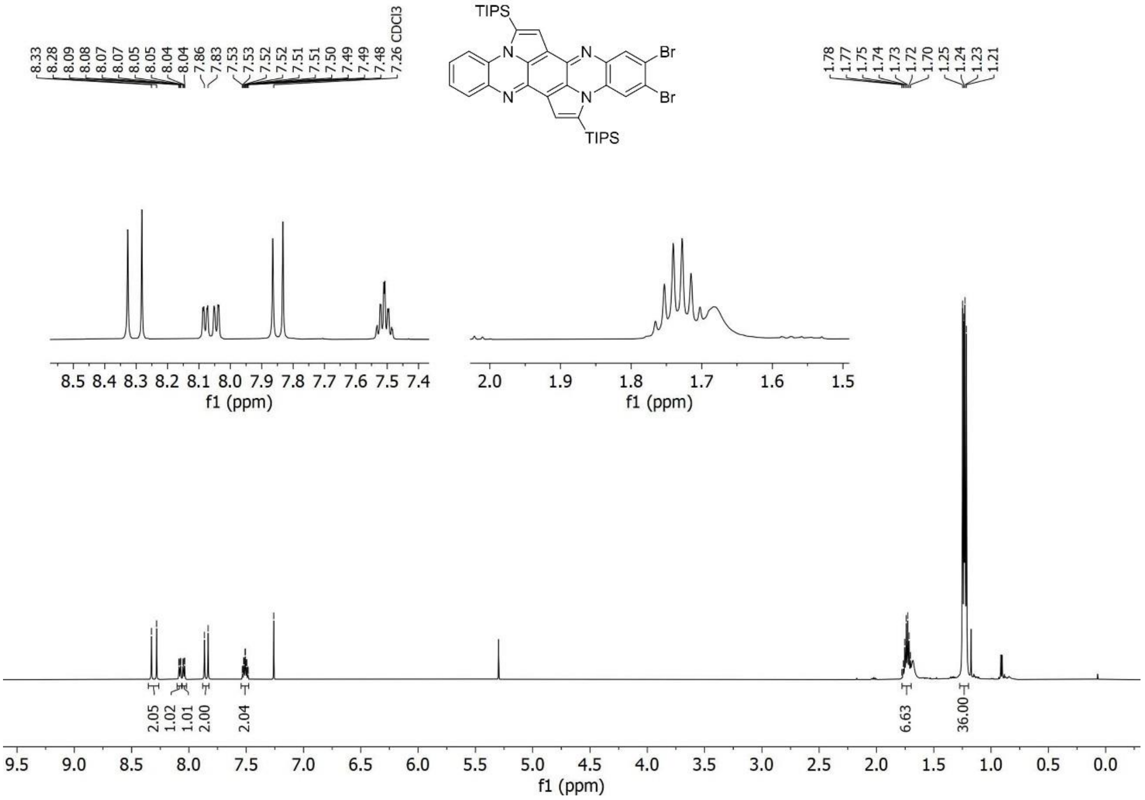

Figure S1. ^{1}H NMR spectrum (600 MHz, $CDCl_3$) of **3a**.

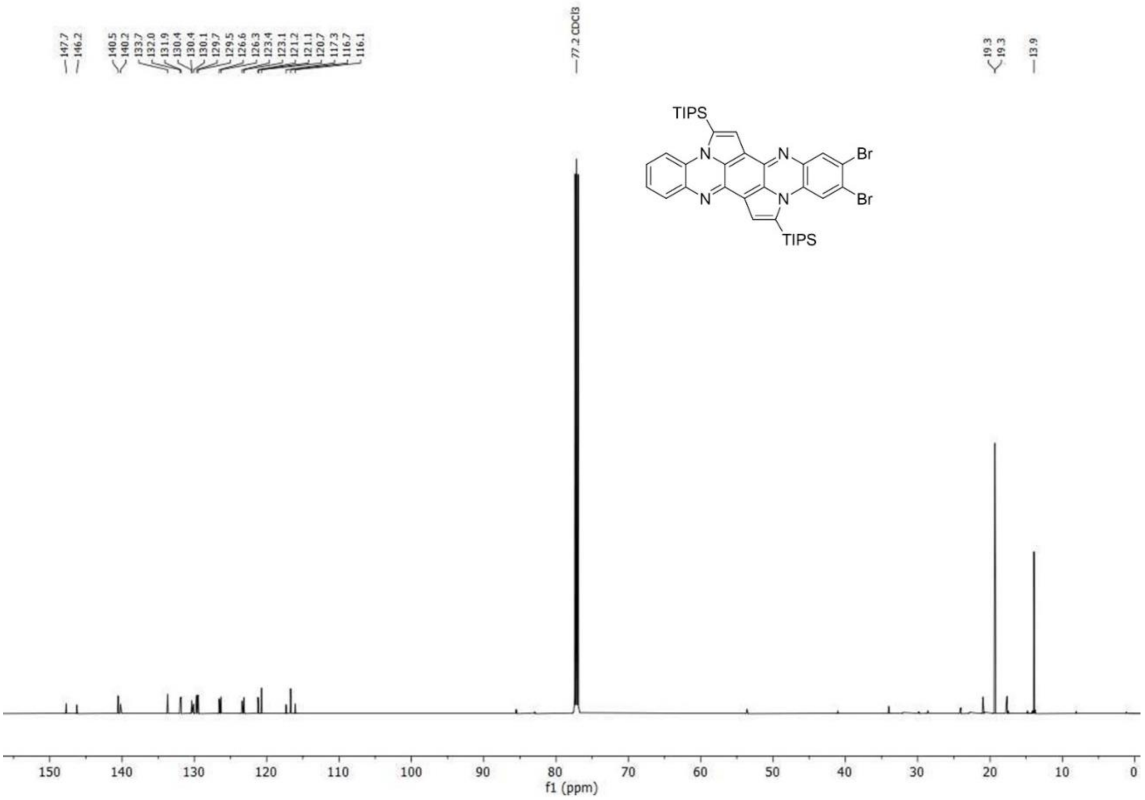

Figure S2. $^{13}C\{^{1}H\}$ NMR spectrum (151 MHz, $CDCl_3$) of **3a**.

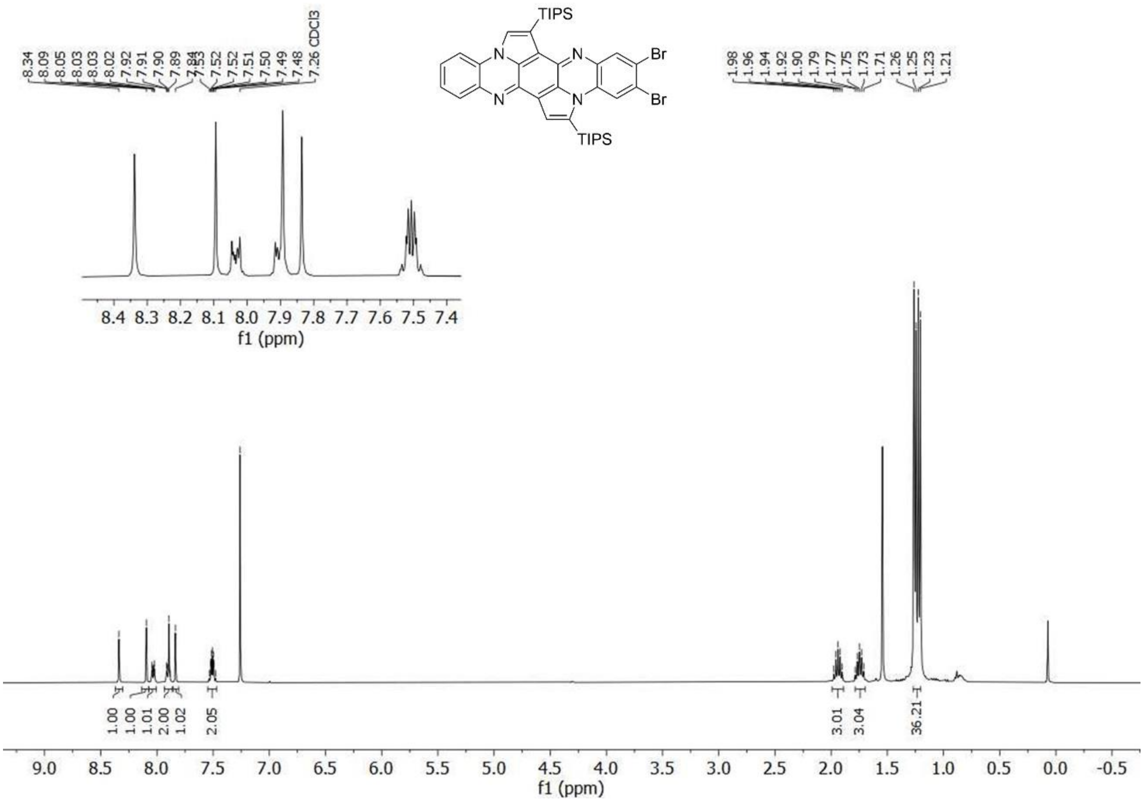

Figure S3. ^{1}H NMR spectrum (400 MHz, $CDCl_3$) of **3b**.

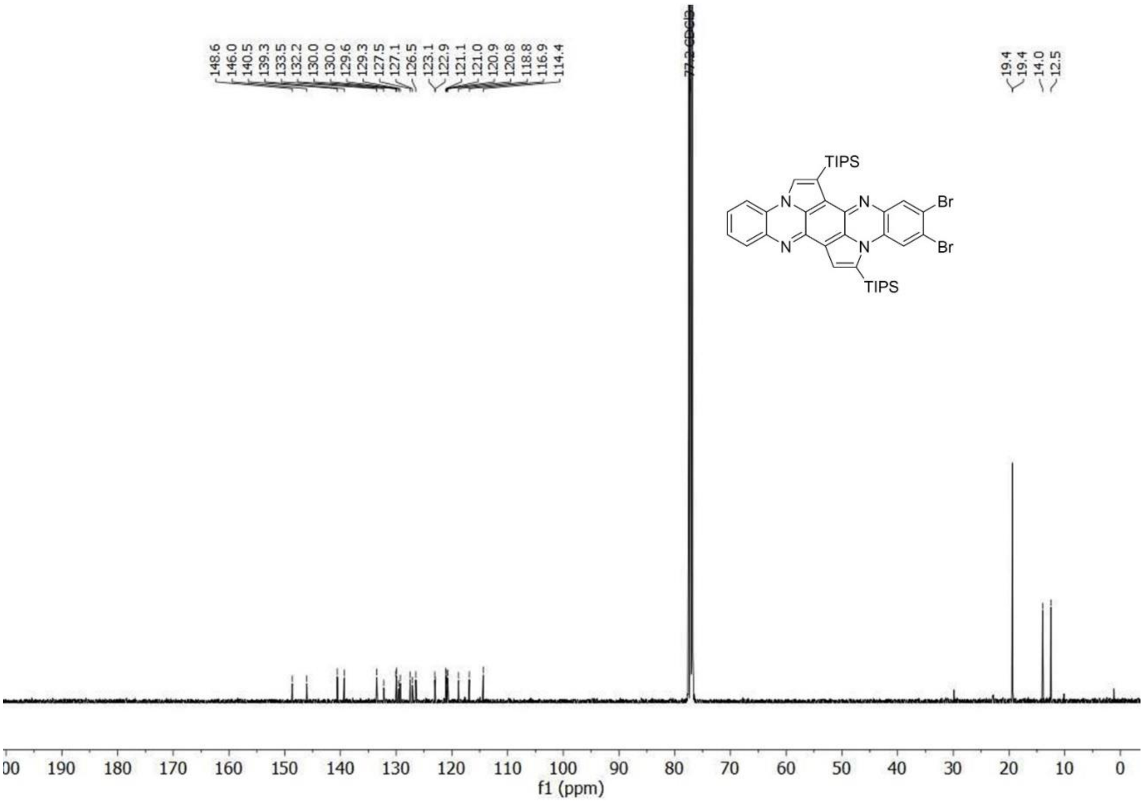

Figure S4. $^{13}C\{^{1}H\}$ NMR spectrum (101 MHz, $CDCl_3$) of **3b**.

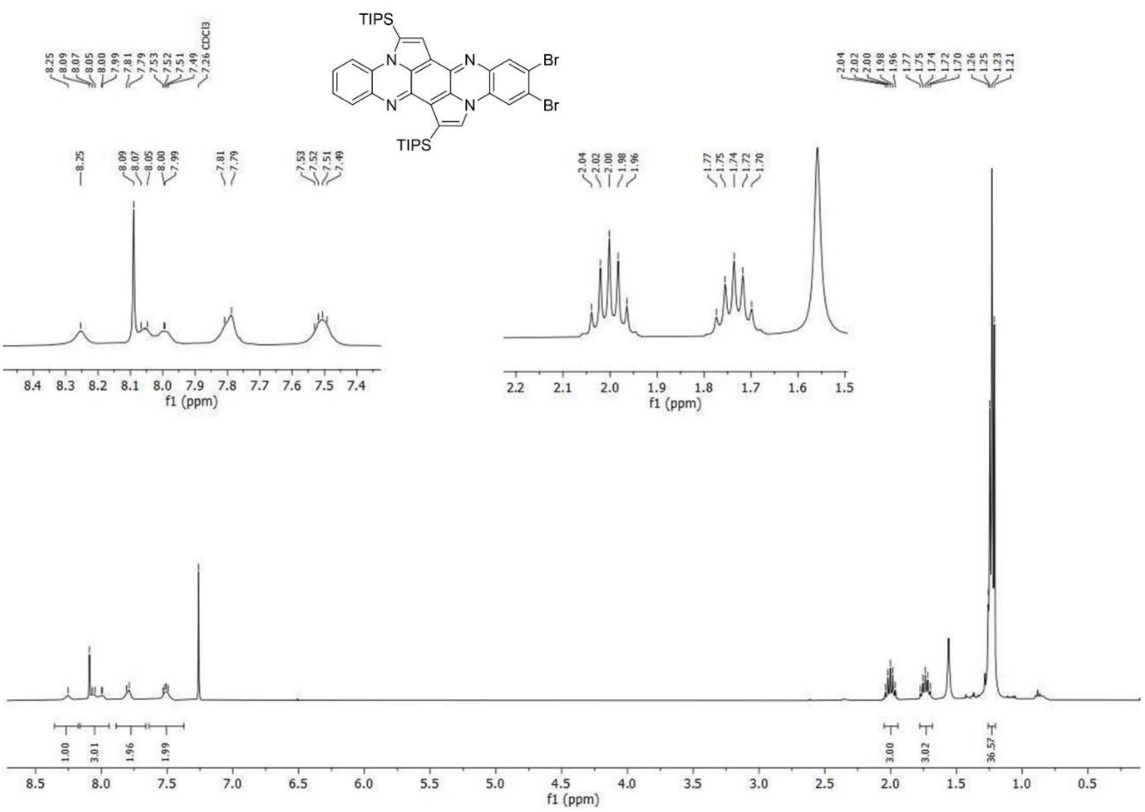

Figure S5. ^{1}H NMR spectrum (400 MHz, $CDCl_3$) of **3c**.

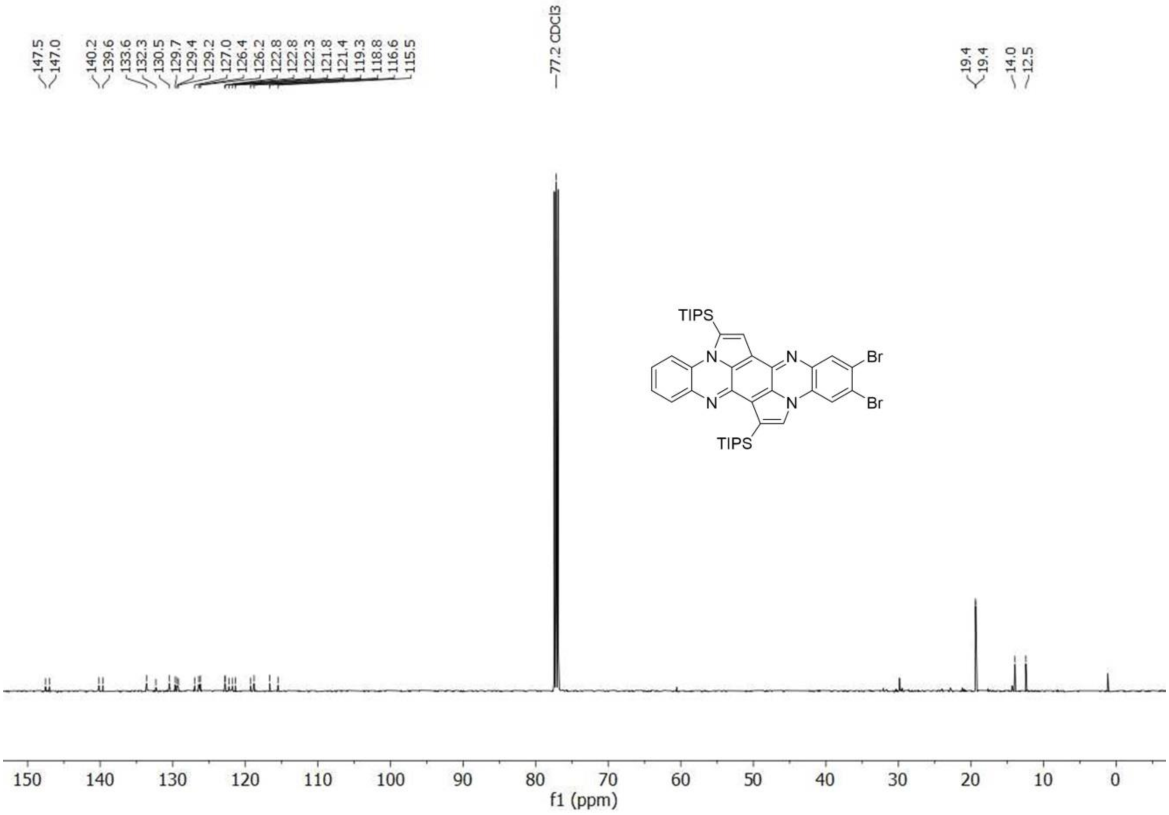

Figure S6. ^{1}H NMR spectrum (400 MHz, $CDCl_3$) of **3c**.

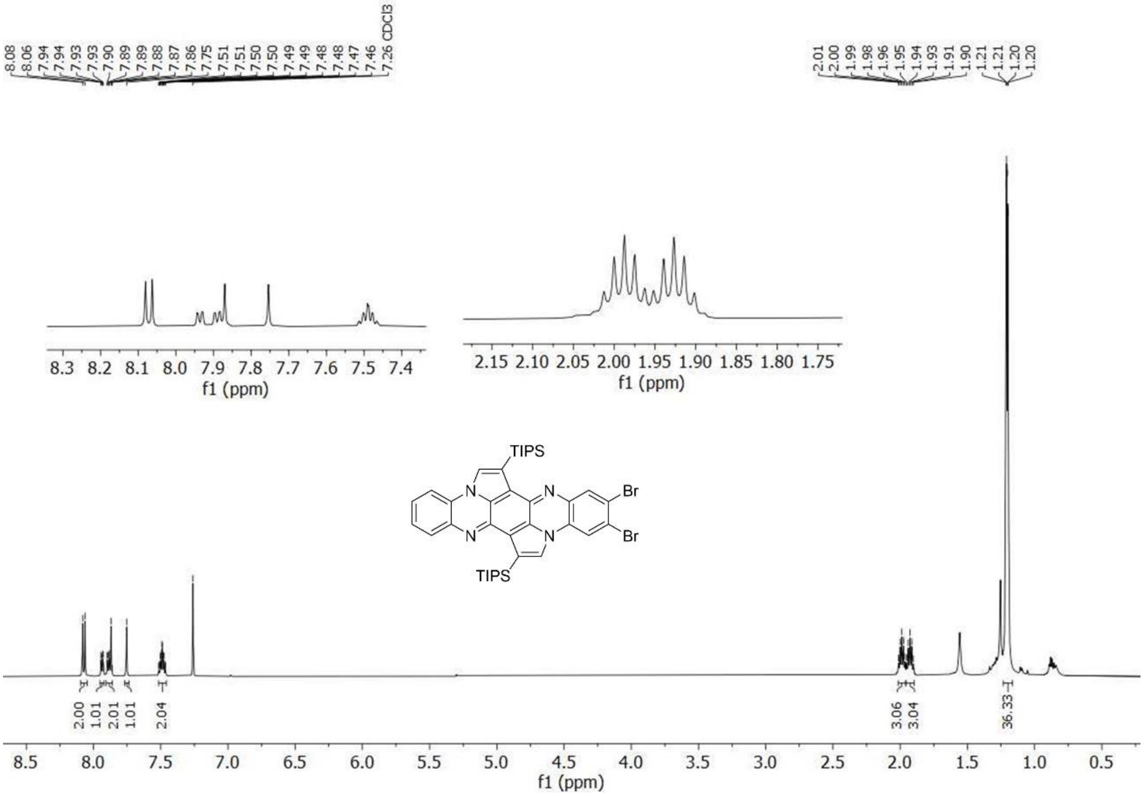

Figure S7. $^{13}C\{^{1}H\}$ spectrum (126 MHz, $CDCl_3$) of **3d**.

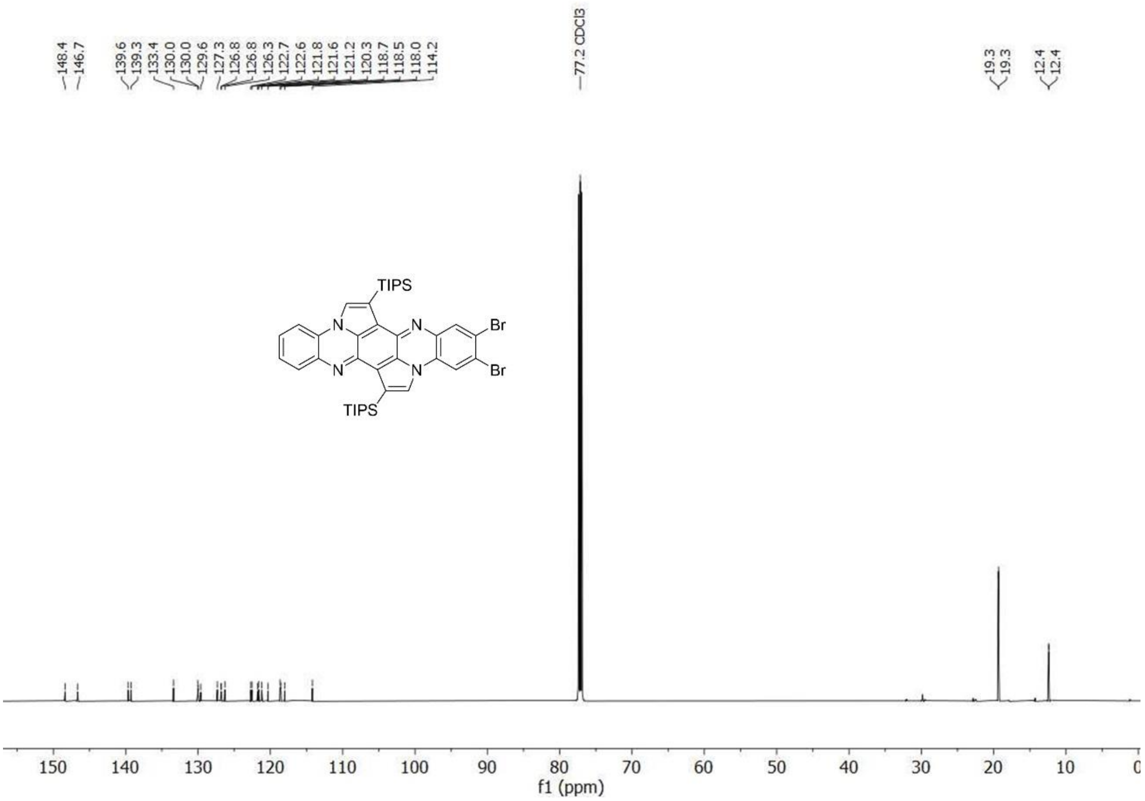

Figure S8. $^{13}C\{^{1}H\}$ NMR spectrum (151 MHz, $CDCl_3$) of **3d**.

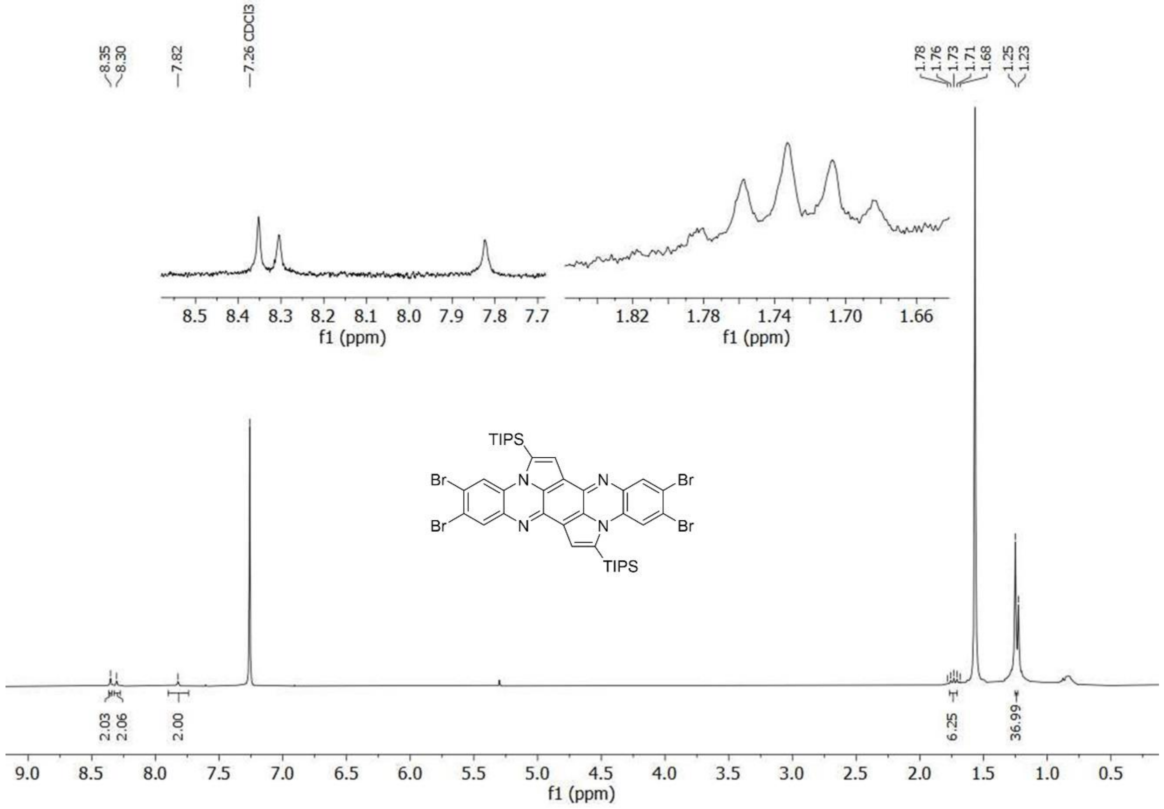

Figure S9. ^{1}H-NMR spectrum (300 MHz, $CDCl_3$) of **4a**.

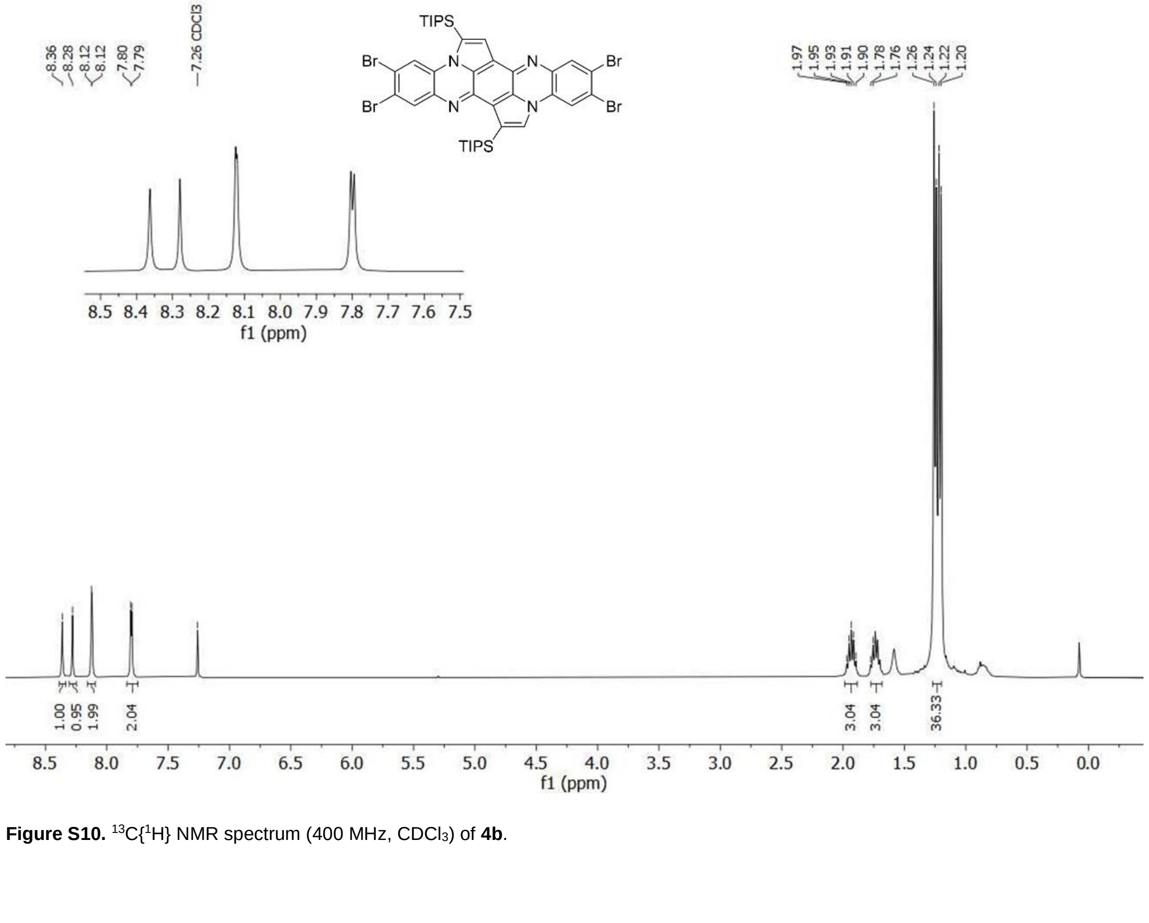

Figure S10. $^{13}C\{^{1}H\}$ NMR spectrum (400 MHz, $CDCl_3$) of **4b**.

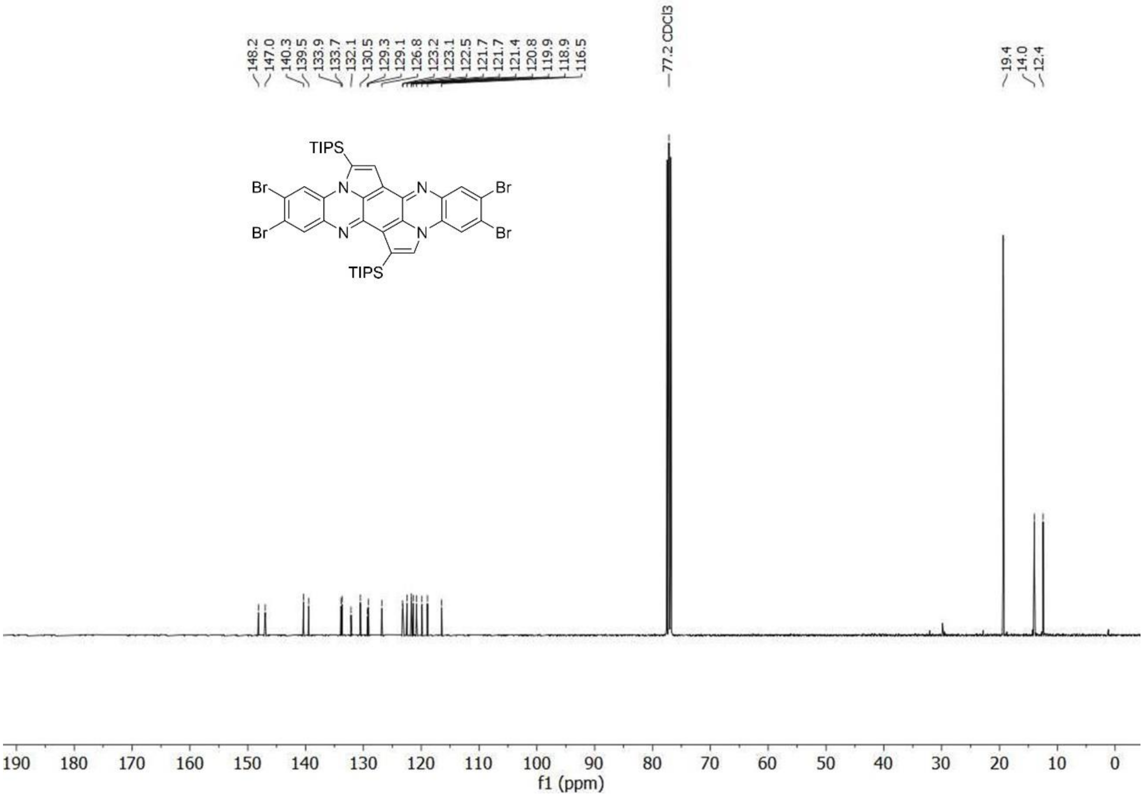

Figure S11. $^{13}C\{^{1}H\}$ NMR spectrum (101 MHz, $CDCl_3$) of **4b**.

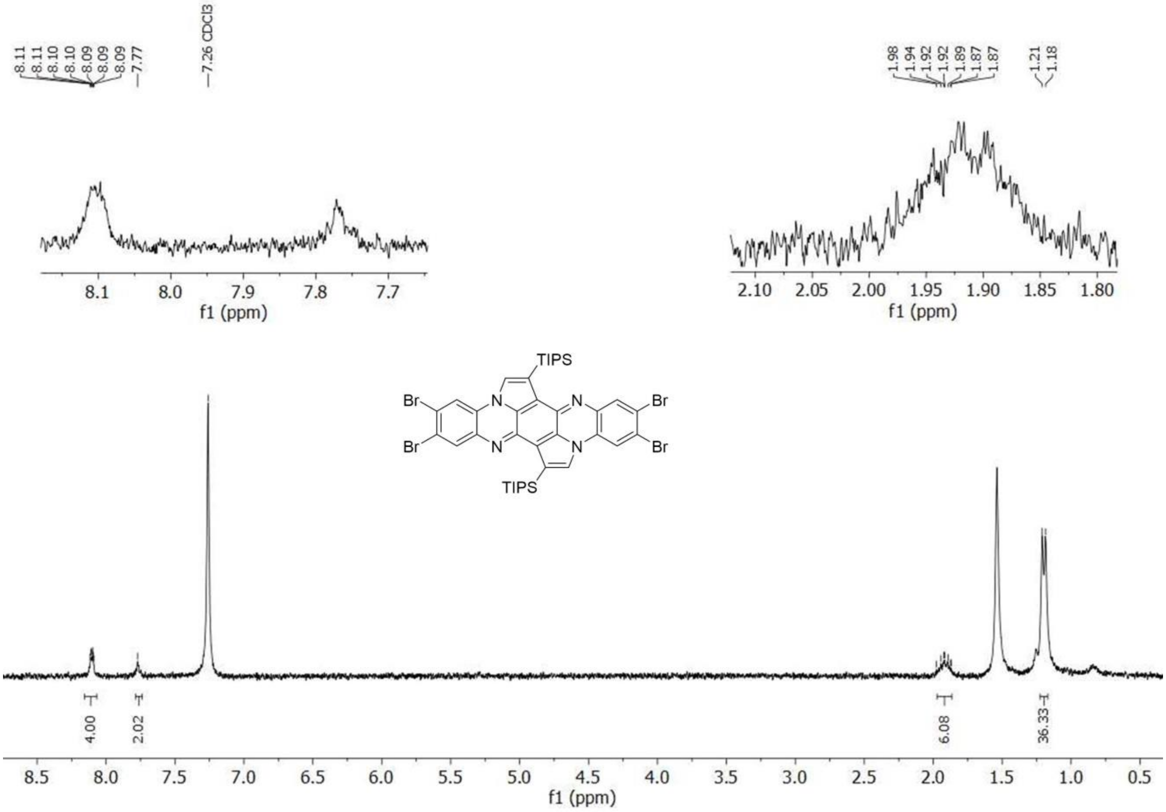

Figure S12. ^{1}H NMR spectrum (300 MHz, $CDCl_3$) of **4c**.

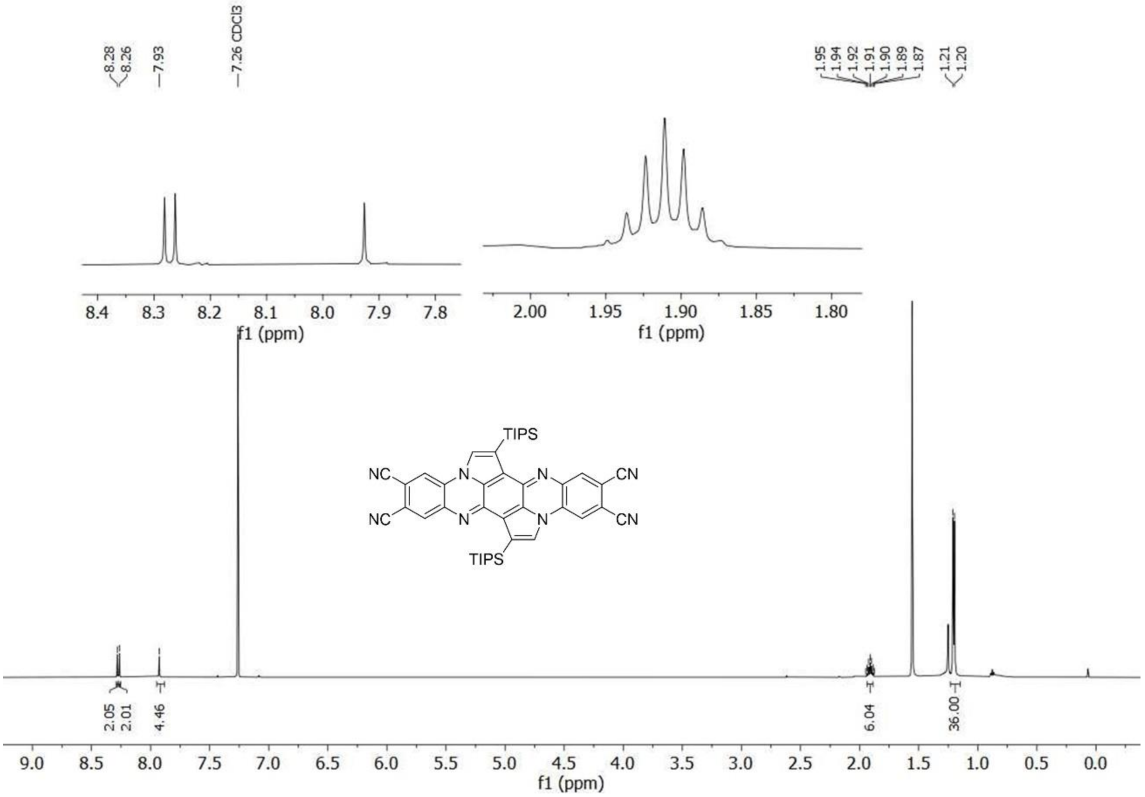

Figure S13. ^{1}H NMR spectrum (600 MHz, $CDCl_3$) of **5c**.

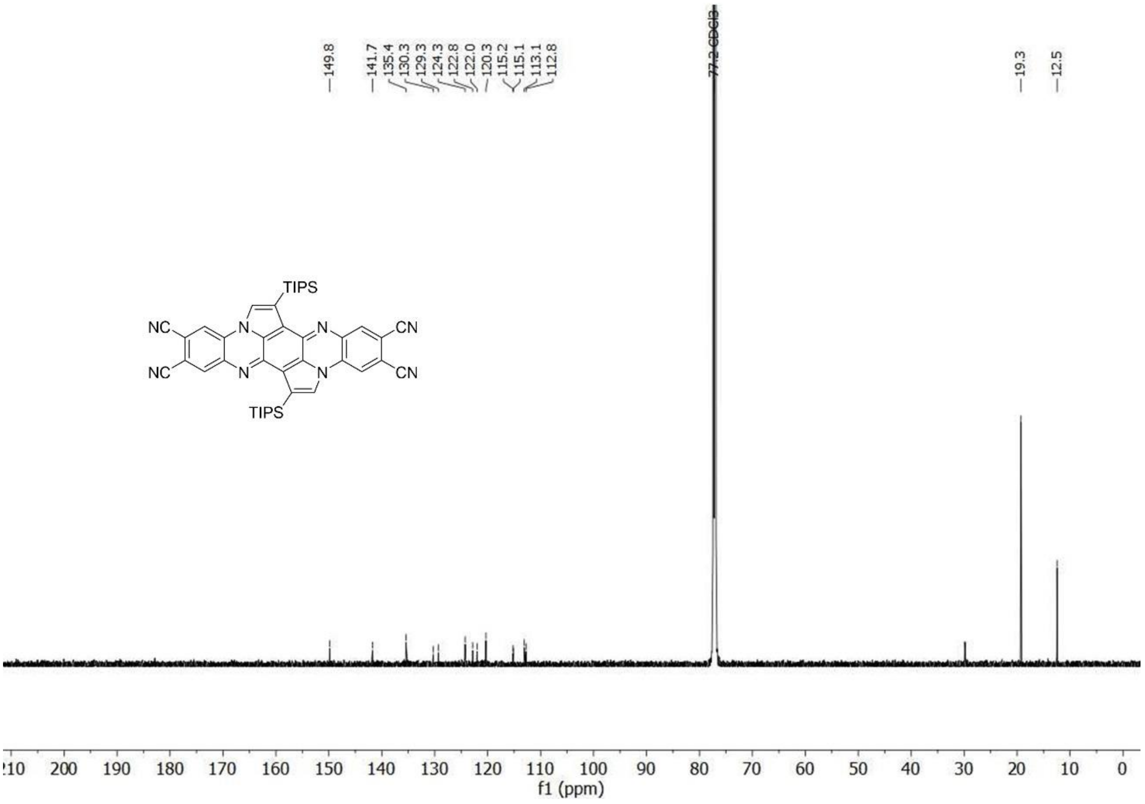

Figure S14. $^{13}C\{^1H\}$ NMR spectrum (151 MHz, $CDCl_3$, 323 K) of **5c**.

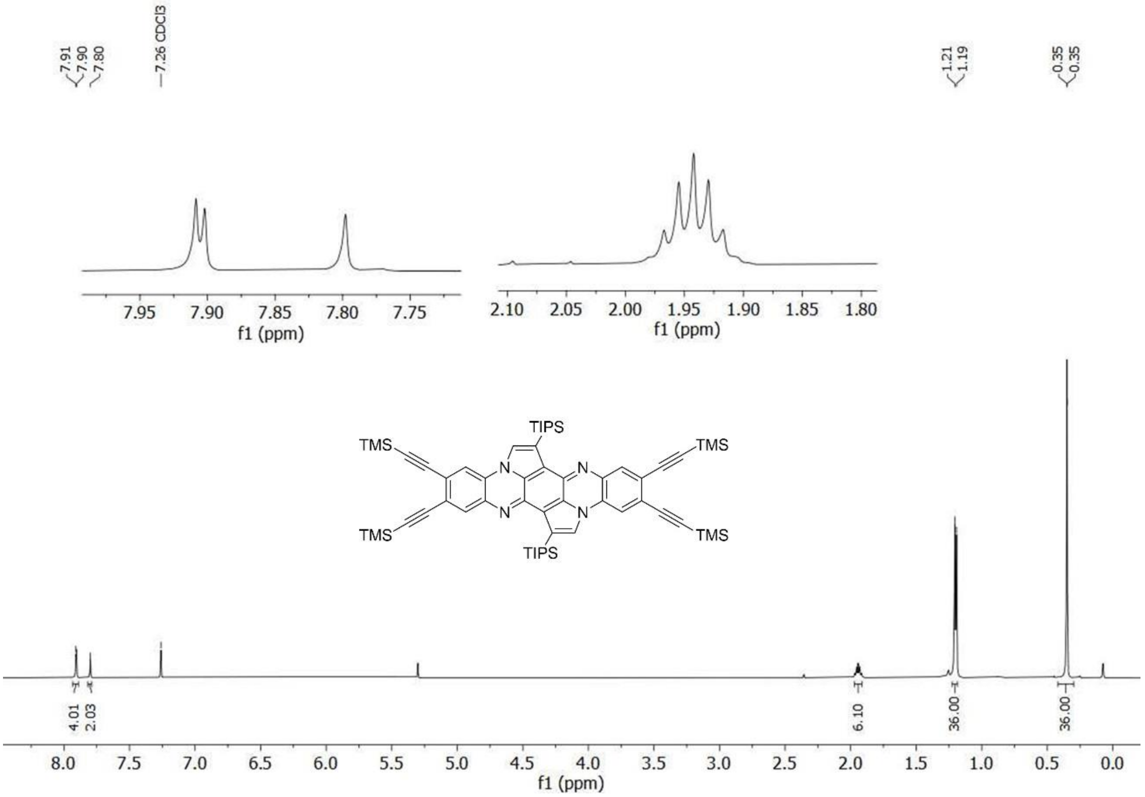

Figure S15. ^{1}H NMR spectrum (600 MHz, $CDCl_3$) of **6c**.

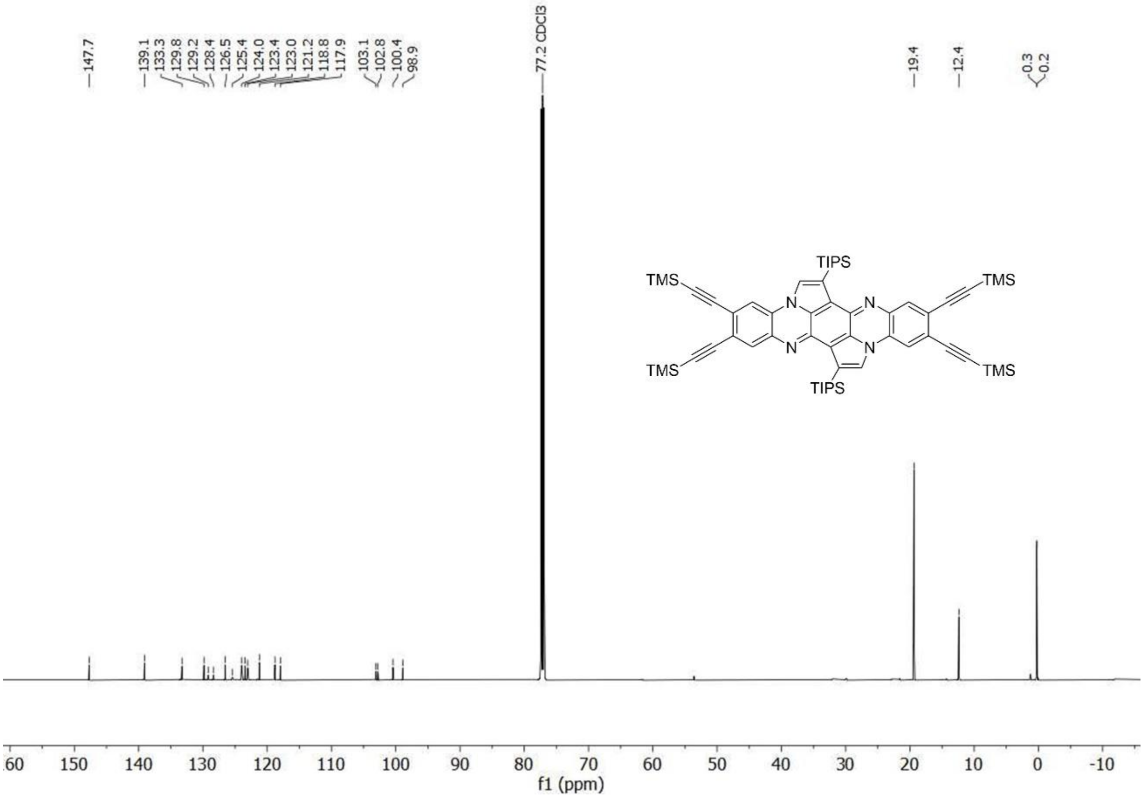

Figure S16. $^{13}C\{^{1}H\}$ NMR spectrum (151 MHz, $CDCl_3$) of **6c**.

3 UV-Vis Spectra

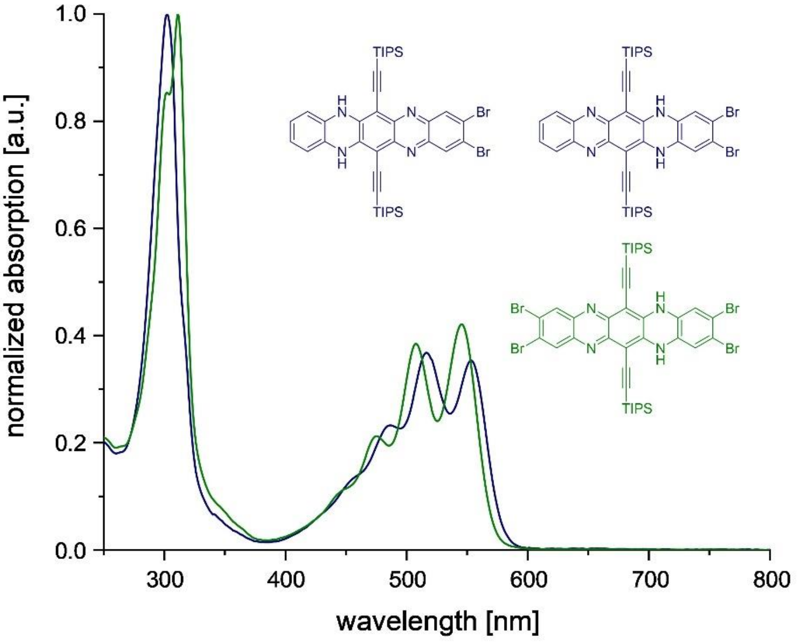

Figure S17. Normalized absorption spectra of **1** and **2** in DCM.

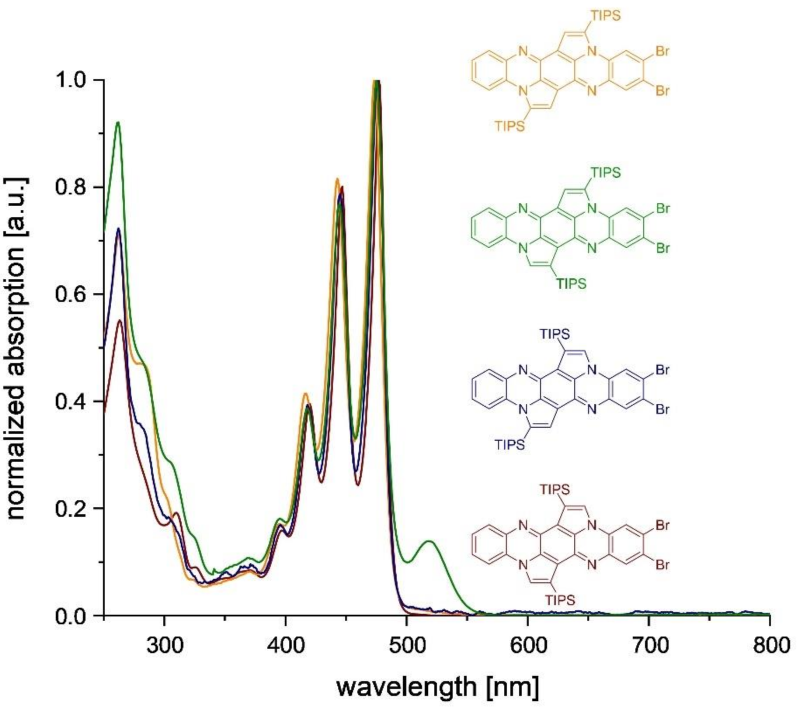

Figure S18. Normalized absorption spectra of **3a**, **3b**, **3c**, and **3d** in DCM.

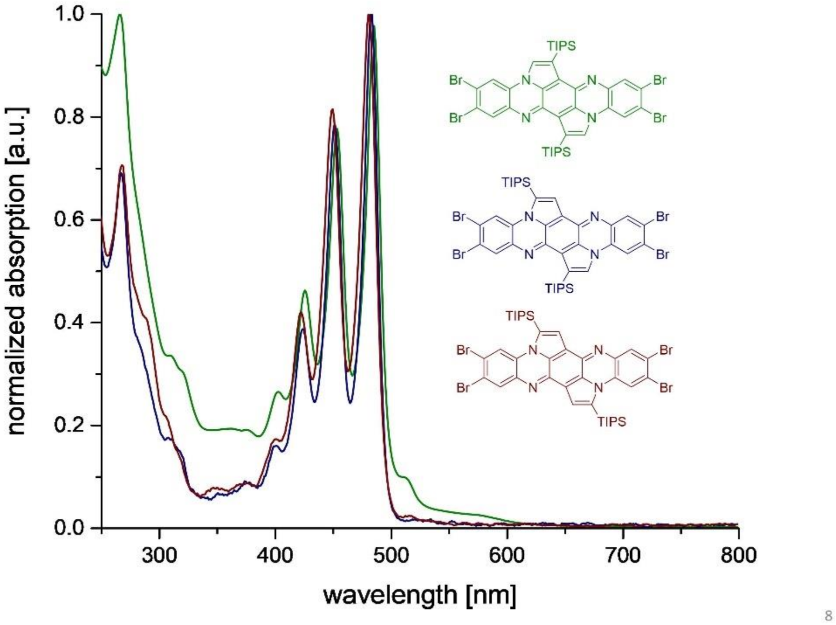

Figure S19. Normalized absorption spectra of **4a**, **4b**, and **4c** in DCM.

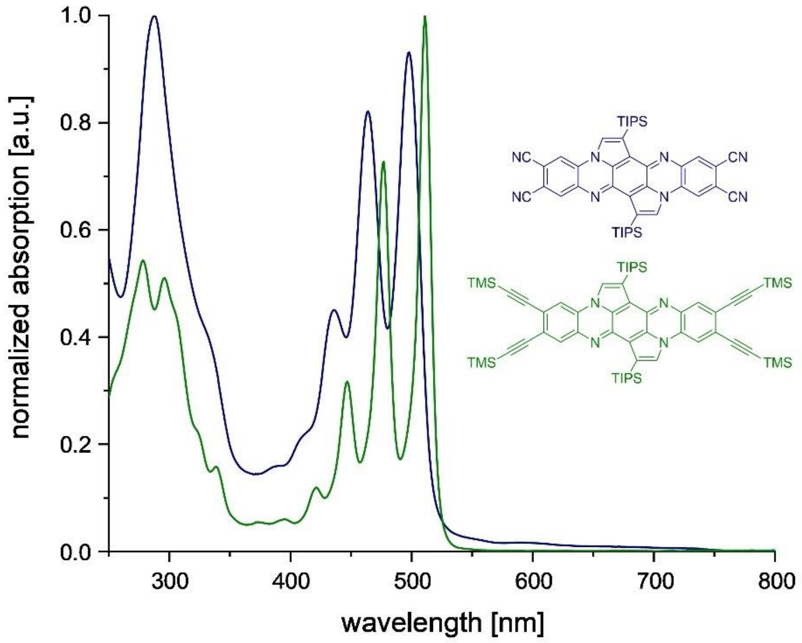

Figure S20. Normalized absorption spectra of **5c** and **6c** in DCM.

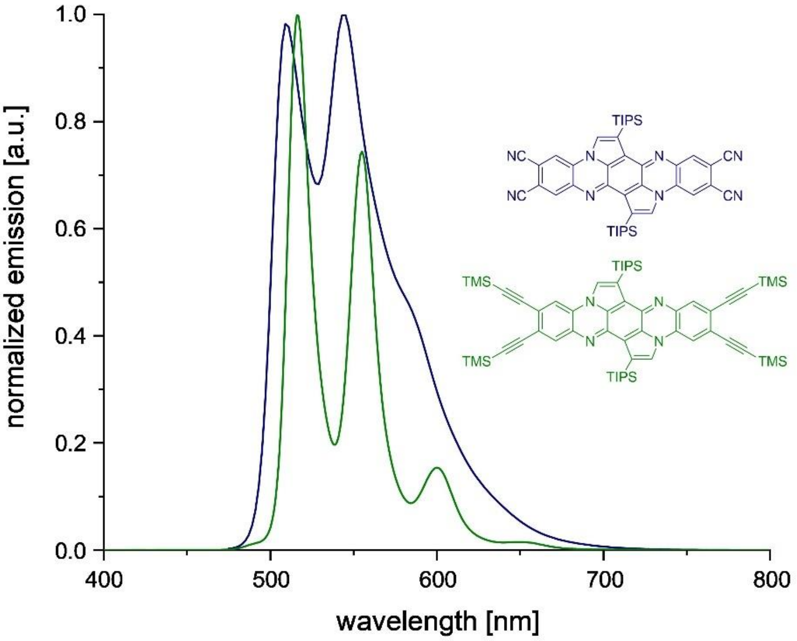

Figure S21. Normalized emission spectra of **1** and **2** in DCM.

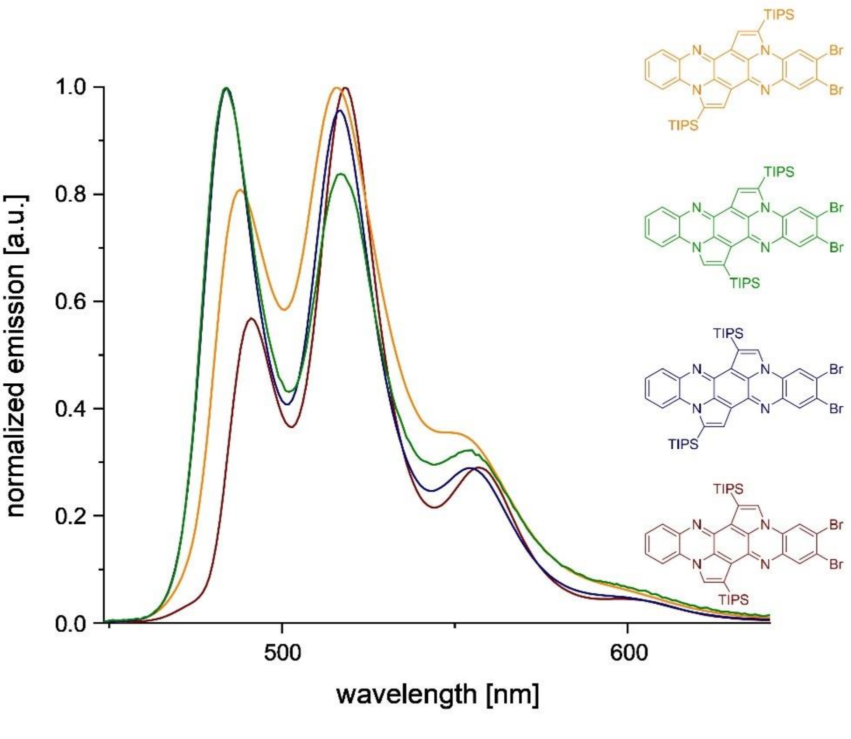

Figure S22. Normalized emission spectra of **3a**, **3b**, **3c**, and **3d** in DCM.

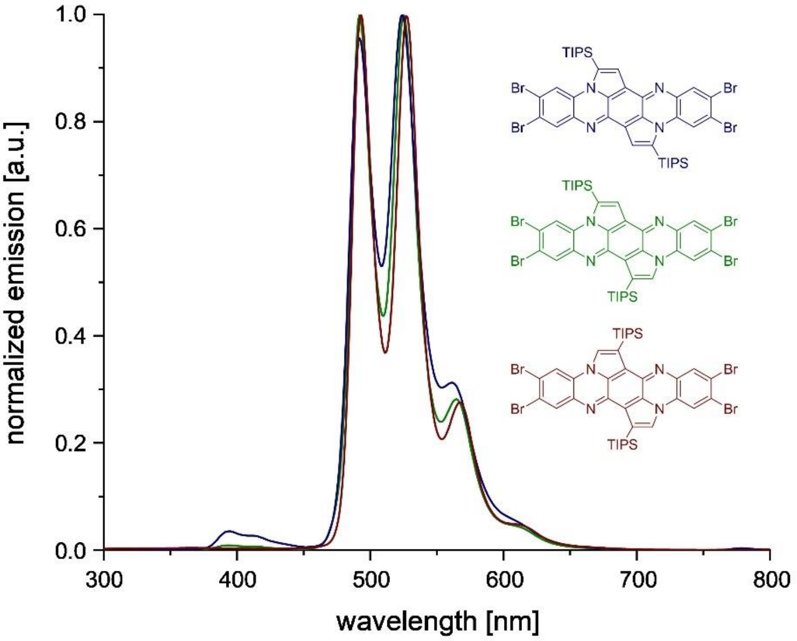

Figure S23. Normalized emission spectra of **4a**, **4b**, and **4c** in DCM.

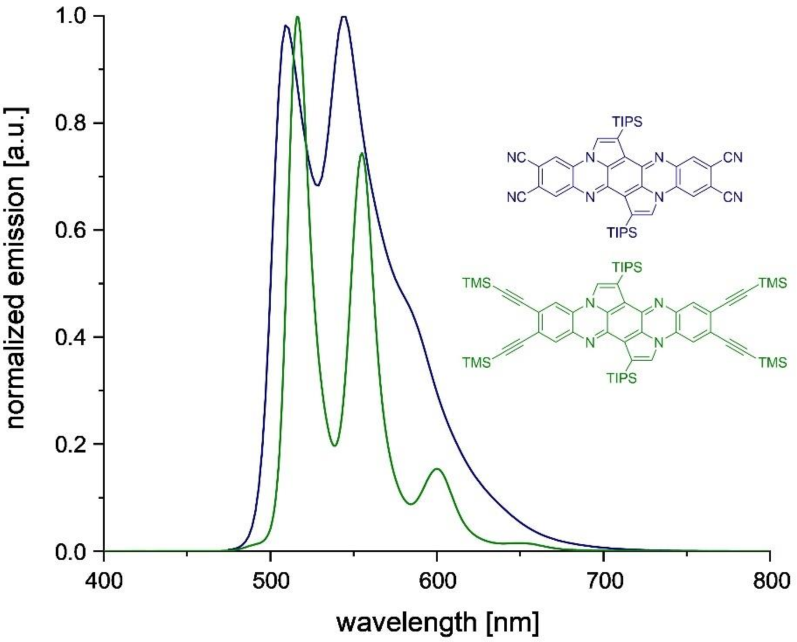

Figure S24. Normalized emission spectra of **5c** and **6c** in DCM.

4 IR Spectra

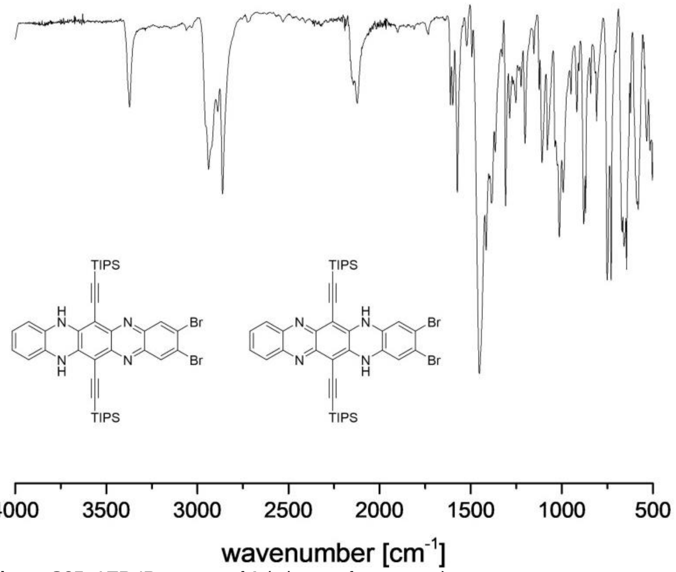

Figure S25. ATR-IR spectra of **1** (mixture of tautomers).

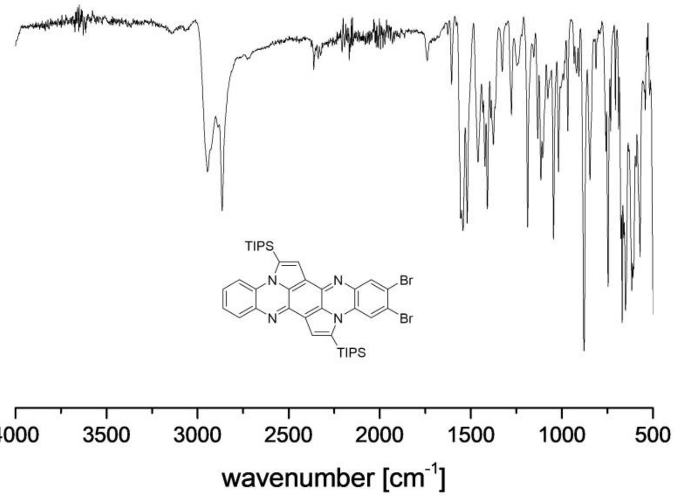

Figure S26. ATR-IR spectra of **3a**.

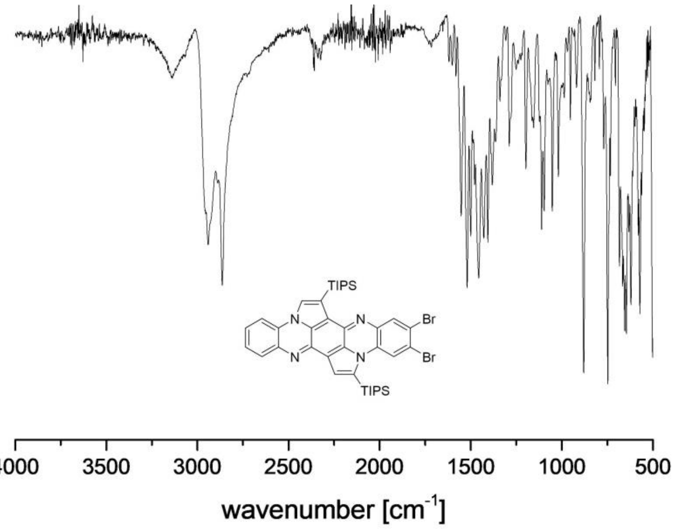

Figure S27. ATR-IR spectra of **3b**.

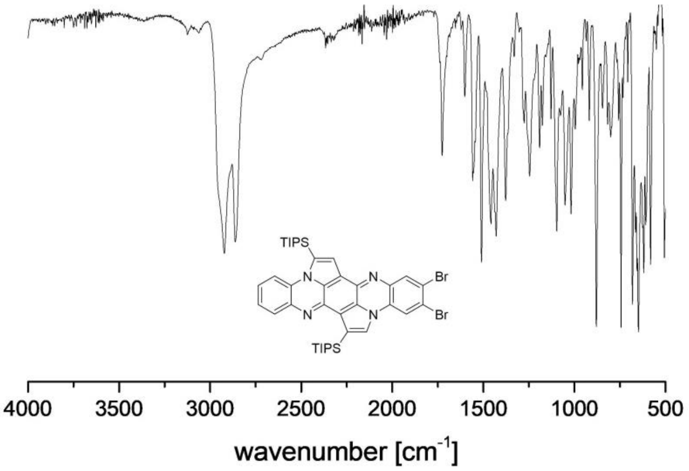

Figure S28. ATR-IR spectra of **3c**.

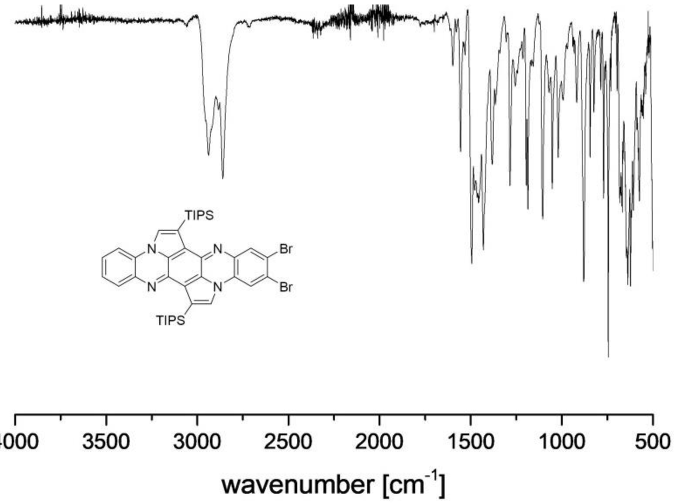

Figure S29. ATR-IR spectra of **3d**.

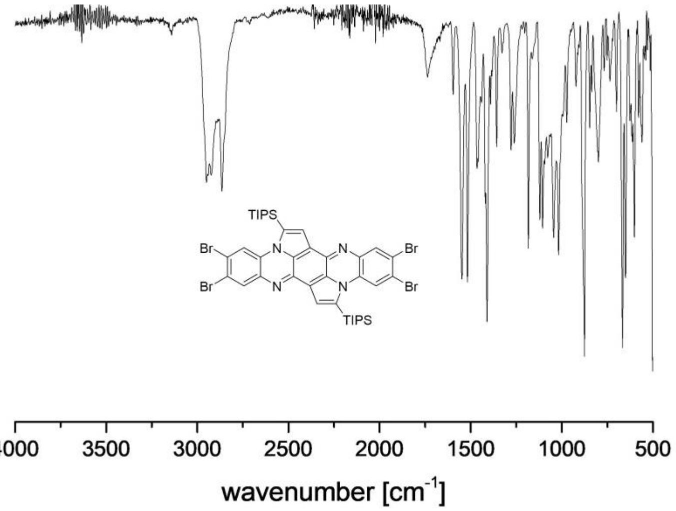

Figure S30. ATR-IR spectra of **4a**.

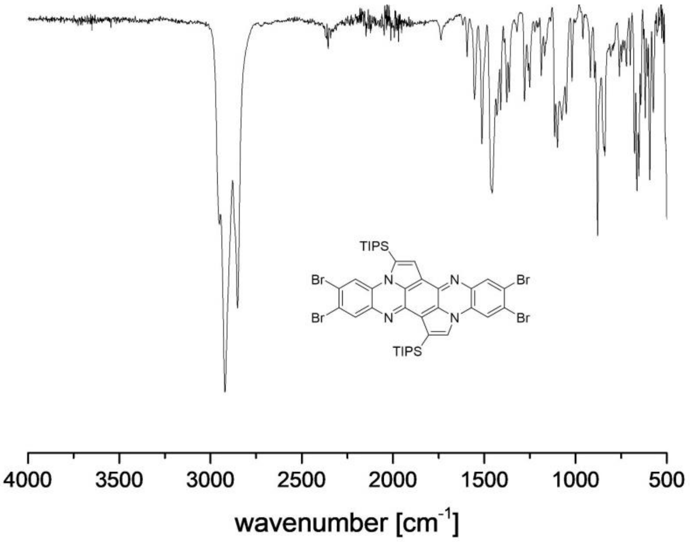

Figure S31. ATR-IR spectra of **4b**.

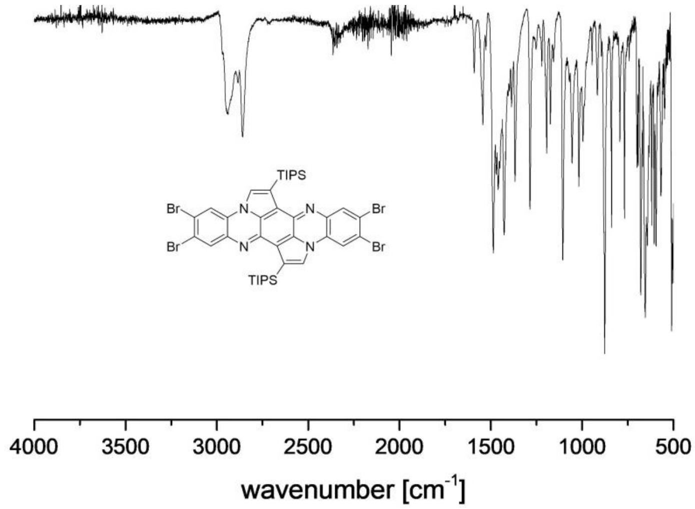

Figure S32. ATR-IR spectra of **4c**.

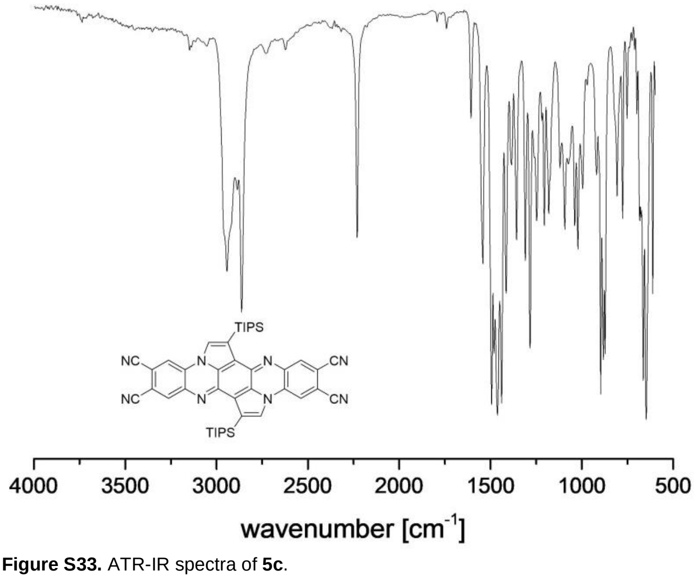

Figure S33. ATR-IR spectra of **5c**.

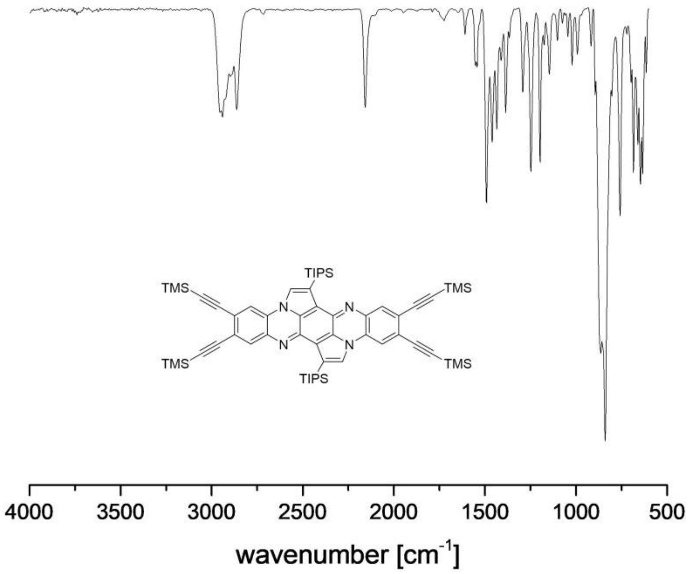

Figure S34. ATR-IR spectra of **6c**.

5 Crystallographic Data

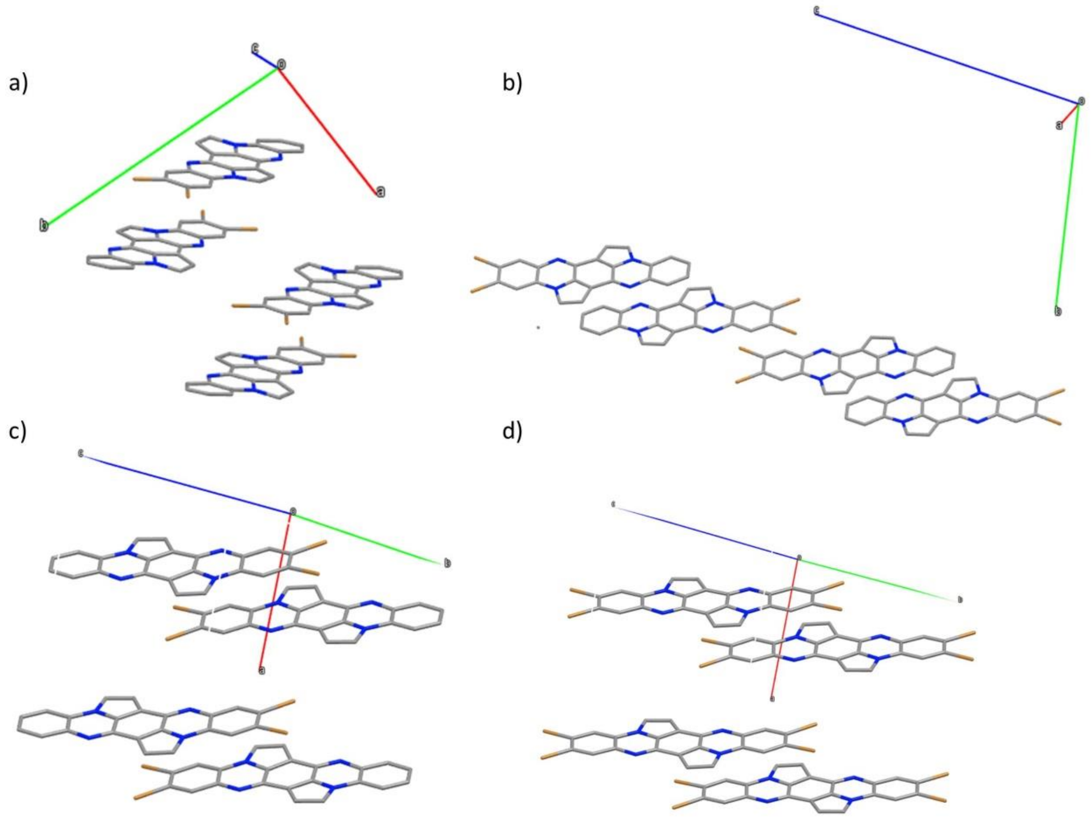

Figure S35. Solid state packing of a) **3a**, b) **3b**, c) **3c** and d) **4b**. Solvent molecules and TIPS-groups were removed for clarity.

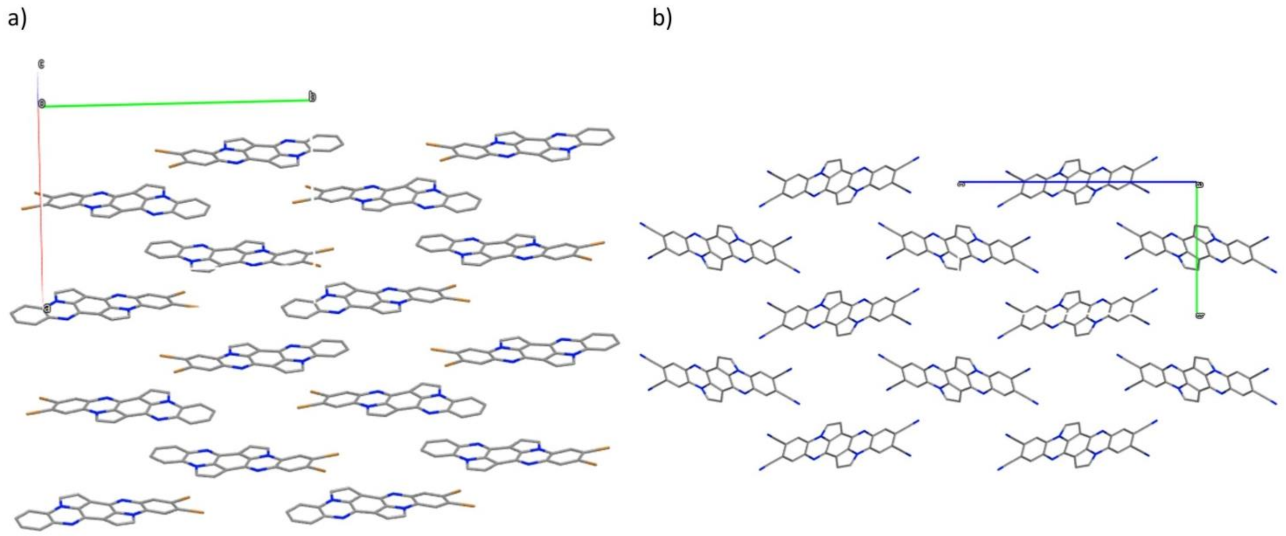

Figure S36. Solid state packing of a) **3d** and b) **5c**. Solvent molecules and TIPS-groups were removed for clarity.

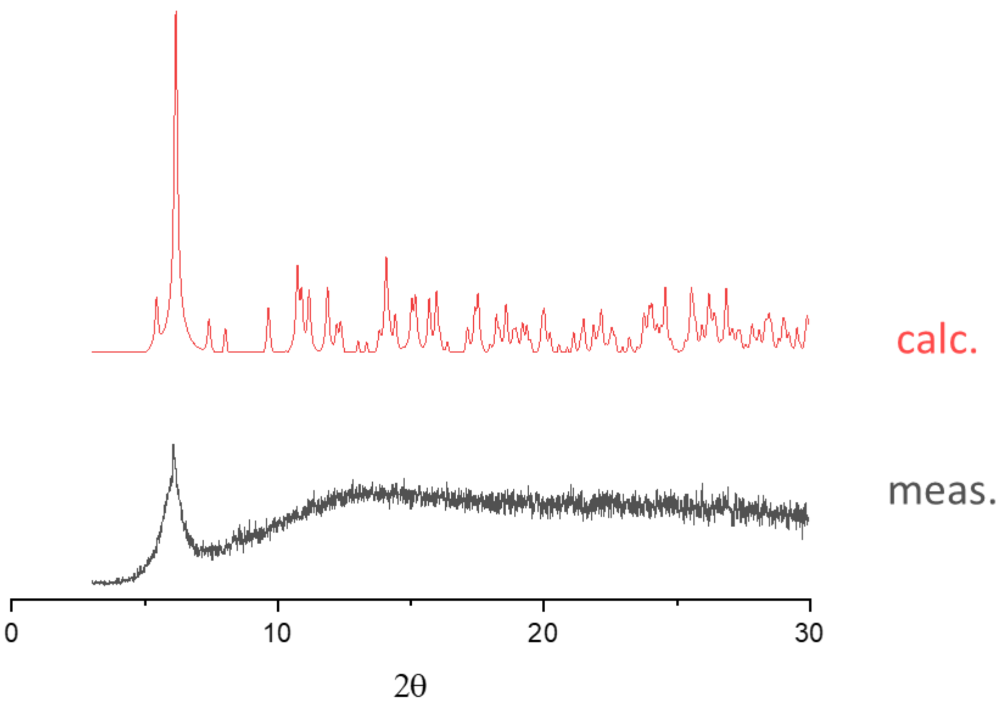

Figure S37. Calculated powder diffractogram and measured XRD film pattern of **3d**. Measured reflex at 2θ = 6.07°.

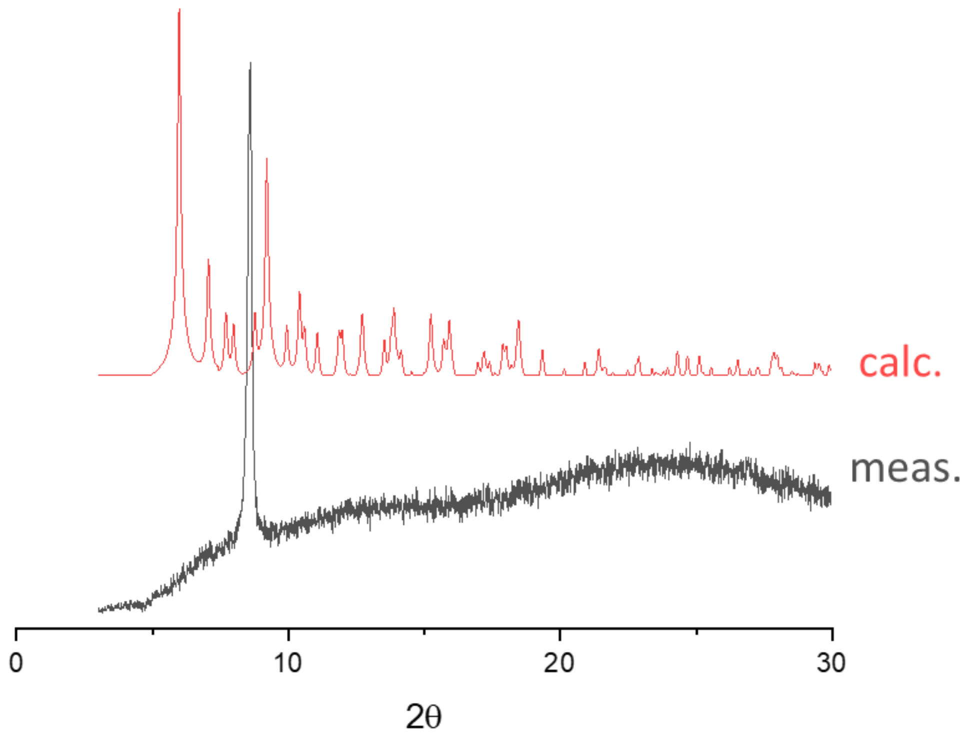

Figure S38. Calculated powder diffractogram and measured XRD film pattern of **5c**. Measured reflex at 2θ = 8.59°.

Table S1. Crystal structure, crystal data and structure refinement of **3a** (2178793).

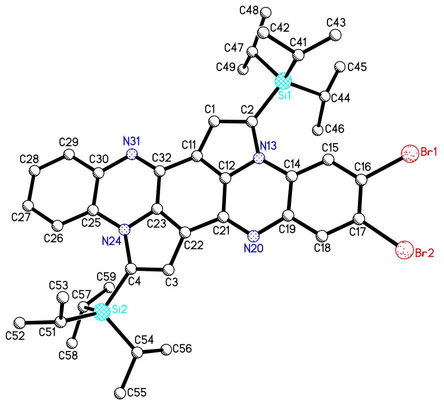

Identification code	hek7	
Empirical formula	$C_{42}H_{55}Br_2Cl_3N_4OSi_2$	
Formula weight	954.25	
Temperature	200(2) K	
Wavelength	1.54178 Å	
Crystal system	triclinic	
Space group	$P\bar{1}$	
Z	2	
Unit cell dimensions	a =8.8455(5) Å	α =70.467(5) deg.
	b = 16.0891(11) Å	β =81.306(5) deg.
	c = 16.7125(9) Å	γ =89.407(5) deg.
Volume	2213.8(2) $Å^3$	
Density (calculated)	1.43 g/cm^3	
Absorption coefficient	4.79 mm^{-1}	
Crystal shape	plate	
Crystal size	0.140 x 0.030 x 0.018 mm^3	
Crystal colour	yellow	
Theta range for data collection	2.8 to 62.1 deg.	
Index ranges	$-9\leq h\leq 10$, $-17\leq k\leq 18$, $-10\leq l\leq 18$	
Reflections collected	16806	
Independent reflections	6611 (R(int) = 0.0717)	
Observed reflections	3518 ($I > 2\sigma(I)$)	
Absorption correction	Semi-empirical from equivalents	
Max. and min. transmission	2.15 and 0.40	
Refinement method	Full-matrix least-squares on F^2	
Data/restraints/parameters	6611 / 434 / 510	
Goodness-of-fit on F^2	0.99	
Final R indices (I>2sigma(I))	R1 = 0.069, wR2 = 0.169	
Largest diff. peak and hole	0.58 and -0.95 $eÅ^{-3}$	

Table S2. Crystal structure, crystal data and structure refinement of **3b** (2178794).

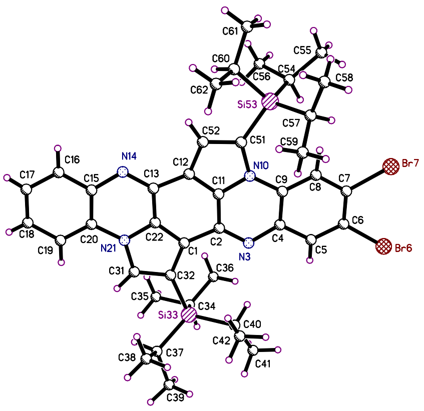

Identification code	hek8	
Empirical formula	$C_{40}H_{50}Br_2N_4Si_2$	
Formula weight	802.84	
Temperature	200(2) K	
Wavelength	0.71073 Å	
Crystal system	Triclinic	
Space group	P-1	
Z	2	
Unit cell dimensions	a =8.3078(5) Å	α =100.677(1) deg.
	b = 13.4793(8) Å	β =91.144(1) deg.
	c = 17.6145(10) Å	γ =90.074(1) deg.
Volume	1938.0(2) $Å^3$	
Density (calculated)	1.38 g/cm^3	
Absorption coefficient	2.19 mm^{-1}	
Crystal shape	plank	
Crystal size	0.170 x 0.054 x 0.040 mm^3	
Crystal colour	brown	
Theta range for data collection	1.2 to 29.9 deg.	
Index ranges	$-11 \le h \le 11$, $-18 \le k \le 18$, $-24 \le l \le 23$	
Reflections collected	39798	
Independent reflections	10362 (R(int) = 0.0341)	
Observed reflections	7920 (I > 2\s(I))	
Absorption correction	Semi-empirical from equivalents	
Max. and min. transmission	0.75 and 0.66	
Refinement method	Full-matrix least-squares on F^2	
Data/restraints/parameters	10362 / 0 / 493	
Goodness-of-fit on F^2	1.03	
Final R indices ($I>2\sigma(I)$)	R1 = 0.037, wR2 = 0.088	
Largest diff. peak and hole	0.82 and -0.55 $eÅ^{-3}$	

Table S3. Crystal structure, crystal data and structure refinement of **3c** (2178795).

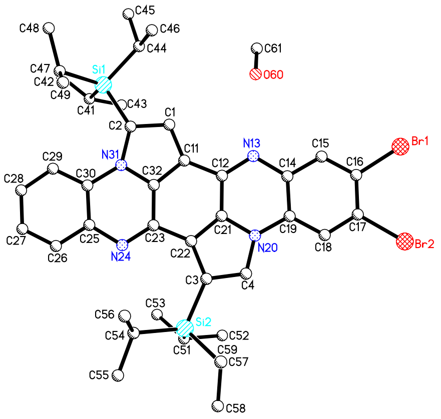

Identification code	hek9	
Empirical formula	$C_{40}H_{50}Br_2N_4O_{0.50}Si_2$	
Formula weight	810.84	
Temperature	200(2) K	
Wavelength	0.71073 Å	
Crystal system	triclinic	
Space group	P&^-&B1	
Z	2	
Unit cell dimensions	a =8.0823(4) Å	α =70.1331(9) deg.
	b = 14.8030(8) Å	β =88.3939(11) deg.
	c = 18.1892(10) Å	γ =81.644(1) deg.
Volume	2024.42(19) $Å^3$	
Density (calculated)	1.33 g/cm^3	
Absorption coefficient	2.10 mm^{-1}	
Crystal shape	rectangular	
Crystal size	0.211 x 0.100 x 0.092 mm^3	
Crystal colour	brown	
Theta range for data collection	1.2 to 26.8 deg.	
Index ranges	$-10 \le h \le 10$, $-18 \le k \le 18$, $-23 \le l \le 23$	
Reflections collected	36522	
Independent reflections	8687 (R(int) = 0.0250)	
Observed reflections	7240 ($I > 2\sigma(I)$)	
Absorption correction	Semi-empirical from equivalents	
Max. and min. transmission	0.85 and 0.76	
Refinement method	Full-matrix least-squares on F^2	
Data/restraints/parameters	8687 / 34 / 476	
Goodness-of-fit on F^2	1.04	
Final R indices (I>2sigma(I))	R1 = 0.031, wR2 = 0.074	
Largest diff. peak and hole	0.45 and -0.34 $eÅ^{-3}$	

Table S4. Crystal structure, crystal data and structure refinement of **3d** (2178796).

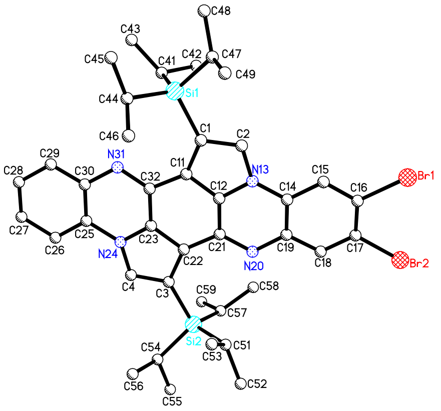

Identification code	mai11sq	
Empirical formula	$C_{40}H_{50}Br_2N_4Si_2$	
Formula weight	802.84	
Temperature	200(2) K	
Wavelength	0.71073 Å	
Crystal system	orthorhombic	
Space group	$Pna2_1$	
Z	8	
Unit cell dimensions	a = 14.9709(6) Å	α = 90 deg.
	b = 19.7156(7) Å	β = 90 deg.
	c = 28.6501(12) Å	γ = 90 deg.
Volume	8456.4(6) $Å^3$	
Density (calculated)	1.26 g/cm^3	
Absorption coefficient	2.01 mm^{-1}	
Crystal shape	plate	
Crystal size	0.107 x 0.075 x 0.020 mm^3	
Crystal colour	yellow	
Theta range for data collection	1.3 to 25.0 deg.	
Index ranges	$-17 \leq h \leq 17$, $-23 \leq k \leq 23$, $-34 \leq l \leq 34$	
Reflections collected	49689	
Independent reflections	14847 (R(int) = 0.1348)	
Observed reflections	8311 ($I > 2\sigma(I)$)	
Absorption correction	Semi-empirical from equivalents	
Max. and min. transmission	0.97 and 0.88	
Refinement method	Full-matrix least-squares on F^2	
Data/restraints/parameters	14847 / 1843 / 868	
Goodness-of-fit on F^2	1.02	
Final R indices (I>2sigma(I))	R1 = 0.083, wR2 = 0.183	
Absolute structure parameter	0.48(2)	
Largest diff. peak and hole	0.72 and -0.83 $eÅ^{-3}$	

Table S5. Crystal structure, crystal data and structure refinement of **4b** (2178797).

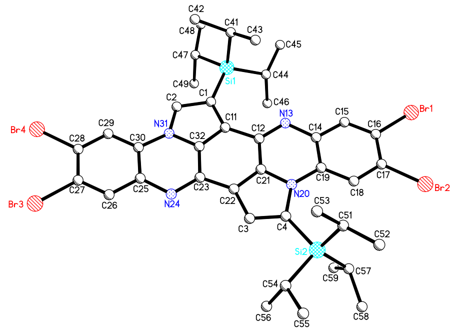

Identification code	hek6	
Empirical formula	$C_{41}H_{52}Br_4N_4OSi_2$	
Formula weight	992.68	
Temperature	200(2) K	
Wavelength	0.71073 Å	
Crystal system	triclinic	
Space group	$P\bar{1}$	
Z	2	
Unit cell dimensions	a =8.0736(9) Å	α =73.515(3) deg.
	b = 14.8486(17) Å	β =87.505(3) deg.
	c = 18.764(2) Å	γ =82.248(3) deg.
Volume	2137.2(4) $Å^3$	
Density (calculated)	1.54 g/cm^3	
Absorption coefficient	3.86 mm^{-1}	
Crystal shape	plate	
Crystal size	0.075 x 0.057 x 0.022 mm^3	
Crystal colour	brown	
Theta range for data collection	1.1 to 23.9 deg.	
Index ranges	$-9 \leq h \leq 9$, $-16 \leq k \leq 16$, $-21 \leq l \leq 21$	
Reflections collected	23667	
Independent reflections	6611 (R(int) = 0.0940)	
Observed reflections	3494 ($I > 2\sigma(I)$)	
Absorption correction	Semi-empirical from equivalents	
Max. and min. transmission	0.93 and 0.79	
Refinement method	Full-matrix least-squares on F^2	
Data/restraints/parameters	6611 / 412 / 471	
Goodness-of-fit on F^2	1.01	
Final R indices (I>2sigma(I))	R1 = 0.060, wR2 = 0.126	
Largest diff. peak and hole	0.81 and -0.47 $eÅ^{-3}$	

Table S6. Crystal structure, crystal data and structure refinement of **5c** (2178798).

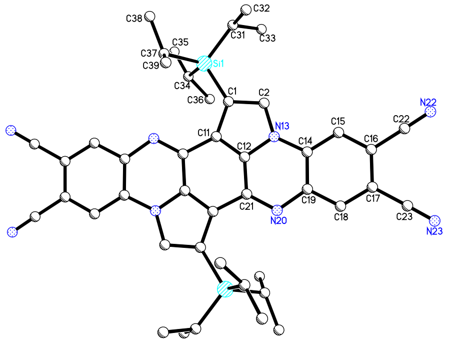

Identification code	hek17	
Empirical formula	$C_{46}H_{50}Cl_6N_8Si_2$	
Formula weight	983.82	
Temperature	200(2) K	
Wavelength	0.71073 Å	
Crystal system	monoclinic	
Space group	$P2_1/n$	
Z	4	
Unit cell dimensions	a = 16.9519(14) Å	α = 90 deg.
	b = 12.6352(10) Å	β =99.9219(19) deg.
	c = 23.2915(19) Å	γ = 90 deg.
Volume	4914.2(7) $Å^3$	
Density (calculated)	1.33 g/cm^3	
Absorption coefficient	0.44 mm^{-1}	
Crystal shape	prism	
Crystal size	0.098 x 0.050 x 0.040 mm^3	
Crystal colour	orange	
Theta range for data collection	1.4 to 25.0 deg.	
Index ranges	$-20\leq h\leq 20$, $-14\leq k\leq 15$, $-27\leq l\leq 27$	
Reflections collected	44952	
Independent reflections	8671 (R(int) = 0.0989)	
Observed reflections	4939 ($I > 2\sigma(I)$)	
Absorption correction	Semi-empirical from equivalents	
Max. and min. transmission	0.96 and 0.91	
Refinement method	Full-matrix least-squares on F^2	
Data/restraints/parameters	8671 / 406 / 597	
Goodness-of-fit on F^2	1.03	
Final R indices (I>2sigma(I))	R1 = 0.084, wR2 = 0.207	
Largest diff. peak and hole	0.92 and -0.85 $eÅ^{-3}$	

6 Computational Details

6.1 Calculations of the Optoelectronic Properties

Calculations were performed using Gaussian 16. TMS groups were used instead of TIPS groups to simplify calculations. First, the gas-phase ground-state equilibrium geometry of the molecules was optimized at the B3LYP/def2-SVP level of theory. Afterwards, the received geometries were refined using the B3LYP/def2-TZVP level of theory. FMO calculations were performed starting from the optimized geometries on the B3LYP/def2-TZVP level of theory.[6]

6.1.1 FMO Calculations

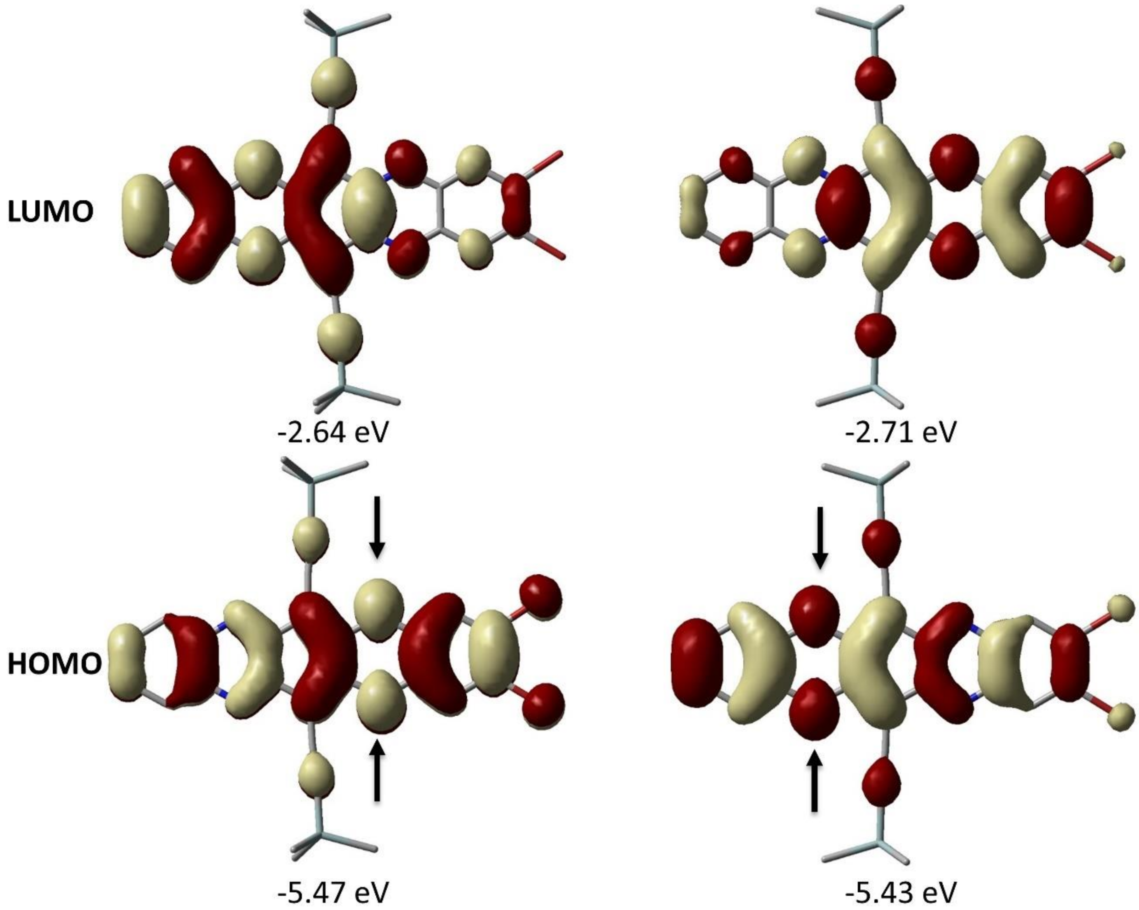

Figure S39. Calculated FMOs of the two isomers of **1**. The position of the nitrogen atoms is marked by arrows.

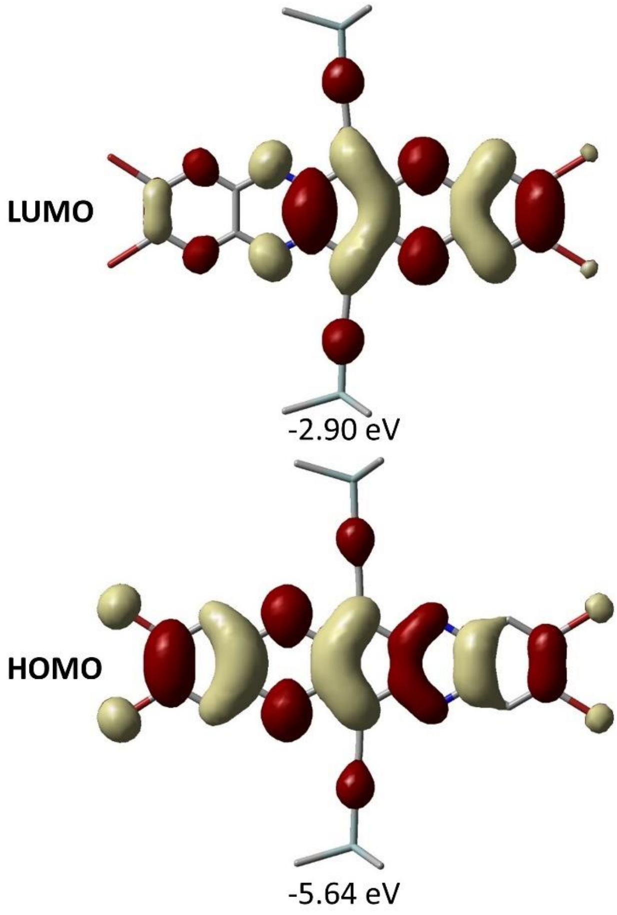

Figure S40. Calculated FMOs of the two isomers of **2**.

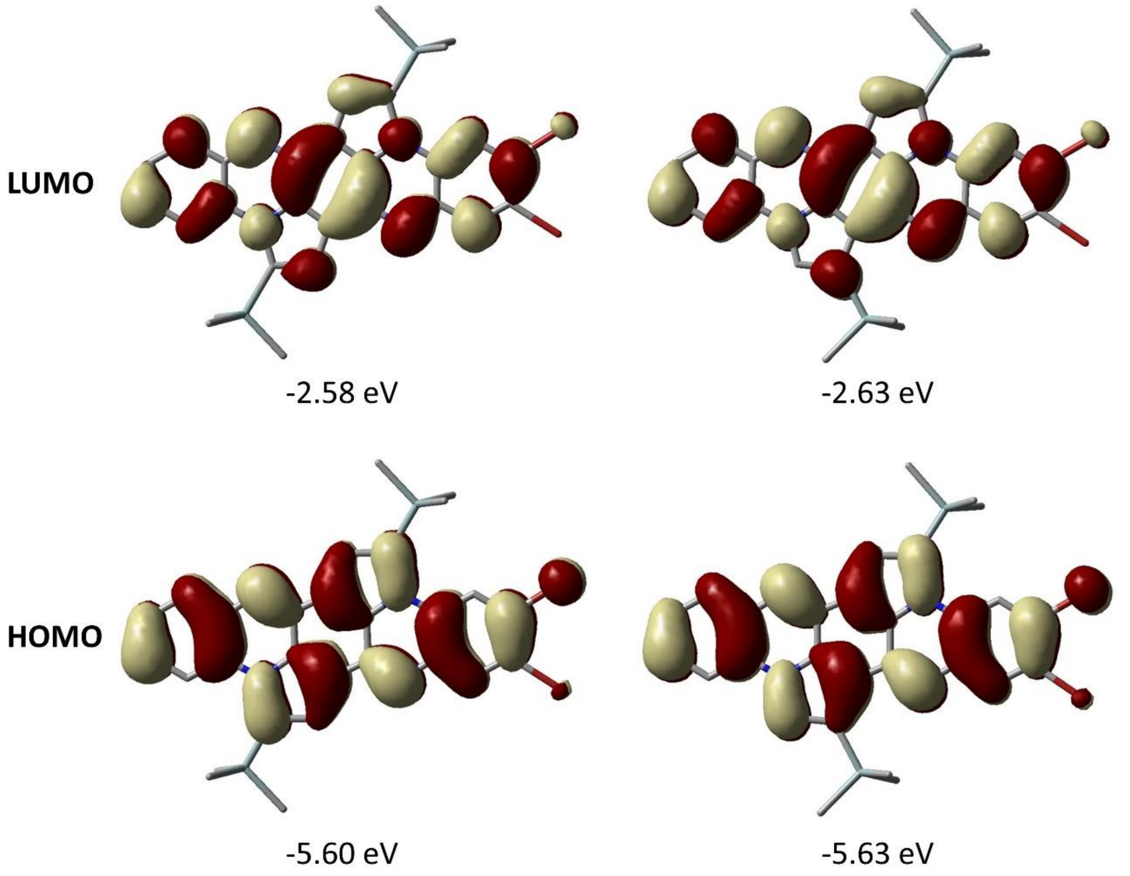

Figure S41. Calculated FMOs. Left: **3a**; Right: **3b**.

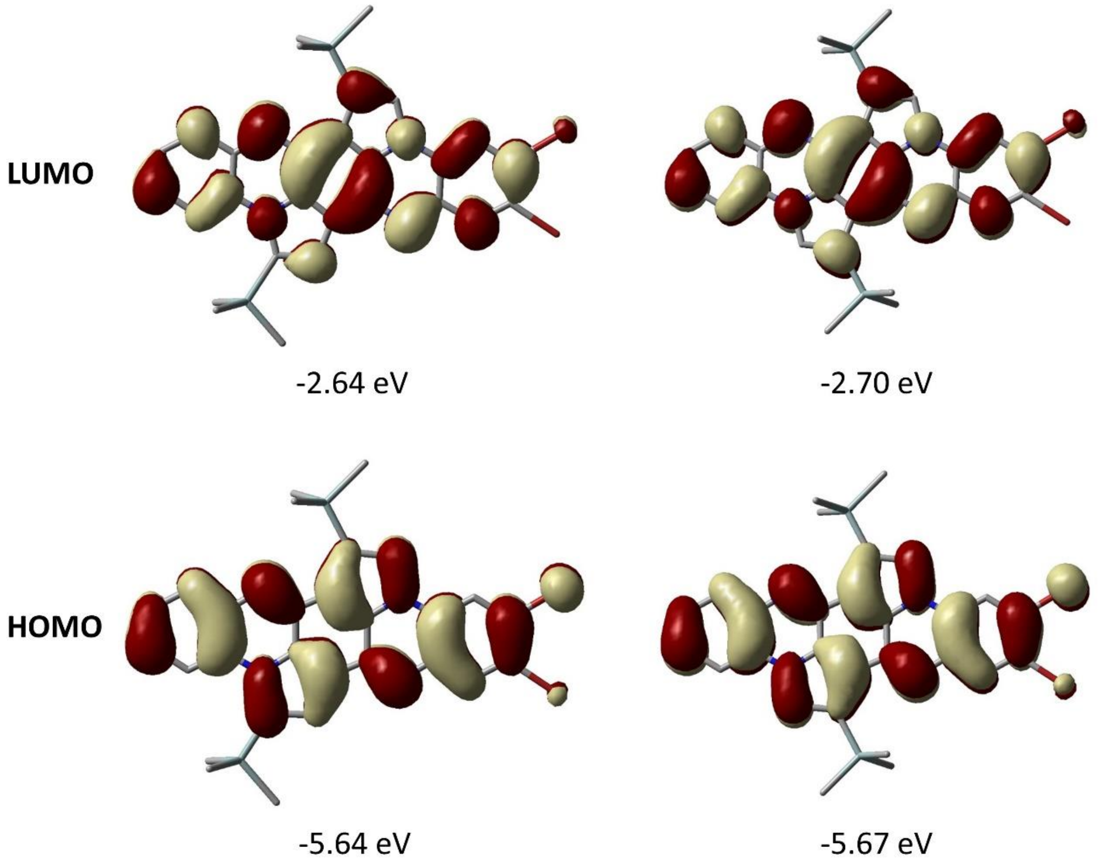

Figure S42. Calculated FMOs. Left: **3c**; Right: **3d**.

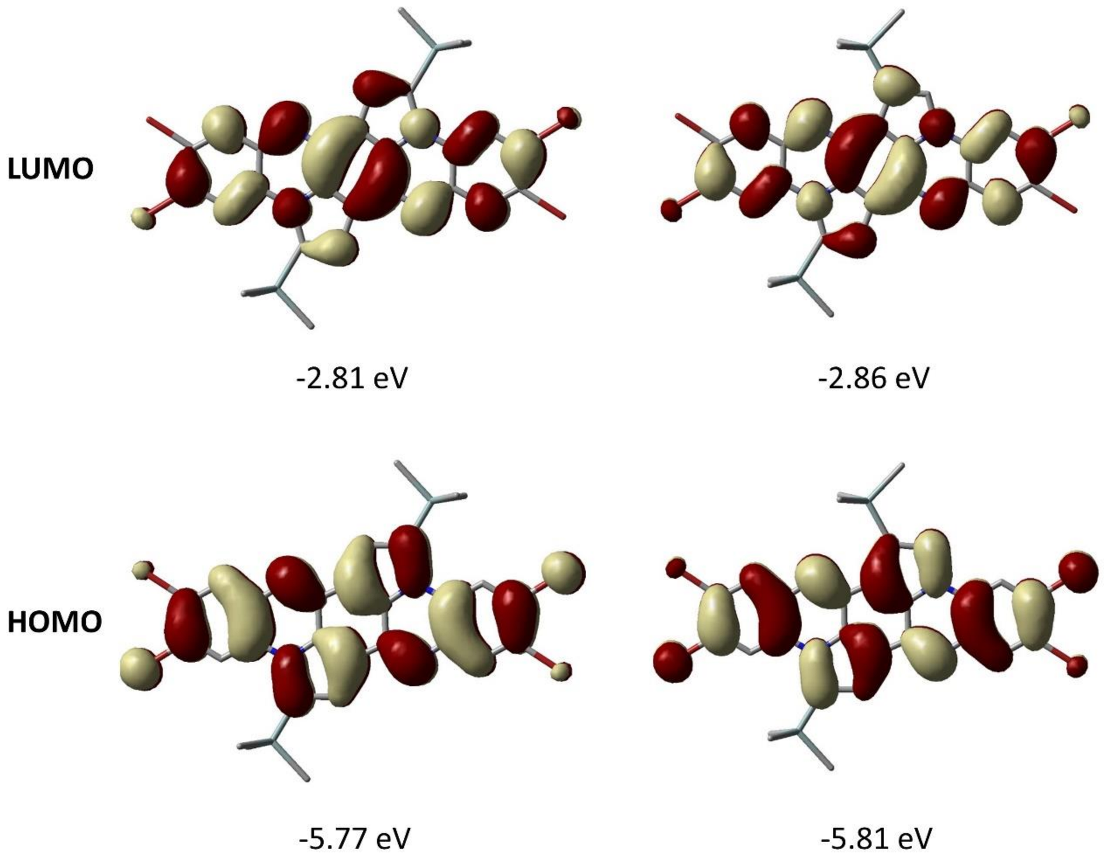

Figure S43. Calculated FMOs. Left: **4a**; Right: **4b**.

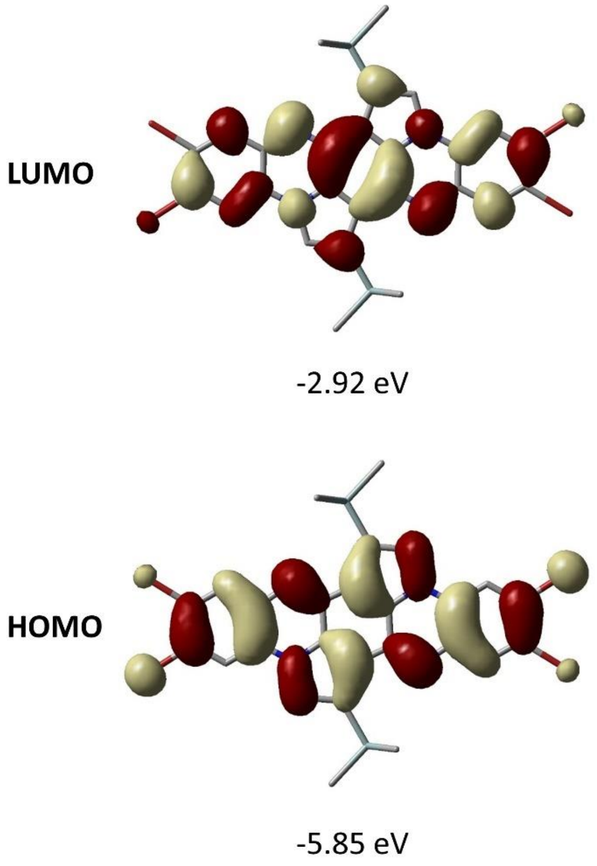

Figure S44. Calculated FMOs of **4c**.

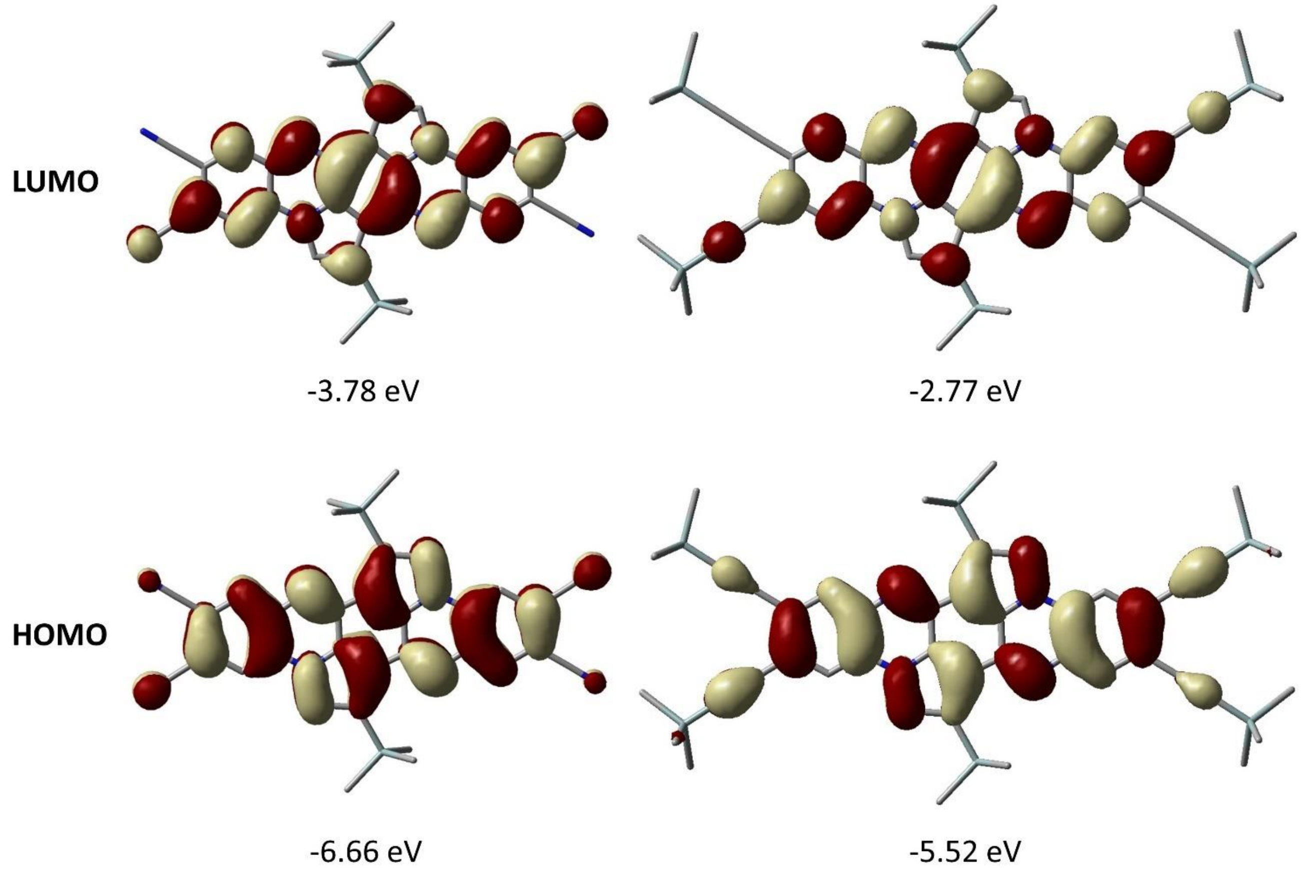

Figure S45. Calculated FMOs. Left: **5c**; Right: **6c**.

6.1.2 Calculation of Theoretical Electron Mobilities

Transfer integrals were calculated using the ADF program.[7] DZ Basis set and GGA PW91 as functional were used. The calculation was performed for each dimer pair of a crystal structure. The used dimers and the corresponding transfer integrals are shown below. Reorganization energies were calculated using the four point method. Therefore a geometry optimization of the isolated monomer in the gas phase was performed for the neutral ($E_{(M)}$) and the anionic species ($E^{-}_{(M-)}$) was performed. Afterwards, single point energy calculations were performed starting from the coordinates of the neutral specie where the charge of the molecule was set negative ($E_{(M-)}$) and starting from the coordinates of the anionic specie where the charge was set neutral ($E^{-}_{(M)}$).[8] A first geometry optimization was performed using Gaussian 16 and the B3LYP/def2svp level of theory. A second geometry optimization as well as single point calculations were performed using Gaussian 16 and the B3LYP/def2tzvp level of theory.

$$\lambda = \lambda_1 + \lambda_2$$
$$\lambda_1 = E_{(M-)} - E_{(M)}$$
$$\lambda_2 = E^{-}_{(M)} - E^{-}_{(M-)}$$

The reorganization energies and the transfer integrals were used to calculate the electron transfer rate using the Marcus theory.[9] The results for all possible transfer paths are summarized in the figures below.

$$k_{ET} = \frac{4\pi}{h} \frac{V^2}{\sqrt{4\pi k_b T}} e^{-\frac{\lambda}{4k_b T}}$$

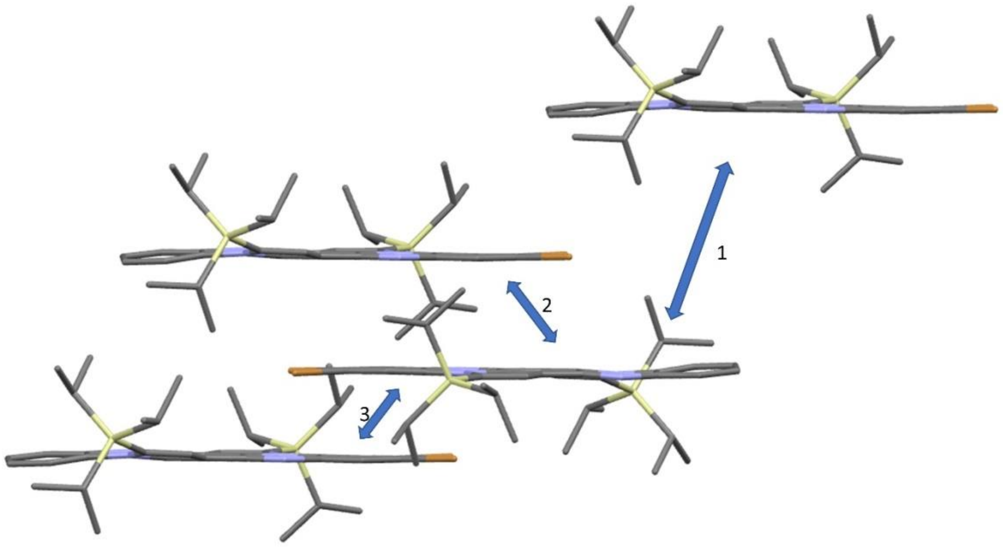

Dimer pair	Transfer integral V (for e^-) [meV]	Reorganisation energy λ [eV]	Electron transfer rate K_{ET} [1/s]
1	0.02		$4.06x10^6$
2	0.86	277	$7.50x10^9$
3	7.85		$6.24x10^{13}$

Figure S46. Top: Dimer Pairs of **3a** used for the calculation of transfer integrals. Bottom: Calculated transfer integrals, reorganization energy and electron transfer rate.

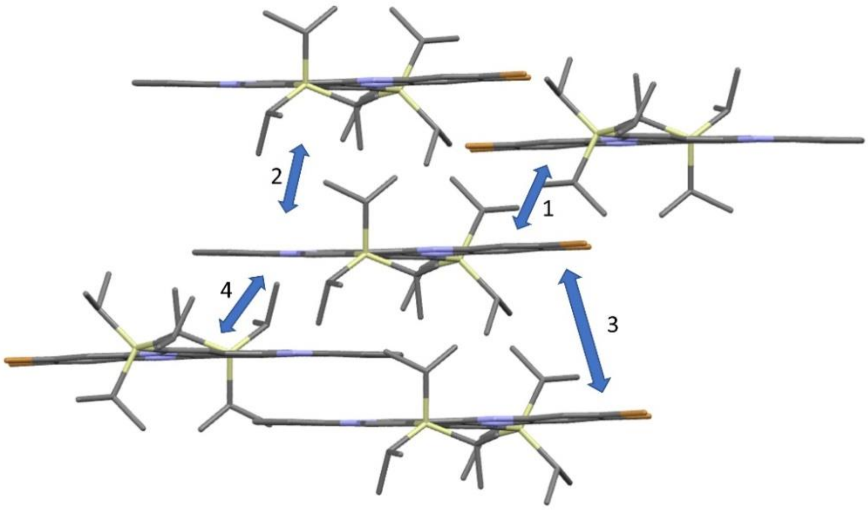

Dimer pair	Transfer integral V (for e^-) [meV]	Reorganisation energy λ [eV]	Electron transfer rate K_{ET} [1/s]
1	30.1		$9.10x10^{12}$
2	0.01	282	$1.01x10^6$
3	0.01		$1.01x10^6$
4	40.6		$1.66x10^{13}$

Figure S47. Top: Dimer Pairs of **3b** used for the calculation of transfer integrals. Bottom: Calculated transfer integrals, reorganization energy and electron transfer rate.

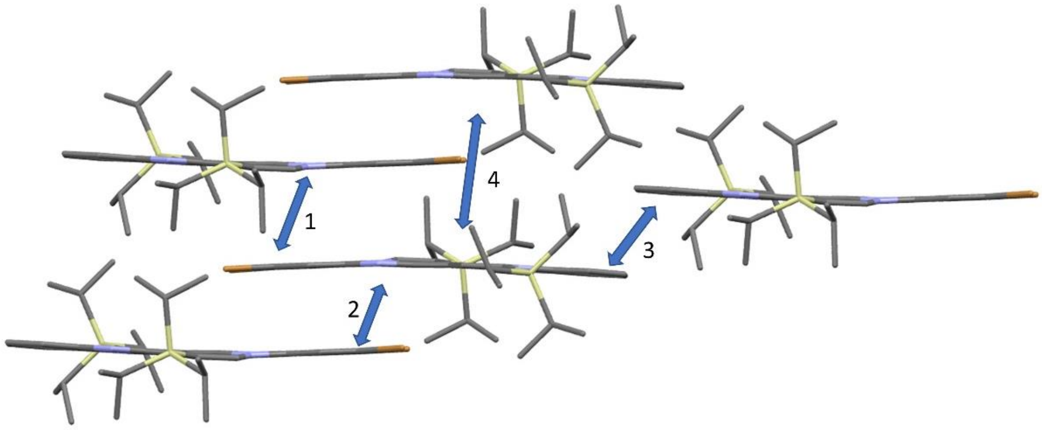

Dimer pair	Transfer integral V (for e$^-$) [meV]	Reorganisation energy λ [eV]	Electron transfer rate K_{ET} [1/s]
1	102	284	$1.02x10^{14}$
2	6.38		$4.08x10^{11}$
3	0.12		$1.44x10^{8}$
4	1.00		$1.00x10^{10}$

Figure S48. Top: Dimer Pairs of **3c** used for the calculation of transfer integrals. Bottom: Calculated transfer integrals, reorganization energy and electron transfer rate.

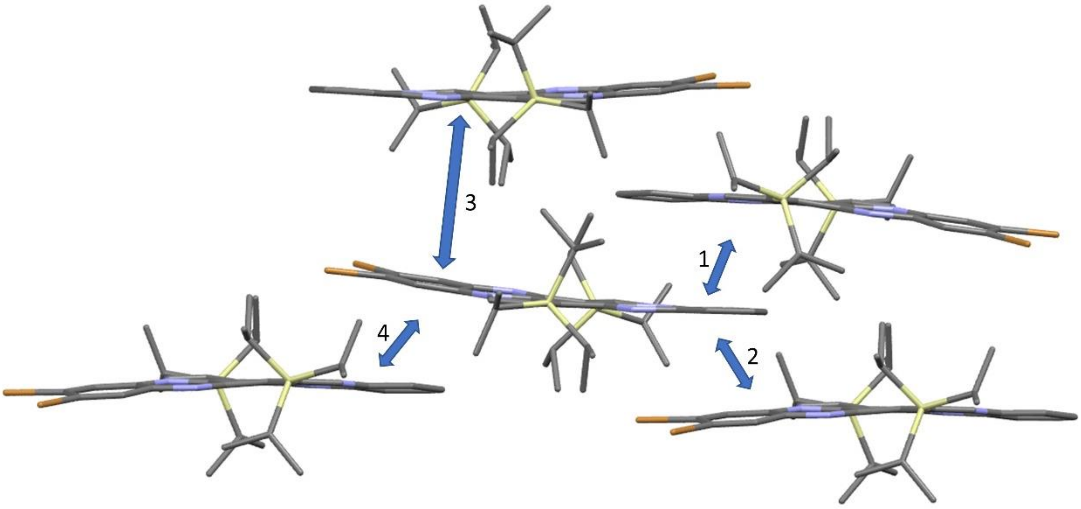

Dimer pair	Transfer integral V (for e$^-$) [meV]	Reorganisation energy λ [eV]	Electron transfer rate K_{ET} [1/s]
1	52.8	288	$2.77x10^{13}$
2	8.45		$7.11x10^{11}$
3	1.67		$2.78x10^{10}$
4	1.67		$2.78x10^{10}$

Figure S49. Top: Dimer Pairs of **3d** used for the calculation of transfer integrals. Bottom: Calculated transfer integrals, reorganization energy and electron transfer rate.

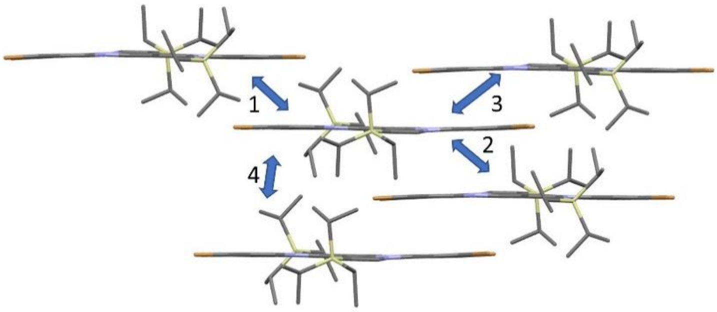

Dimer pair	Transfer integral V (for e^-) [meV]	Reorganisation energy λ [eV]	Electron transfer rate K_{ET} [1/s]
1	13.2		$1.76x10^{12}$
2	84.4	280	$7.18x10^{13}$
3	7.04		$5.00x10^{11}$
4	0.55		$3.05x10^{9}$

Figure S50. Top: Dimer Pairs of **4b** used for the calculation of transfer integrals; Bottom: Calculated transfer integrals, reorganization energy and electron transfer rate.

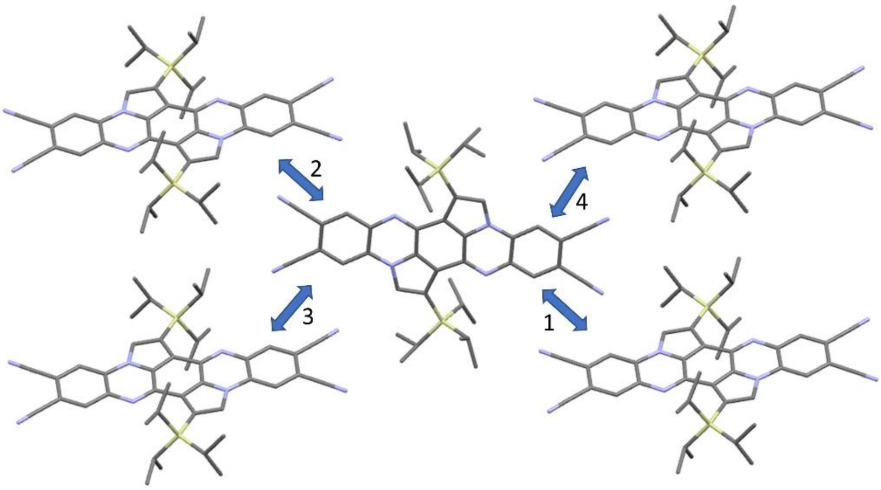

Dimer pair	Transfer integral V (for e^-) [meV]	Reorganisation energy λ [eV]	Electron transfer rate K_{ET} [1/s]
1	3.24		$1.08x10^{11}$
2	3.24	271	$1.08x10^{11}$
3	3.24		$1.08x10^{11}$
4	3.24		$1.08x10^{11}$

Figure S51. Top: Dimer Pairs of **5c** used for the calculation of transfer integrals. Bottom: Calculated transfer integrals, reorganization energy and electron transfer rate.

Afterwards the theoretical electron mobility was calculated assuming a charge carrier diffusion in the crystal. The diffusion coefficient D was calculated as followed:[10,11]

$$D \approx \frac{1}{2n}\sum_i r_i^2 k_{ETi} P_i$$

$$P_i = \frac{k_{ETi}}{\sum_i k_{ETi}}$$

n: Dimension; r_i: intermolecular distance between two molecules; P_i: Hopping probability for path i.

The theoretical electron mobility was calculated using the following formula:[10,12]

$$\mu_{theo} = \frac{e}{k_b T} D$$

The results are shown in the manuscript.

6.1.3 NICS$_{zz}$ XY-Scans

NICS$_{zz}$ XY-Scans were performed using Gaussian 16 starting from the optimized geometries on the B3LYP/def2-TZVP level of theory and the GIAO method. For the scan, every 0.1 Å a dummy atom was placed on a line which is placed in the center of the molecule. To minimize the influence of sigma electron density, the dummy atoms were placed 1.7 Å over the molecule.[13]

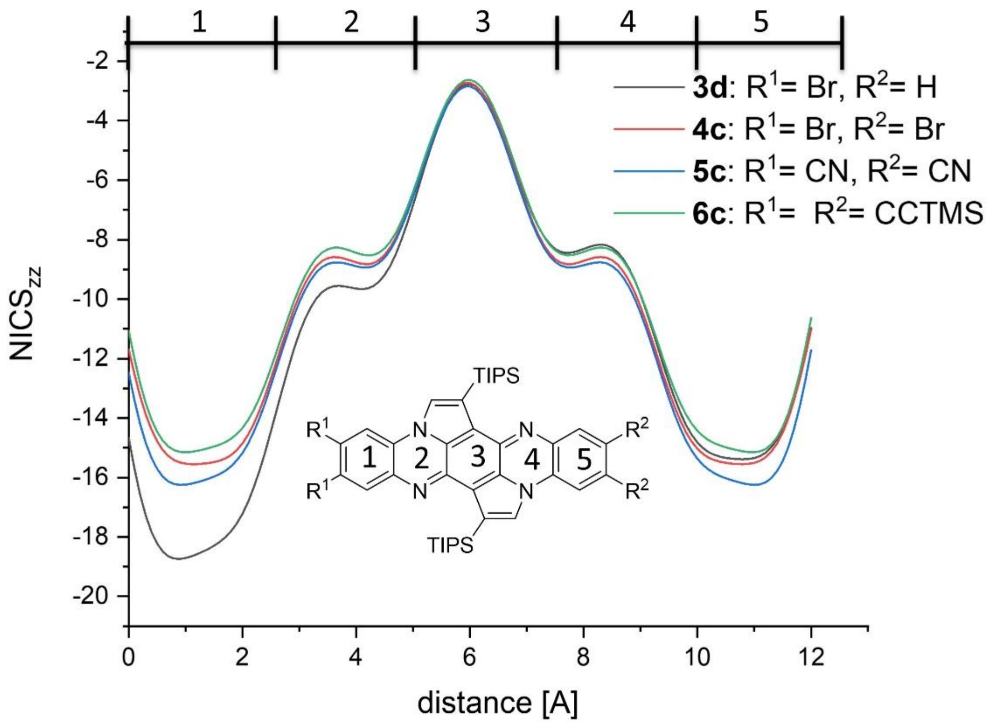

Figure S52. NICS$_{zz}$ XY-Scan of **3d** (black), **4c** (red), **5c** (blue) and **6c** (green).

6.1.4 Coordinates of the Optimized Geometries

1:

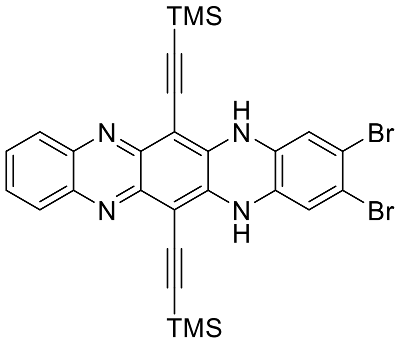

C	7.67852300	0.70844900	0.00026600
C	7.67850300	-0.70866800	0.00023600
C	6.50137100	-1.40688400	0.00017100
C	5.26396400	-0.71538700	0.00013300
C	5.26398400	0.71523600	0.00016300
C	6.50141100	1.40669900	0.00023000
N	4.10866400	-1.40748100	0.00006900
C	2.97868300	-0.72203600	0.00003700
C	2.97870400	0.72195000	0.00006600
N	4.10870400	1.40736400	0.00012700
C	1.72126200	-1.43060700	-0.00003600
C	0.53607200	-0.71762700	-0.00009100
C	0.53609200	0.71761100	-0.00006300
C	1.72130300	1.43055700	0.00002000
N	-0.68147100	-1.35659100	-0.00017800
C	-1.90314300	-0.70277500	-0.00024600
C	-1.90312300	0.70282800	-0.00022000
N	-0.68143300	1.35661000	-0.00012700
C	-3.10928400	-1.38644500	-0.00033700
C	-4.31992100	-0.69499800	-0.00040700
C	-4.31990100	0.69512100	-0.00038200
C	-3.10924400	1.38653200	-0.00028800
C	1.67270600	2.84445800	0.00002700
C	1.54746100	4.05344900	0.00007000
C	1.67262500	-2.84450600	-0.00009700
C	1.54733900	-4.05349300	-0.00009100
Br	-5.92045000	1.71971000	-0.00047400
Br	-5.92049900	-1.71954200	-0.00053500
Si	1.48999500	-5.89402300	0.00037800
C	2.35979600	-6.51389500	1.54426300
C	2.35929800	-6.51466500	-1.54347800
C	-0.31226400	-6.43025300	0.00079500
Si	1.49023000	5.89398200	0.00043700
C	2.36062100	6.51450600	-1.54285200
C	2.35901800	6.51386700	1.54488800
C	-0.31199600	6.43032200	-0.00038100
H	8.62248000	1.23879200	0.00031700
H	8.62244500	-1.23903800	0.00026600
H	6.47733100	-2.48882700	0.00014800
H	6.47740200	2.48864200	0.00025100
H	-0.65756900	-2.36670100	-0.00019900
H	-0.65750200	2.36671900	-0.00011200
H	-3.11403000	-2.46798100	-0.00035700

H	-3.11396000	2.46806900	-0.00027000
H	1.86760500	-6.15044000	2.44859000
H	2.36068100	-7.60659700	1.57669700
H	3.39723100	-6.17477400	1.57060200
H	1.86678200	-6.15171300	-2.44782900
H	2.36022200	-7.60738400	-1.57533100
H	3.39670900	-6.17551100	-1.57035000
H	-0.39002600	-7.52050600	0.00108100
H	-0.83682700	-6.06088800	0.88481200
H	-0.83710200	-6.06133600	-0.88324700
H	1.86868500	6.15156100	-2.44752200
H	2.36165300	7.60722300	-1.57474200
H	3.39802200	6.17526700	-1.56901400
H	1.86620400	6.15046500	2.44889600
H	2.35992900	7.60657000	1.57728700
H	3.39642200	6.17470500	1.57193700
H	-0.83629300	6.06130000	-0.88469900
H	-0.83714600	6.06112700	0.88336000
H	-0.38969200	7.52058000	-0.00030900

1_iso:

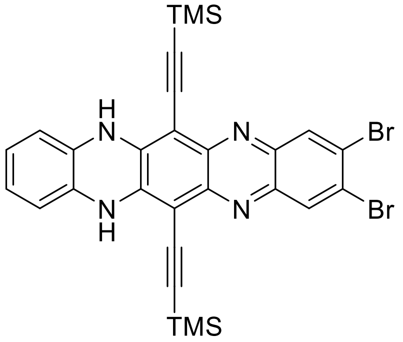

C	-7.56026200	0.69480900	-0.00005500
C	-7.56026400	-0.69478900	-0.00005000
C	-6.35676800	-1.39159700	-0.00003400
C	-5.14982100	-0.70182300	-0.00002400
C	-5.14982000	0.70183700	-0.00002900
C	-6.35676400	1.39161400	-0.00004500
N	-3.92255200	-1.35370500	-0.00000800
C	-2.70859600	-0.71892100	-0.00000900
C	-2.70859400	0.71892800	-0.00001100
N	-3.92254900	1.35371600	-0.00001500
C	-1.52127500	-1.43163800	-0.00000700
C	-0.26539300	-0.72392800	-0.00000300
C	-0.26539100	0.72392900	-0.00000400
C	-1.52127100	1.43164200	-0.00000800
N	0.86439700	-1.40918200	0.00000100
C	2.01747200	-0.71259100	0.00000400
C	2.01747400	0.71258600	0.00000200
N	0.86440100	1.40917900	-0.00000200
C	3.25226500	-1.40112600	0.00000800
C	4.43552300	-0.71095500	0.00001100
C	4.43552500	0.71094300	0.00001000
C	3.25226800	1.40111700	0.00000600
C	-1.56921300	2.84541400	-0.00001000
C	-1.69265600	4.05465700	-0.00000800

C	-1.56922100	-2.84541000	-0.00000900
C	-1.69266800	-4.05465300	-0.00000900
Br	6.05709300	1.70688000	0.00001500
Br	6.05708900	-1.70689600	0.00001800
Si	-1.74962000	-5.89470900	0.00001200
C	-0.88097600	-6.51689600	1.54352700
C	-0.88201900	-6.51692000	-1.54408200
C	-3.55269800	-6.42997500	0.00061000
Si	-1.74959700	5.89471400	0.00000500
C	-0.88074600	6.51693500	-1.54337900
C	-0.88219700	6.51688300	1.54422900
C	-3.55267400	6.42998700	-0.00083000
H	-8.49388100	1.24094500	-0.00006800
H	-8.49388400	-1.24092200	-0.00005700
H	-6.34943500	-2.47495500	-0.00002900
H	-6.34942800	2.47497200	-0.00005000
H	-3.90006800	-2.36398000	-0.00001200
H	-3.90006200	2.36399000	-0.00002400
H	3.23571500	-2.48158500	0.00000900
H	3.23572200	2.48157700	0.00000500
H	-1.37117200	-6.15134300	2.44810000
H	-0.88365100	-7.60958700	1.57641400
H	0.15770700	-6.18156900	1.56908300
H	-1.37286900	-6.15144600	-2.44833200
H	-0.88465500	-7.60961400	-1.57690800
H	0.15662800	-6.18153800	-1.57038300
H	-3.63116300	-7.52018400	0.00065500
H	-4.07651800	-6.06011900	0.88488700
H	-4.07708700	-6.06015500	-0.88334600
H	-1.37088600	6.15149600	-2.44802800
H	-0.88332100	7.60962900	-1.57618100
H	0.15791100	6.18151900	-1.56885900
H	-1.37310100	6.15129400	2.44840400
H	-0.88492900	7.60957300	1.57714200
H	0.15647600	6.18158700	1.57060100
H	-4.07638800	6.06007500	-0.88514600
H	-4.07716900	6.06022700	0.88308700
H	-3.63113500	7.52019700	-0.00095500

2:

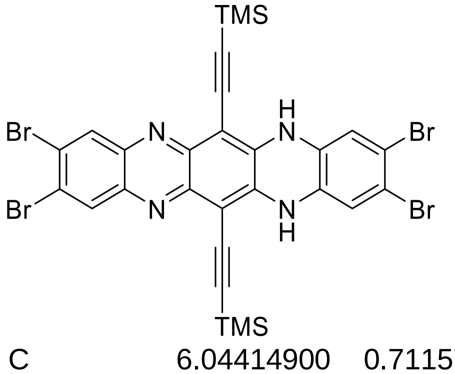

C	6.04414900	0.71157300	0.00009100
C	6.04414900	-0.71157500	0.00009600
C	4.86157100	-1.40177400	0.00006800
C	3.62632800	-0.71297400	0.00003400

C	3.62632800	0.71297300	0.00002800
C	4.86157200	1.40177200	0.00005700
N	2.47416100	-1.40899100	0.00000700
C	1.34408300	-0.72335600	-0.00002400
C	1.34408300	0.72335500	-0.00003000
N	2.47416200	1.40899000	-0.00000400
C	0.08837700	-1.43185000	-0.00005200
C	-1.09693300	-0.71800500	-0.00009000
C	-1.09693300	0.71800500	-0.00009500
C	0.08837800	1.43185000	-0.00006200
N	-2.31290900	-1.35621200	-0.00013300
C	-3.53514500	-0.70251700	-0.00009900
C	-3.53514500	0.70251900	-0.00010500
N	-2.31290900	1.35621300	-0.00014400
C	-4.74098800	-1.38684100	-0.00006900
C	-5.95119200	-0.69538800	-0.00004800
C	-5.95119200	0.69539000	-0.00005500
C	-4.74098700	1.38684300	-0.00008300
C	0.03978600	2.84579500	-0.00006400
C	-0.08436300	4.05478000	-0.00006300
C	0.03978500	-2.84579500	-0.00004300
C	-0.08436600	-4.05478000	-0.00003700
Br	-7.55156400	1.71904500	-0.00002800
Br	-7.55156400	-1.71904200	-0.00001100
Br	7.66555900	1.70607100	0.00012800
Br	7.66555800	-1.70607400	0.00014200
Si	-0.14322900	-5.89704300	-0.00003000
C	0.72542000	-6.51561100	1.54442300
C	0.72553700	-6.51561000	-1.54441800
C	-1.94666200	-6.42841300	-0.00009300
Si	-0.14322400	5.89704300	-0.00002800
C	0.72578100	6.51563600	-1.54427000
C	0.72519400	6.51558400	1.54456700
C	-1.94665700	6.42841400	-0.00034900
H	4.84496200	-2.48221100	0.00007200
H	4.84496400	2.48220900	0.00005300
H	-2.28876800	-2.36657200	-0.00010300
H	-2.28876700	2.36657300	-0.00012400
H	-4.74558400	-2.46837200	-0.00006400
H	-4.74558300	2.46837400	-0.00008800
H	0.23503200	-6.14904700	2.44845400
H	0.72240000	-7.60822500	1.57874200
H	1.76422900	-6.18070300	1.56958200
H	0.23484200	-6.14956100	-2.44849100
H	0.72308300	-7.60823500	-1.57842800
H	1.76416700	-6.18016700	-1.56980300
H	-2.02647400	-7.51849600	-0.00002900
H	-2.47066400	-6.05849400	0.88401800
H	-2.47056400	-6.05860300	-0.88430900
H	0.23526100	6.14955600	-2.44842600
H	0.72328400	7.60826100	-1.57829000
H	1.76443100	6.18023900	-1.56947100
H	0.23463500	6.14906000	2.44852100
H	0.72222700	7.60819900	1.57887100
H	1.76398100	6.18062200	1.56990500
H	-2.47040700	6.05869100	-0.88469100

H	-2.47080900	6.05840800	0.88363600
H	-2.02646900	7.51849700	-0.00018900

3a:

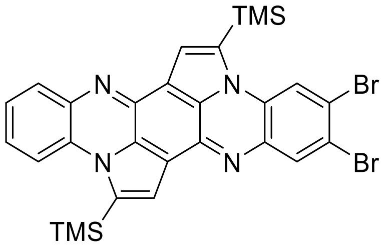

C	7.44609900	1.59933400	-0.00073700
C	7.02181700	2.92976500	-0.00082300
C	5.67336200	3.21372100	-0.00073300
C	4.70556500	2.19390600	-0.00055100
C	5.15382500	0.83930100	-0.00046900
C	6.52211900	0.57000000	-0.00056400
N	3.38188000	2.57718900	-0.00047600
C	2.48964700	1.62513900	-0.00030700
C	2.85831200	0.24701300	-0.00021800
N	4.16982400	-0.16025300	-0.00030900
C	1.05519100	1.82824400	-0.00022700
C	0.22167200	0.72067600	-0.00008200
C	0.59100600	-0.65817600	-0.00001300
C	2.02281000	-0.86107900	-0.00008900
N	-1.09196600	1.13003100	-0.00001700
C	-2.07171900	0.12906900	0.00017500
C	-1.62254900	-1.22385500	0.00023000
N	-0.30247300	-1.61193900	0.00013200
C	-3.43670600	0.39478300	0.00032800
C	-4.36832700	-0.63060400	0.00051000
C	-3.94203100	-1.96316600	0.00054500
C	-2.59044000	-2.23930900	0.00041100
C	2.87714100	-1.98392000	-0.00005100
C	4.20465300	-1.56885900	-0.00021000
C	0.20098500	2.95119200	-0.00030300
C	-1.12709500	2.53726200	-0.00013700
Br	-5.16283800	-3.42115700	0.00078500
Br	-6.20805100	-0.15086100	0.00071200
Si	-2.53830000	3.79864300	-0.00023300
C	-1.72211500	5.49335900	-0.00058800
C	-3.59015300	3.68089600	1.55866200
C	-3.59039800	3.68034800	-1.55892000
Si	5.61299400	-2.83067300	-0.00031500
C	4.79831000	-4.52650600	-0.00047100
C	6.66614400	-2.71832500	1.55911000
C	6.66614900	-2.71802300	-1.55971000
H	8.50241600	1.36377000	-0.00080400
H	7.74741200	3.73260700	-0.00096300
H	5.30950500	4.23248700	-0.00079800
H	6.87235600	-0.44698400	-0.00050100
H	-3.79463800	1.40789700	0.00031900
H	-2.23612100	-3.25984200	0.00044700
H	2.55387400	-3.01062600	0.00006100
H	0.52477600	3.97770800	-0.00044200

H	-1.10308400	5.65483100	-0.88497300
H	-2.49999600	6.26137900	-0.00071800
H	-1.10303800	5.65518000	0.88370200
H	-4.13187400	2.74192400	1.67312100
H	-4.32988700	4.48617400	1.56021000
H	-2.96220600	3.80776900	2.44368200
H	-4.33014900	4.48560900	-1.56062300
H	-2.96259600	3.80693400	-2.44408400
H	-4.13211400	2.74132400	-1.67297000
H	4.17927300	-4.68870900	-0.88471900
H	5.57658600	-5.29419900	-0.00059400
H	4.17935100	-4.68891300	0.88379500
H	7.18351900	-1.76777700	1.68867500
H	7.42287400	-3.50764200	1.54836100
H	6.04112800	-2.87293200	2.44183900
H	7.42282000	-3.50739900	-1.54914100
H	6.04113100	-2.87237100	-2.44248100
H	7.18359500	-1.76747900	-1.68903100

3b:

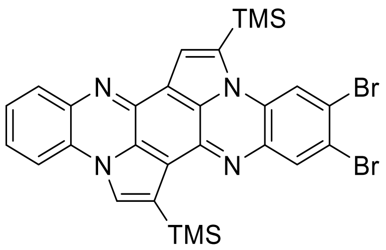

C	8.01496000	0.91557400	0.00000700
C	7.73909000	2.28638200	-0.00000800
C	6.43263800	2.72993400	-0.00001900
C	5.35489100	1.82778600	-0.00001600
C	5.65950700	0.43806200	-0.00000100
C	6.98189500	-0.00433900	0.00001200
N	4.07230200	2.33880900	-0.00002300
C	3.08376400	1.48362400	-0.00001700
C	3.30492600	0.07489700	-0.00001100
N	4.57310400	-0.43725600	-0.00000100
C	1.67696500	1.82233300	-0.00000300
C	0.74631300	0.79363000	0.00002600
C	0.98230600	-0.61833400	0.00003200
C	2.38757400	-0.96938300	0.00000500
N	-0.52388000	1.32627700	0.00002100
C	-1.59723600	0.42748000	0.00001600
C	-1.28284400	-0.96186400	0.00001600
N	-0.00663300	-1.47491800	0.00002300
C	-2.92963300	0.82557800	0.00002200
C	-3.95745900	-0.10335100	0.00002300
C	-3.66371200	-1.47117200	0.00002000
C	-2.34547100	-1.87797600	0.00001800
C	3.13962000	-2.19360800	-0.00004900
C	4.47174100	-1.81389300	0.00001800
C	0.93148000	3.01967200	-0.00002100
C	-0.42835100	2.73080600	0.00004700
Br	-5.02087100	-2.80268000	0.00002300
Br	-5.74113400	0.55416100	0.00003400
Si	-1.71397100	4.12089400	0.00006800

C	-0.74038400	5.73031600	-0.00005900
C	-2.77112800	4.10387000	1.55961700
C	-2.77130000	4.10370600	-1.55936100
Si	2.56226300	-3.98859300	-0.00012100
C	1.55703800	-4.34076200	1.54720400
C	4.10264300	-5.07750700	-0.00008300
C	1.55716700	-4.34069000	-1.54754800
H	9.03958100	0.56752400	0.00001700
H	8.55234500	3.00038100	-0.00001100
H	6.19461100	3.78530300	-0.00002900
H	7.19865400	-1.06392900	0.00002400
H	-3.18687900	1.86865500	0.00003100
H	-2.09563700	-2.92890600	0.00001900
H	5.35404400	-2.42883400	0.00003400
H	1.34989300	4.01132700	-0.00004700
H	-0.10891900	5.83275100	-0.88446300
H	-1.44213600	6.56849400	-0.00003800
H	-0.10879200	5.83281400	0.88424600
H	-3.39799500	3.21981600	1.67637000
H	-3.43233300	4.97477800	1.56060400
H	-2.13291500	4.17304700	2.44370600
H	-3.43250800	4.97461200	-1.56037000
H	-2.13318500	4.17278600	-2.44352800
H	-3.39817200	3.21963200	-1.67594100
H	0.67915800	-3.69496500	1.58957800
H	1.21744000	-5.37990300	1.55922400
H	2.14855200	-4.17265200	2.45014800
H	4.72204300	-4.91145800	-0.88477000
H	3.81339000	-6.13158500	-0.00012200
H	4.72196900	-4.91150300	0.88466400
H	0.67929400	-3.69488900	-1.58996500
H	2.14875900	-4.17254000	-2.45043300
H	1.21756900	-5.37983100	-1.55964500

3c:

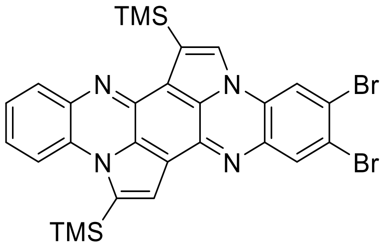

C	-6.91210000	1.81310000	0.00024000
C	-7.14308400	0.43600700	0.00050400
C	-6.08212800	-0.45136000	0.00048800
C	-4.76596200	0.00969800	0.00019600
C	-4.51482700	1.41311600	0.00001100
C	-5.61761200	2.28552000	0.00001300
N	-3.64970100	-0.83846500	0.00013100
C	-2.40627500	-0.25212200	0.00012800
C	-2.23395200	1.16796900	-0.00002200
N	-3.25840300	1.97817200	-0.00012100
C	-1.42866900	-1.23852400	0.00020500
C	-0.04091200	-0.83811100	0.00019200
C	0.11820100	0.57985900	0.00007300
C	-0.84290900	1.58187500	-0.00002700

N	0.98585700	-1.65040500	0.00022700
C	2.24022500	-1.07909500	0.00014900
C	2.48430000	0.32113200	0.00001400
N	1.36414600	1.15004200	-0.00002800
C	3.35608700	-1.92865400	0.00018600
C	4.64638200	-1.43559200	0.00010200
C	4.86348600	-0.05113700	-0.00003000
C	3.78326800	0.81684100	-0.00007600
C	-0.14699000	2.83849200	-0.00004300
C	1.20135500	2.51992100	-0.00019100
C	-2.12018400	-2.46777500	0.00016200
C	-3.49116600	-2.23860500	0.00015800
Br	6.60624200	0.70539300	-0.00015800
Br	6.08264000	-2.68290200	0.00016500
Si	-4.71074600	-3.68463200	-0.00025800
C	-3.66725300	-5.25004600	-0.00071300
C	-5.76800500	-3.71982000	-1.56042900
C	-5.76805500	-3.72085600	1.55986500
Si	-0.80595200	4.60571200	-0.00004900
C	-1.82647700	4.91042900	1.54725200
C	-1.82684100	4.91025300	-1.54714500
C	0.68323400	5.76315900	-0.00029100
H	-7.74415200	2.50495200	0.00024400
H	-8.15517200	0.05271200	0.00073800
H	-6.28451800	-1.50758000	0.00074800
H	-5.40504900	3.34616700	-0.00015000
H	3.17400100	-2.99355900	0.00028500
H	3.96241700	1.88177000	-0.00017900
H	2.05486200	3.17458200	-0.00030300
H	-1.65838700	-3.44002300	0.00013700
H	-3.03172300	-5.32490300	0.88354300
H	-4.33101100	-6.11867400	-0.00098200
H	-3.03169300	-5.32436200	-0.88499300
H	-6.41076000	-2.84954500	-1.69239100
H	-6.40916200	-4.60557800	-1.54916600
H	-5.12647200	-3.78866600	-2.44217600
H	-6.40921800	-4.60660300	1.54797700
H	-5.12653500	-3.79032100	2.44157300
H	-6.41081400	-2.85067600	1.69244500
H	-1.22917000	4.76615200	2.45049200
H	-2.21040100	5.93404400	1.56106400
H	-2.67553300	4.22699500	1.58656100
H	-1.22974000	4.76590300	-2.45051100
H	-2.21079200	5.93385800	-1.56096700
H	-2.67589400	4.22679800	-1.58619300
H	1.30977100	5.62594400	0.88431400
H	1.30957000	5.62582900	-0.88502000
H	0.34633900	6.80298800	-0.00032100

3d:

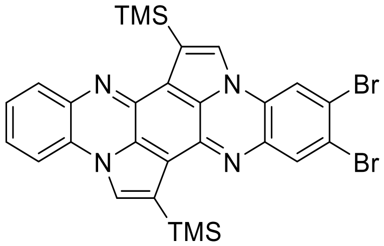

C	7.55257500	0.90981800	-0.00001100
C	7.59117200	-0.48789000	-0.00001000
C	6.41744000	-1.21315000	-0.00000400
C	5.16485700	-0.57517500	0.00000200
C	5.15008300	0.84625700	0.00000100
C	6.33945600	1.57436100	-0.00000700
N	4.02811800	-1.35833500	0.00000500
C	2.86820500	-0.75259400	0.00000800
C	2.77041300	0.67390700	0.00001000
N	3.89510000	1.45407500	0.00000600
C	1.57462200	-1.40651900	0.00001000
C	0.45402300	-0.58462100	0.00000300
C	0.35693800	0.84299800	0.00000200
C	1.64793500	1.49584900	0.00001200
N	-0.67201200	-1.36728200	-0.00001000
C	-1.92351300	-0.75610200	-0.00002000
C	-1.93650900	0.66405300	-0.00001700
N	-0.80472200	1.45026300	-0.00000600
C	-3.11128700	-1.47895300	-0.00003600
C	-4.33074800	-0.82083700	-0.00004700
C	-4.36755600	0.57987100	-0.00004300
C	-3.18763000	1.29825300	-0.00002900
C	2.11298300	2.85475500	0.00003100
C	3.49499100	2.77520500	0.00000000
C	1.11056000	-2.76538900	0.00002200
C	-0.27179900	-2.68753800	-0.00001500
Br	-6.00538900	1.54707800	-0.00005900
Br	-5.90775200	-1.88007000	-0.00006800
Si	1.15322800	4.47806000	0.00006300
C	0.09395800	4.59570400	-1.54665400
C	2.41466000	5.88018800	0.00002800
C	0.09404900	4.59569700	1.54684400
Si	2.07285500	-4.38769500	0.00005700
C	3.13237800	-4.50286100	1.54671900
C	3.13248400	-4.50286500	-1.54653400
C	0.81302400	-5.79095100	0.00001200
H	8.47301400	1.47878300	-0.00001700
H	8.54372400	-1.00147500	-0.00001500
H	6.42519200	-2.29495000	-0.00000400
H	6.31314700	2.65549200	-0.00000900
H	-3.09480000	-2.55865000	-0.00003900
H	-3.20406900	2.37841300	-0.00002800
H	4.22195500	3.56770900	-0.00000800
H	-0.99788700	-3.48115100	-0.00002800
H	-0.62040500	3.77232000	-1.58465700
H	-0.46624200	5.53444400	-1.56135000
H	0.70638600	4.55845700	-2.45052700
H	3.05534000	5.85468800	0.88478700

H	1.90028400	6.84459000	0.00002100
H	3.05531600	5.85466300	-0.88474800
H	-0.46611300	5.53445800	1.56160300
H	-0.62034200	3.77233900	1.58486000
H	0.70652700	4.55839600	2.45068100
H	2.52025900	-4.46519700	2.45077200
H	3.69288500	-5.44145300	1.56203000
H	3.84610100	-3.67883300	1.58293800
H	2.52042300	-4.46525500	-2.45062800
H	3.69302900	-5.44143500	-1.56177600
H	3.84617800	-3.67881100	-1.58273300
H	0.17222400	-5.76653300	0.88471100
H	0.17225400	-5.76650100	-0.88470700
H	1.32844700	-6.75479900	0.00000300

4a:

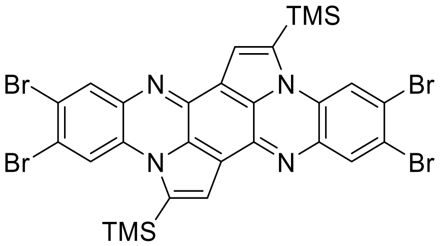

C	-6.00757000	0.19360000	-0.00008900
C	-5.79116200	1.57651800	-0.00007300
C	-4.49821800	2.05664000	-0.00002800
C	-3.38629300	1.20125100	0.00000900
C	-3.62189500	-0.20468800	0.00000700
C	-4.93006700	-0.67684400	-0.00005000
N	-2.14142900	1.78710200	0.00003300
C	-1.11269400	0.98212400	0.00004800
C	-1.26574900	-0.43668900	0.00005100
N	-2.50013100	-1.04290400	0.00004700
C	0.27170300	1.40320200	0.00005400
C	1.26574900	0.43668900	0.00005400
C	1.11269400	-0.98212500	0.00004200
C	-0.27170400	-1.40320200	0.00004500
N	2.50013000	1.04290400	0.00005500
C	3.62189400	0.20468800	0.00002900
C	3.38629200	-1.20125100	0.00001800
N	2.14142900	-1.78710200	0.00002700
C	4.93006600	0.67684400	0.00000800
C	6.00757000	-0.19360000	-0.00001700
C	5.79116100	-1.57651800	-0.00002200
C	4.49821800	-2.05663900	-0.00000500
C	-0.94333300	-2.64455800	0.00003500
C	-2.31860100	-2.43971300	0.00006900
C	0.94333300	2.64455800	0.00004800
C	2.31860100	2.43971300	0.00007400
Br	7.22111400	-2.82891600	-0.00005600
Br	7.75150300	0.56183600	-0.00004300
Br	-7.75150400	-0.56183500	-0.00016500
Br	-7.22111400	2.82891700	-0.00012400
Si	3.52063200	3.90377500	0.00011200
C	2.45267000	5.45171300	0.00006300
C	4.57649800	3.94543100	-1.55943100

C	4.57638500	3.94547100	1.55973300
Si	-3.52063100	-3.90377700	0.00013100
C	-2.45266800	-5.45171300	-0.00003100
C	-4.57659200	-3.94538400	-1.55935000
C	-4.57628900	-3.94552300	1.55981600
H	-4.30446700	3.11941100	-0.00002300
H	-5.12828300	-1.73286000	-0.00007200
H	5.12828200	1.73286100	0.00001000
H	4.30446700	-3.11941100	-0.00001100
H	-0.46579300	-3.60918300	0.00003200
H	0.46579300	3.60918300	0.00004400
H	1.81614400	5.51636800	0.88441300
H	3.10288600	6.33043600	0.00013700
H	1.81628600	5.51641000	-0.88438600
H	5.25338800	3.09883500	-1.67480500
H	5.18650000	4.85291200	-1.56157800
H	3.93572900	3.97659300	-2.44380100
H	5.18644200	4.85291400	1.56185900
H	3.93555100	3.97673800	2.44405200
H	5.25321600	3.09884400	1.67522300
H	-1.81608900	-5.51639600	0.88427800
H	-3.10288300	-6.33043700	0.00005300
H	-1.81633700	-5.51638100	-0.88452100
H	-5.25350400	-3.09879500	-1.67464500
H	-5.18658000	-4.85287500	-1.56149500
H	-3.93587800	-3.97649900	-2.44376100
H	-5.18636000	-4.85295800	1.56194200
H	-3.93540000	-3.97683700	2.44409200
H	-5.25309800	-3.09889100	1.67538500

4b:

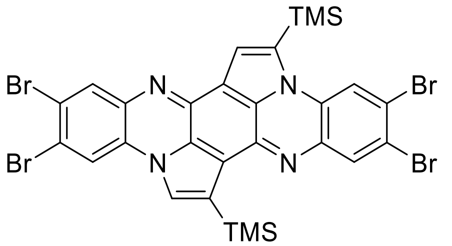

C	5.62657000	-0.27208400	0.00014200
C	5.50817300	1.12258300	0.00010900
C	4.25215800	1.69253900	0.00005900
C	3.08265600	0.91730000	0.00003700
C	3.21866100	-0.50055800	0.00006100
C	4.49017500	-1.06401100	0.00011900
N	1.88122700	1.58682400	-0.00000300
C	0.79281500	0.86207400	-0.00001800
C	0.84862100	-0.56781000	0.00000000
N	2.04070200	-1.25628400	0.00003000
C	-0.55784700	1.38846100	-0.00004200
C	-1.59806500	0.46807900	-0.00004400
C	-1.55559700	-0.95777200	-0.00002500
C	-0.20457300	-1.47111800	-0.00000300
N	-2.79244300	1.13794400	-0.00006400
C	-3.97643900	0.40385500	-0.00006900
C	-3.84827000	-1.01169100	-0.00005100
N	-2.64475200	-1.68318200	-0.00002800

C	-5.23070800	1.00418100	-0.00009200
C	-6.37780100	0.22721200	-0.00009600
C	-6.27495900	-1.17108100	-0.00007800
C	-5.02957600	-1.76780700	-0.00005600
C	0.38410700	-2.75339800	0.00001400
C	1.76895700	-2.63828800	0.00003300
C	-1.14869400	2.69828900	-0.00005700
C	-2.51794800	2.49079800	-0.00007600
Br	-7.80832500	-2.29575100	-0.00008300
Br	-8.05249600	1.12285000	-0.00012800
Br	7.31265600	-1.14856600	0.00022100
Br	7.02252900	2.27116600	0.00013500
Si	-0.34764900	4.40748700	-0.00006700
C	0.69352400	4.62467900	-1.54807900
C	0.69351300	4.62473100	1.54794500
C	-1.73782200	5.68105000	-0.00008200
Si	2.87015300	-4.18033300	-0.00000900
C	1.69995800	-5.65247900	-0.00003500
C	3.92075000	-4.29455300	1.55953900
C	3.92071600	-4.29444300	-1.55958600
H	4.13682100	2.76657400	0.00003700
H	4.61314900	-2.13130700	0.00015100
H	-5.32239700	2.08014700	-0.00010600
H	-4.93487200	-2.84401200	-0.00004200
H	-0.15583300	-3.68444900	0.00000900
H	-3.31510200	3.21303800	-0.00009600
H	0.08534000	4.53142500	-2.45074100
H	1.16201600	5.61236200	-1.56223600
H	1.48275800	3.87308800	-1.58935400
H	0.08535900	4.53132300	2.45061100
H	1.16184600	5.61248800	1.56215500
H	1.48286500	3.87326000	1.58916400
H	-2.37309800	5.59549300	-0.88491500
H	-2.37322200	5.59537700	0.88465100
H	-1.31726600	6.68989000	0.00001700
H	1.06067100	-5.67443700	-0.88446700
H	2.28969500	-6.57287200	-0.00001800
H	1.06062900	-5.67443500	0.88436800
H	4.65730400	-3.49912200	1.67365200
H	4.46353500	-5.24376200	1.56254200
H	3.27990300	-4.27837600	2.44422900
H	4.46350300	-5.24365000	-1.56268000
H	3.27984800	-4.27819500	-2.44426100
H	4.65726200	-3.49899800	-1.67365000

4c:

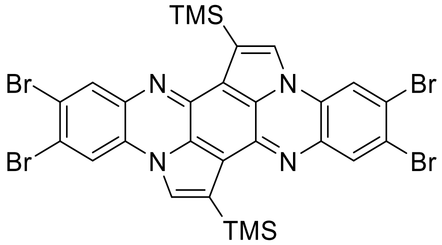

C	5.95507800	0.76447300	0.00006600
C	5.96843900	-0.63738500	0.00005800
C	4.77668900	-1.33533900	0.00002900

C	3.53680100	-0.67932800	0.00000200
C	3.54738600	0.74080300	0.00000900
C	4.74742200	1.44346300	0.00004300
N	2.39169900	-1.44572200	-0.00001900
C	1.24126000	-0.81899700	-0.00003700
C	1.16828400	0.60959300	-0.00004000
N	2.30666900	1.37265300	-0.00001300
C	-0.06141500	-1.45069000	-0.00005200
C	-1.16828400	-0.60959300	-0.00003700
C	-1.24126000	0.81899700	-0.00003000
C	0.06141500	1.45069000	-0.00004900
N	-2.30666900	-1.37265300	-0.00001100
C	-3.54738600	-0.74080300	0.00001500
C	-3.53680100	0.67932800	0.00001300
N	-2.39169900	1.44572200	-0.00000800
C	-4.74742200	-1.44346300	0.00004700
C	-5.95507800	-0.76447300	0.00007500
C	-5.96843900	0.63738500	0.00007200
C	-4.77668900	1.33533900	0.00004300
C	0.54845100	2.80202200	-0.00009100
C	1.92867900	2.70030800	0.00000600
C	-0.54845100	-2.80202200	-0.00009000
C	-1.92867900	-2.70030800	0.00000100
Br	-7.58929700	1.63134800	0.00011300
Br	-7.54956000	-1.79574800	0.00011800
Br	7.54956000	1.79574800	0.00011100
Br	7.58929700	-1.63134800	0.00009400
Si	-0.38482200	4.44293400	-0.00017500
C	-1.44048000	4.57500700	-1.54774400
C	0.90099600	5.82161000	-0.00006400
C	-1.44074400	4.57499500	1.54721600
Si	0.38482200	-4.44293400	-0.00017100
C	1.44073500	-4.57499800	1.54722600
C	1.44048900	-4.57500400	-1.54773400
C	-0.90099600	-5.82161000	-0.00006900
H	4.77445500	-2.41561600	0.00002600
H	4.74956600	2.52325500	0.00005100
H	-4.74956600	-2.52325500	0.00005100
H	-4.77445500	2.41561600	0.00004400
H	2.66838400	3.48117500	0.00003900
H	-2.66838400	-3.48117500	0.00003100
H	-2.16872100	3.76393900	-1.58616900
H	-1.98481200	5.52298500	-1.56351100
H	-0.82801000	4.52739200	-2.45106900
H	1.54098300	5.78580200	0.88480900
H	0.40310400	6.79460200	-0.00011500
H	1.54114500	5.78579100	-0.88481900
H	-1.98506400	5.52298000	1.56290600
H	-2.16900500	3.76393800	1.58550400
H	-0.82843000	4.52735500	2.45064600
H	0.82841600	-4.52736500	2.45065300
H	1.98506000	-5.52298100	1.56291400
H	2.16899300	-3.76393800	1.58552200
H	0.82802400	-4.52738700	-2.45106300
H	1.98482100	-5.52298200	-1.56350000
H	2.16873000	-3.76393600	-1.58615400

H	-1.54099000	-5.78580200	0.88480000
H	-1.54113900	-5.78579200	-0.88482900
H	-0.40310500	-6.79460200	-0.00011600

5c:

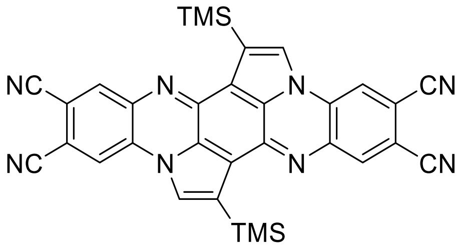

C	-5.94204900	0.87543700	-0.00000700
C	-5.97945900	-0.54098000	-0.00000600
C	-4.79335000	-1.25737300	-0.00000300
C	-3.54437500	-0.61924700	-0.00000100
C	-3.53058800	0.80479100	-0.00000200
C	-4.71652900	1.53050800	-0.00000500
N	-2.41559900	-1.40303600	0.00000100
C	-1.25278500	-0.79627000	0.00000200
C	-1.15581400	0.63001900	0.00000200
N	-2.28163600	1.41291600	-0.00000100
C	0.03610300	-1.45233700	0.00000300
C	1.15581400	-0.63001900	0.00000200
C	1.25278500	0.79627000	0.00000000
C	-0.03610300	1.45233700	0.00000200
N	2.28163600	-1.41291600	-0.00000100
C	3.53058800	-0.80479100	-0.00000400
C	3.54437500	0.61924700	-0.00000500
N	2.41559900	1.40303500	-0.00000300
C	4.71652900	-1.53050800	-0.00000700
C	5.94204900	-0.87543700	-0.00000900
C	5.97945900	0.54098000	-0.00001100
C	4.79335000	1.25737300	-0.00000800
C	-0.49916400	2.81363000	0.00000500
C	-1.87920500	2.73569600	-0.00000300
C	0.49916400	-2.81363000	0.00000700
C	1.87920500	-2.73569600	-0.00000100
Si	0.46037100	4.44608700	0.00000900
C	1.51382200	4.55253600	1.54958000
C	-0.80907800	5.83672100	-0.00000100
C	1.51384300	4.55252700	-1.54954800
Si	-0.46037100	-4.44608700	0.00001300
C	-1.51384300	-4.55253000	-1.54954400
C	-1.51382200	-4.55253300	1.54958400
C	0.80907800	-5.83672100	0.00000500
C	7.22396500	1.24239200	-0.00001500
N	8.21483600	1.82967600	-0.00001300
C	7.14139100	-1.64839000	-0.00001100
N	8.09215400	-2.29930800	-0.00001900
C	-7.14139100	1.64839000	-0.00001000
N	-8.09215400	2.29930700	-0.00001100
C	-7.22396500	-1.24239200	-0.00000800
N	-8.21483600	-1.82967600	-0.00001000
H	-4.80978000	-2.33787800	-0.00000300
H	-4.70028800	2.61059200	-0.00000600

H	4.70028800	-2.61059200	-0.00000600
H	4.80978000	2.33787800	-0.00000900
H	-2.60463200	3.52987200	-0.00000700
H	2.60463200	-3.52987200	-0.00000300
H	2.23046700	3.73135600	1.58933300
H	2.07265600	5.49181000	1.56884200
H	0.89910200	4.51336800	2.45166400
H	-1.44905800	5.81037300	-0.88514600
H	-0.29897700	6.80324900	0.00001500
H	-1.44908700	5.81035900	0.88512300
H	2.07265300	5.49181500	-1.56882400
H	2.23050800	3.73136400	-1.58927100
H	0.89913700	4.51332100	-2.45164000
H	-0.89913600	-4.51333600	-2.45163600
H	-2.07266100	-5.49181300	-1.56881400
H	-2.23050100	-3.73136100	-1.58927400
H	-0.89910200	-4.51335000	2.45166800
H	-2.07264600	-5.49181200	1.56885400
H	-2.23047500	-3.73136000	1.58932900
H	1.44906300	-5.81037000	-0.88513700
H	1.44908300	-5.81036100	0.88513200
H	0.29897700	-6.80324900	0.00001500

6c:

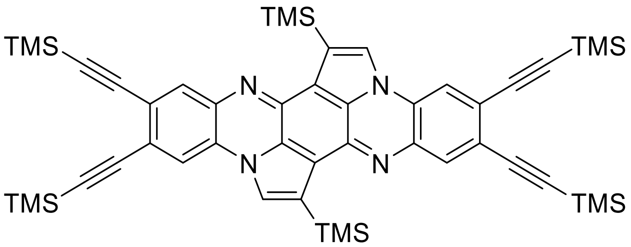

C	5.98156000	-0.68304900	-0.00201100
C	5.97613200	0.74255700	-0.00156900
C	4.75502200	1.41612200	-0.00212800
C	3.55355000	0.72094500	-0.00293800
C	3.53453200	-0.70175200	-0.00319600
C	4.76924600	-1.36328900	-0.00281900
N	2.31507100	1.35846400	-0.00331700
C	1.17200900	0.60296700	-0.00350100
C	1.23629100	-0.82562400	-0.00364900
N	2.38366800	-1.45908700	-0.00361100
C	0.06971900	1.45042600	-0.00353300
C	-1.23647800	0.82702500	-0.00342300
C	-1.17228900	-0.60156100	-0.00367100
C	-0.06998700	-1.44903900	-0.00379100
N	-2.38376000	1.46059200	-0.00294000
C	-3.53466000	0.70339200	-0.00264400
C	-3.55392300	-0.71930500	-0.00310000
N	-2.31543900	-1.35696800	-0.00363500
C	-4.76915200	1.36523800	-0.00163700
C	-5.98162700	0.68537200	-0.00111100
C	-5.97659700	-0.74023300	-0.00186300
C	-4.75562200	-1.41416100	-0.00280900
C	-0.56590400	-2.79734400	-0.00384400
C	-1.94560200	-2.68686200	-0.00376800
C	0.56551600	2.79877200	-0.00348600

C	1.94520000	2.68836300	-0.00333600
C	-7.18341900	-1.48755600	-0.00136800
C	-7.19925100	1.41921700	0.00031500
C	7.19957400	-1.41635800	-0.00125100
C	7.18265900	1.49022600	-0.00040800
C	-8.19828900	-2.15289200	-0.00116500
C	-8.22138600	2.07222700	0.00174300
C	8.19729300	2.15585200	0.00074800
C	8.22224800	-2.06859000	-0.00063100
Si	0.35840300	-4.44153100	-0.00382600
C	1.41451300	-4.58177600	1.54310800
C	1.41439200	-4.58198000	-1.55078500
C	-0.93363000	-5.81539000	-0.00359100
Si	-0.35892100	4.44293900	-0.00390900
C	-1.41492600	4.58363600	1.54303200
C	-1.41499400	4.58264700	-1.55089800
C	0.93299700	5.81686600	-0.00439000
Si	-9.74075700	-3.15677900	0.00338900
C	-10.03632000	-3.78397600	1.74947800
C	-9.51385100	-4.59714900	-1.18130100
C	-11.16400000	-2.06534500	-0.55309500
Si	-9.77103700	3.06387700	0.00426700
C	-10.76598500	2.62102700	-1.52620300
C	-9.30825700	4.88443800	-0.01653900
C	-10.73988800	2.65122800	1.56023000
Si	9.74389100	3.15371300	0.00307400
C	9.27329000	4.97244100	-0.02304100
C	10.74110200	2.71013600	-1.52519600
C	10.71014900	2.74805800	1.56200100
Si	9.76881200	-3.06467300	0.00472300
C	10.06591200	-3.69403400	1.74985500
C	11.18926500	-1.96594300	-0.54671100
C	9.55243900	-4.50359000	-1.18348100
H	4.76052900	2.49656500	-0.00178300
H	4.75959000	-2.44405700	-0.00309300
H	-4.75924600	2.44601100	-0.00114500
H	-4.76137200	-2.49459900	-0.00329600
H	-2.69057900	-3.46257400	-0.00380600
H	2.69015200	3.46410200	-0.00330100
H	0.80286700	-4.53053500	2.44682400
H	1.95316800	-5.53306300	1.55801600
H	2.14741900	-3.77485400	1.58055300
H	0.80266500	-4.53087800	-2.45445400
H	1.95308900	-5.53324100	-1.56563700
H	2.14728900	-3.77505900	-1.58841700
H	-1.57355800	-5.77609200	0.88119100
H	-1.57377800	-5.77624800	-0.88821700
H	-0.44014900	-6.79067800	-0.00357100
H	-0.80316900	4.53295500	2.44670700
H	-1.95381000	5.53479500	1.55752700
H	-2.14765900	3.77658100	1.58090000
H	-0.80327700	4.53130600	-2.45456400
H	-1.95381500	5.53383100	-1.56602700
H	-2.14779400	3.77562500	-1.58819100
H	0.43941500	6.79210300	-0.00470500
H	1.57308400	5.77803600	0.88029200

H	1.57304100	5.77743800	-0.88907500
H	-10.14555500	-2.95552300	2.45225600
H	-10.94720800	-4.38670400	1.79516200
H	-9.20573400	-4.40457600	2.09198900
H	-9.31920300	-4.24576100	-2.19663000
H	-10.41083300	-5.22140100	-1.20781900
H	-8.67538400	-5.22835200	-0.87958600
H	-11.29450600	-1.21663200	0.12119200
H	-10.98938500	-1.67217800	-1.55659500
H	-12.10070800	-2.62849100	-0.57001500
H	-11.01881300	1.55896500	-1.53722900
H	-11.69884700	3.18996200	-1.56020100
H	-10.20516900	2.84000000	-2.43714200
H	-8.71108300	5.14944100	0.85842000
H	-10.20323500	5.51210900	-0.01502700
H	-8.72581600	5.13280400	-0.90618800
H	-10.99003900	1.58910500	1.59805600
H	-10.16479200	2.89001400	2.45715600
H	-11.67352900	3.21861400	1.59776900
H	8.67432100	5.23782600	0.85061800
H	10.16586100	5.60348400	-0.02178800
H	8.69175800	5.21676900	-0.91444200
H	10.99663400	1.64874600	-1.53325200
H	11.67248700	3.28147200	-1.55913100
H	10.18103400	2.92551100	-2.43743100
H	11.64242000	3.31769200	1.59928300
H	10.13313300	2.98815900	2.45732300
H	10.96251500	1.68663800	1.60318100
H	9.23700700	-4.31811000	2.09007300
H	10.97896100	-4.29350700	1.79548900
H	10.17112500	-2.86654900	2.45443700
H	11.01480200	-1.57130200	-1.54970700
H	12.12838300	-2.52508400	-0.56337900
H	11.31533600	-1.11825500	0.12981100
H	10.45184600	-5.12445300	-1.20820100
H	8.71496400	-5.13794400	-0.88579100
H	9.35946800	-4.15091500	-2.19868800

7 Device Fabrication and Measurement

A sliced, highly p-doped silicon wafer with 100 nm thick thermally grown SiO_2 was cleaned with ultra-sonication in acetone, isopropanol and ethanol successively for 10 min. It was washed with water and dried in a stream of nitrogen. The wafer was placed in freshly produced Caro´s acid and got heated to 100 °C for 20 min. After cleaning with water and drying a 150 mM solution of $Al(NO_3)_3$*$9H_2O$ in ethanol was spin-coated (5000 rpm; 40 s) onto the substrate. Right after that, the wafer was heated to 300 °C for 30 min. For the formation of the SAM the substrate was placed in a 15.0 mM solution of 12-cyclohexyldodecylphosphonic acid (CDPA)[12] in isopropanol for 16 h. Then, the substrate was cleaned with ultra-sonication in isopropanol for 10 min, rinsed with water and dried in a stream of nitrogen. The capacitance of the dielectric layer is 26.5 nF cm^{-2}.
Drop-casted films were prepared by dropping the prepared solution onto the substrate and covering the wafer against drafts. To form the electrodes, a 40 nm thick layer of gold was deposited through a shadow mask onto the organic layer in a vacuum evaporator at a pressure below $2x10^{-6}$ bar. Transistor characteristics were measured with a semiconductor characterization system (Keithley 4200-SCS) in a nitrogen filled glove box. The field effect mobilities were determined in the saturated regime using the equation $I_{DS}=(W/2L)C_i\mu\ (V_G-V_{th})^2$, where I_{DS} is the source-drain current, W is the channel width, L the channel length, C_i is the capacitance per unit area of the gate dielectric layer, µ is the field effect mobility, and V_{th} is the threshold voltage.

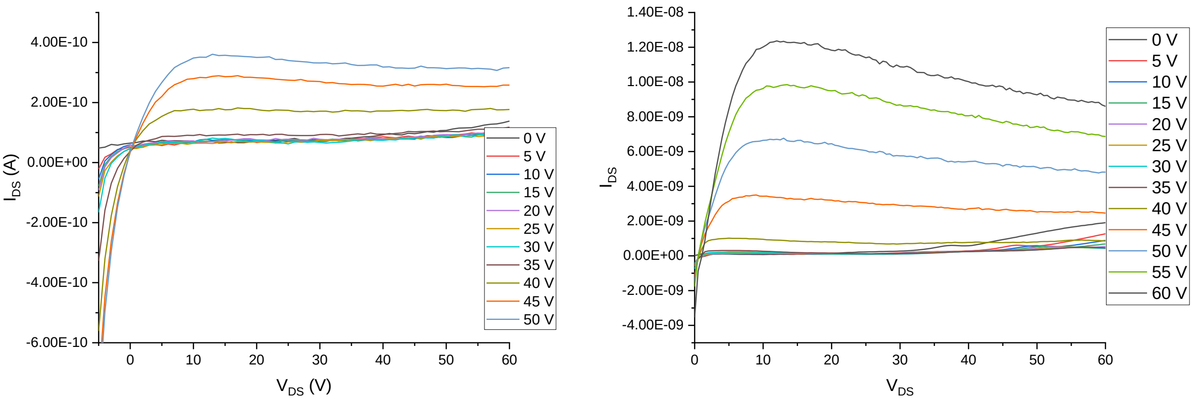

Figure S53. Output characteristics of **5c** (left) and **3d** (right).

8 Stability Measurements

Stabilities of the compound were measured by time dependent absorption spectroscopy. The solutions of the compounds in DCM were irradiated with UV light (365nm) and white light under ambient conditions. In the figure below our LED panel is shown. The yellow LED were used for the measurement. All other LED were switched off.

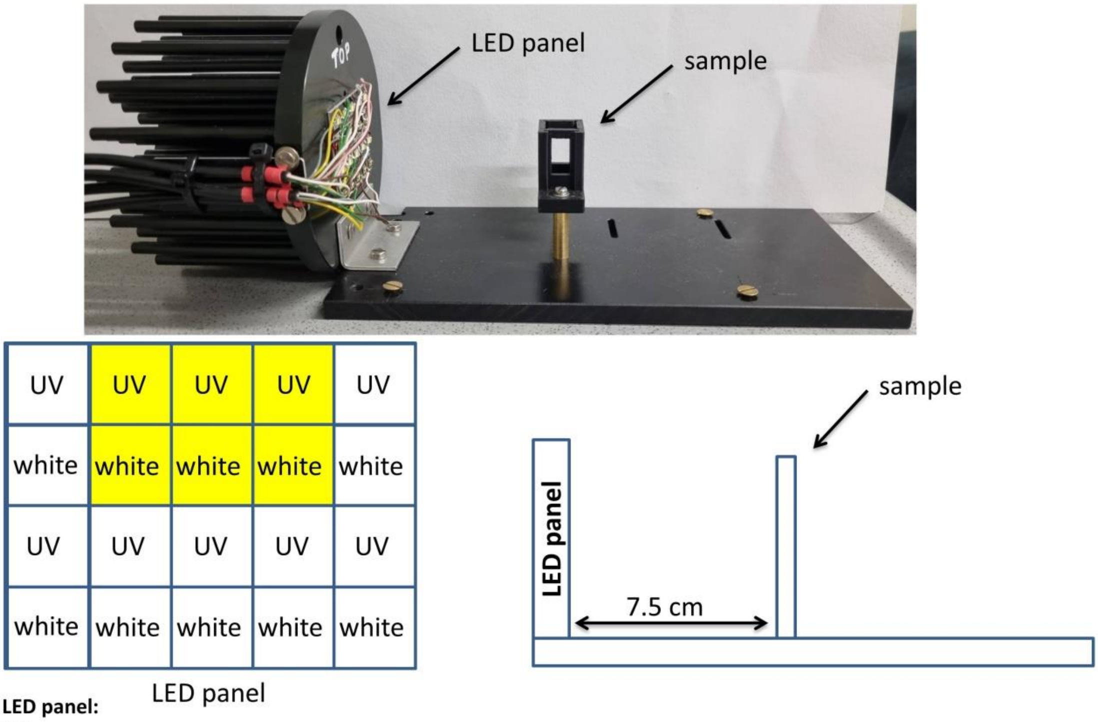

LED panel:
UV:
UV-LED: Nichia NCSU276C UV SMD-LED, 10x10mm Platine, 1050mW, 365nm

white:
white light LED: Cree XP-G2 S2 SMD-LED, 10x10mm Platine, 338lm, 6200K, CRI 70

Figure S54. Photograph and schematic structure of the LED pane which was used for the stability measurements.

9 References

[1] G. R. Fulmer, A. J. M. Miller, N. H. Sherden, H. E. Gottlieb, A. Nudelman, B. M. Stoltz, J. E. Bercaw, K. I. Goldberg, *Organometallics* **2010**, *29*, 2176–2179.

[2] B. D. Lindner, J. U. Engelhart, O. Tverskoy, A. L. Appleton, F. Rominger, A. Peters, H.-J. Himmel, U. H. F. Bunz, *Angew. Chem. Int. Ed.* **2011**, *50*, 8588–8591; *Angew. Chem.* **2011**, *123*, 8747–8750.

[3] M. J. Fleming, H. A. McManus, A. Rudolph, W. H. Chan, J. Ruiz, C. Dockendorff, M. Lautens, *Chem. Eur. J.* **2008**, *14*, 2112–2124.

[4] H. Reiss, L. Ji, J. Han, S. Koser, O. Tverskoy, J. Freudenberg, F. Hinkel, M. Moos, A. Friedrich, I. Krummenacher, C. Lambert, H. Braunschweig, A. Dreuw, T. B. Marder, U. H. F. Bunz, *Angew. Chem. Int. Ed.* **2018**, *57*, 9543–9547; *Angew. Chem.* **2018**, *130*, 9688–9692.

[5] J. U. Engelhart, F. Paulus, M. Schaffroth, V. Vasilenko, O. Tverskoy, F. Rominger, U. H. F. Bunz, *J. Org. Chem.* **2016**, *81*, 1198–1205.

[6] *Gaussian 16*, *Revision B.01*, M. J. Frisch, G. W. Trucks, H. B. Schlegel, G. E. Scuseria, M. A. Robb, J. R. Cheeseman, G. Scalmani, V. Barone, G. A. Petersson, H. Nakatsuji, X. Li, M. Caricato, A. V. Marenich, J. Bloino, B. G. Janesko, R. Gomperts, B. Mennucci, H. P. Hratchian, J. V. Ortiz, A. F. Izmaylov, J. L. Sonnenberg, D. Williams-Young, F. Ding, F. Lipparini, F. Egidi, J. Goings, B. Peng, A. Petrone, T. Henderson, D. Ranasinghe, V. G. Zakrzewski, J. Gao, N. Rega, G. Zheng, W. Liang, M. Hada, M. Ehara, K. Toyota, R. Fukuda, J. Hasegawa, M. Ishida, T. Nakajima, Y. Honda, O. Kitao, H. Nakai, T. Vreven, K. Throssell, J. A. Montgomery, Jr., J. E. Peralta, F. Ogliaro, M. J. Bearpark, J. J. Heyd, E. N. Brothers, K. N. Kudin, V. N. Staroverov, T. A. Keith, R. Kobayashi, J. Normand, K. Raghavachari, A. P. Rendell, J. C. Burant, S. S. Iyengar, J. Tomasi, M. Cossi, J. M. Millam, M. Klene, C. Adamo, R. Cammi, J. W. Ochterski, R. L. Martin, K. Morokuma, O. Farkas, J. B. Foresman, and D. J. Fox, Gaussian, Inc., Wallingford CT, **2016**.

[7] a) ADF 2021.1, SCM, Theoretical Chemistry, Vrije Universiteit, Amsterdam, The Netherlands, E.J. Baerends, T. Ziegler, A.J. Atkins, J. Autschbach, O. Baseggio, D. Bashford, A. Bérces, F.M. Bickelhaupt, C. Bo, P.M. Boerrigter, C. Cappelli, L. Cavallo, C. Daul, D.P. Chong, D.V. Chulhai, L. Deng, R.M. Dickson, J.M. Dieterich, F. Egidi, D.E. Ellis, M. van Faassen, L. Fan, T.H. Fischer, A. Förster, C. Fonseca Guerra, M. Franchini, A. Ghysels, A. Giammona, S.J.A. van Gisbergen, A. Goez, A.W. Götz, J.A. Groeneveld, O.V. Gritsenko, M. Grüning, S. Gusarov, F.E. Harris, P. van den Hoek, Z. Hu, C.R. Jacob, H. Jacobsen, L. Jensen, L. Joubert, J.W. Kaminski, G. van Kessel, C. König, F. Kootstra, A. Kovalenko, M.V. Krykunov, P. Lafiosca, E. van Lenthe, D.A. McCormack, M. Medves, A. Michalak, M. Mitoraj, S.M. Morton, J. Neugebauer, V.P. Nicu, L. Noodleman, V.P. Osinga, S. Patchkovskii, M. Pavanello, C.A. Peeples, P.H.T. Philipsen, D. Post, C.C. Pye, H. Ramanantoanina, P. Ramos, W. Ravenek, M. Reimann, J.I. Rodríguez, P. Ros, R. Rüger, P.R.T. Schipper, D. Schlüns, H. van Schoot, G. Schreckenbach, J.S. Seldenthuis, M. Seth, J.G. Snijders, M. Solà, M. Stener, M. Swart, D. Swerhone, V. Tognetti, G. te Velde, P. Vernooijs, L. Versluis, L. Visscher, O. Visser, F. Wang, T.A. Wesolowski, E.M. van Wezenbeek, G. Wiesenekker, S.K. Wolff, T.K. Woo, A.L. Yakovlev; b) A. Moliterni, D. Altamura, R. Lassandro, V. Olieric, G. Ferri, F. Cardarelli, A. Camposeo, D. Pisignano, J. E. Anthony, C. Giannini, *Acta Crystallogr. B* **2020**, *76*, 427–435; c) G. te Velde, F. M. Bickelhaupt, E. J. Baerends, C. Fonseca Guerra, S. J. A. van Gisbergen, J. G. Snijders, T. Ziegler, *J. Comput. Chem.* **2001**, *22*, 931–967.

[8] V. Stehr, J. Pfister, R. F. Fink, B. Engels, C. Deibel, *Phys. Rev. B* **2011**, *83*, 155208.

[9] R. A. Marcus, *Rev. Mod. Phys.* **1993**, *65*, 599–610.

[10] S.-H. Wen, an Li, J. Song, W.-Q. Deng, K.-L. Han, W. A. Goddard, *J. Phys. Chem. B* **2009**, *113*, 8813–8819.

[11] A. N. Sokolov, S. Atahan-Evrenk, R. Mondal, H. B. Akkerman, R. S. Sánchez-Carrera, S. Granados-Focil, J. Schrier, S. C. Mannsfeld, A. P. Zoombelt, Z. Bao, A. Aspuru-Guzik, *Nat. Commun.* **2011**, *2*, 437.

[12] M. Chu, J.-X. Fan, S. Yang, D. Liu, C. F. Ng, H. Dong, A.-M. Ren, Q. Miao, *Adv. Mater.* **2018**, *30*, e1803467.

[13] A. Stanger, *Org. Lett.* **2022**, *24*, 1243–1246.
